# Reactivity of Low Valent Magnesium(I) Complexes with Epoxides and Episulfides

**DOI:** 10.1002/anie.202520259

**Published:** 2025-11-02

**Authors:** Peter J. Shaw, Gary S. Nichol, Jennifer A. Garden

**Affiliations:** ^1^ School of Chemistry University of Edinburgh Edinburgh EH9 3FJ UK

**Keywords:** Cleavage reactions, Magnesium, Subvalent compounds

## Abstract

Chemical C–H functionalisation is traditionally the domain of potent organolithium bases that require cryogenic conditions, whereas biologically inspired C–H functionalisation can be executed by transition metal‐oxo species. Blending these chemical and biological approaches, this work exploits magnesium‐oxo complexes for selective C–H deprotonation at ambient temperature. Overall, three low valent magnesium(I) dimers, [^Dipp^NacnacMg]_2_ **1**, [^Dep^NacnacMg]_2_ **2** and [^Mes^NacnacMg]_2_ **3**, react with two equivalents of propylene oxide to generate dinuclear Mg complexes of general formula [NacnacMg(OH)(OCH_2_CHCH_2_)MgNacnac] (Nacnac = HC(C(CH_3_)NAr)_2_). Isolation of oxo‐bridged [^Dipp^NacnacMg‐O‐Mg^Dipp^Nacnac] coupled with deuterium labeling studies shows that the reaction proceeds via epoxide deoxygenation, followed by deprotonation and rearrangement of a second equivalent of the epoxide to generate the alkoxide. A range of terminal and internal epoxides was investigated (propylene oxide, butylene oxide, isobutylene oxide, and cyclohexene oxide). The β‐C‐H position was selectively deprotonated in each case, as confirmed by NMR spectroscopy and X‐ray diffraction studies. Exchanging epoxides for episulfides instead generated disulfide‐bridged species, showcasing the difference in Brønsted basicity between Mg‐O and Mg‐S functional groups. Overall, this work demonstrates the unharnessed potential of main group metal‐oxo complexes for selective C–H deprotonation.

## Graphical abstract

C–H functionalisation and ether cleavage are typically performed by aggressive organolithium reagents. Unveiling the untapped Brønsted basicity of magnesium‐oxo species, the reaction of Mg(I) precursors with epoxides initially proceeds via deoxygenation, followed by deprotonation and rearrangement of a second epoxide. Episulfides form a bridged chalcogen species initially, but ultimately show divergent reactivity to the disulfide‐bridged product.

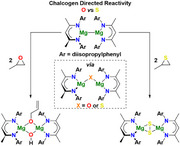

## Introduction

**Scheme 1:**
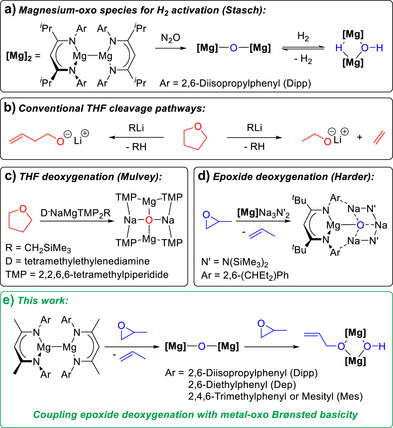
a) Reversible reaction of an oxo‐bridged magnesium complex with H_2_ to generate a dinuclear Mg complex with a hydroxide and hydride bridge; b) conventional ether cleavage pathways to generate lithium alkoxides; c) deoxygenation of THF to form an inverse crown oxo‐complex; d) epoxide deoxygenation to generate an inverse crown oxo‐complex; e) epoxide deoxygenation and metal‐oxo Brønsted basicity explored in this work.

C–H functionalisation underpins the synthesis of pharmaceuticals, agrochemicals, and advanced materials, with highly reactive organolithium bases reportedly used in over 95% of natural product syntheses.^[^ ^1^ ^]^ Yet lithium is under rising threat from increasing use, particularly within lithium ion batteries.^[^ ^2^ ^]^ Nature‐inspired C–H functionalisation has been achieved using high valent transition metal‐oxo (M^n^^+^ = O) compounds.^[^ ^3^ ^]^ However, the use of main group metal‐oxo complexes for C–H functionalisation remains underexplored. As the diagonal neighbour of lithium, magnesium‐oxo Brønsted bases are particularly attractive due to the low toxicity, Earth abundance and prevalence of magnesium in C–H functionalisation. Stasch and co‐workers recently reported the reduction of magnesium‐oxo species using hydrogen, to reversibly form mixed hydride/hydroxide complexes (Scheme 1a).^[^ ^4^ ^]^ This elegant study showed that magnesium‐oxo units can act as Brønsted bases to generate Mg–OH species. While organometallic chemists often dismiss the formation of metal hydroxides as decomposition products due to adventitious water, these studies show that metal‐oxo species can perform alternative deprotonative pathways.

Oxo‐bridged metal complexes (M‐O‐M) are often generated through hydrolytic pathways, such as the reaction between a metal‐hydroxide (M‐OH) and a metal‐alkyl or metal‐amide (MR or MNR_2_).^[^ ^5^ ^]^ Other routes include the use of low‐valent metal complexes to deoxygenate small molecules such as N_2_O.^[^ ^6^, ^7^ ^]^ Alternatively, metal‐oxo species can be generated via the deoxygenation of ethers such as THF and epoxides. It is worth noting that deoxygenation is not the typical pathway for ether cleavage. The reaction of THF with organolithium bases can follow multiple pathways, yet typically deprotonation and rearrangement to generate lithium alkoxides occurs (Scheme 1b).^[^ ^8^, ^9^ ^]^ In 2009, Mulvey and co‐workers reported the α‐deprotonation and stabilisation of THF using a heterometallic sodium zincate base, [(TMEDA)Na(TMP)(CH_2_SiMe_3_)Zn(TMP)] (TMEDA = N,N,N’,N’‐tetramethylethylenediamine; TMP = 2,2,6,6‐tetramethylpiperidine),^[^ ^10^ ^]^ a concept that has since been extended to pyrrolidine analogues.^[^ ^11^ ^]^ In 2010, Mulvey and co‐workers deoxygenated THF using the closely related sodium magnesiate base, [(TMEDA)Na(TMP)(CH_2_SiMe_3_)Mg(TMP)],^[^ ^9^, ^12^ ^]^ and the oxo O^2−^ anion was encapsulated within a tetrametallic “inverse crown” structure (Scheme 1c).

Recently, Harder and co‐workers demonstrated that a tetrametallic Mg/Na_3_ complex could deoxygenate epoxides,^[^ ^13^ ^]^ as the three‐membered and highly ring‐strained analogues of THF (Scheme 1d). This included propylene oxide (PO), 1‐hexylene oxide, and styrene oxide as well as *cis*‐ and *trans*‐stillbene oxide. This reaction also generated an “inverse crown” featuring a bridging O^2−^ unit. Deoxygenation of epoxides to alkenes is an intriguing reaction, with some reports suggesting that epoxidation and deoxygenation could be a useful method of protecting valuable alkene functional groups.^[^ ^14^ ^]^ Epoxide deoxygenation has been reported using metals from across the periodic table including Au,^[^ ^15^ ^]^ In,^[^ ^16^ ^]^ Mo,^[^ ^17^ ^]^ Re,^[^ ^18^ ^]^ and Ti.^[^ ^19^ ^]^ This recent work from Harder and colleagues represents the first epoxide deoxygenation based on Mg, although Mg/MgX_2_ (X = halide) systems have been reported for the deoxygenation of β‐aryl‐α,β‐epoxy silanes to vinylsilanes.^[^ ^20^ ^]^ Mg‐based catalysts have also been reported for asymmetric epoxide ring‐opening and epoxide hydroboration.^[^ ^21^, ^22^, ^23^ ^]^

Our work was inspired by the precedence for magnesium reagents to deoxygenate epoxides to form metal‐oxo species, combined with the potential for magnesium‐oxo species to act as Brønsted bases. We wondered: can these concepts be combined to simultaneously deoxygenate then deprotonate and rearrange epoxides (Scheme 1E)? To answer this question, magnesium(I) dimers were reacted with a range of internal and external epoxides. Beyond epoxides, the reactivity of episulfides (thiiranes) was also explored, to compare and contrast the epoxide reactivity with these heavier sulfur‐based analogues.

## Results and Discussion

**Scheme 2:**
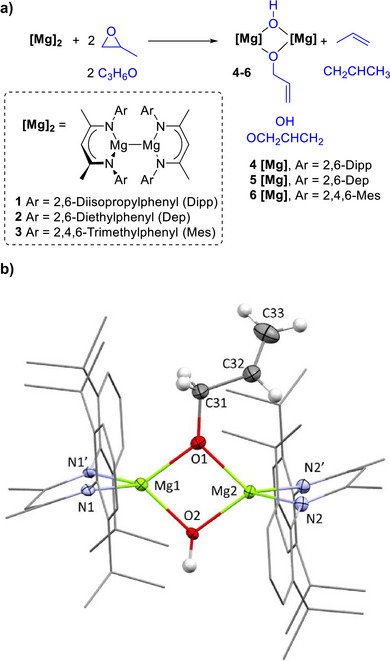
a) Reaction of magnesium(I) dimers **1**–**3** with propylene oxide to generate **4**–**6** (refer to ESI for details); b) molecular structure of dinuclear [^Dipp^NacnacMg(OH)(OCH_2_CHCH_2_)Mg^Dipp^Nacnac] **4**, with crystals obtained from C_6_D_6_ overnight at room temperature. Thermal ellipsoids shown at 50% probability, hydrogen atoms shown at a fixed radius. Selected parts of the ligand framework shown as wireframe. Most C‐bound hydrogen atoms of the ligand system and solvent have been omitted for clarity. The molecular structure has crystallographic mirror symmetry imposed by the space group. The [MgO]_2_ core, plus C31–C32, lie on the mirror plane and C33 is disordered over it by rotation of the C31─C32 bond. Symmetry related atoms are labeled with a prime. The THF solvate (Figure S97) has no imposed symmetry and crystals were obtained after five days at room temperature in THF.

The classic 2,6‐diisopropylphenyl (Dipp)‐substituted magnesium(I) dimer [^Dipp^NacnacMg]_2_ (**1**), where Nacnac = HC(C(CH_3_)NAr)_2_, was prepared and reacted with PO (Scheme 2a). PO was selected as the simplest of the liquid epoxides for relative ease of handling. Two sets of colourless block‐shaped crystals were obtained from C_6_D_6_ and from *d*_8_‐THF; single crystal X‐ray analysis revealed both products to be dinuclear [^Dipp^NacnacMg(OH)(OCH_2_CHCH_2_)Mg^Dipp^Nacnac] (**4**, Scheme 2b, Figures S96 and S97), crystallised as different solvates. In both structures, two crystallographically independent Mg centers are bridged by an hydroxide unit and an unsaturated allyl alkoxide group, which features a terminal C═C double bond as evidenced by the short C(32)═C(33) bond length of 1.258(4) Å in the crystals deposited from C_6_D_6_ (Scheme 2b, Figure S96). Monitoring the reaction by ^1^H NMR spectroscopy in C_6_D_6_ solvent showed that conversion of **1** to **4** was essentially quantitative. The elimination of propene was confirmed by diagnostic ^1^H and ^13^C NMR resonances, and further supported by cross‐peaks in the COSY and HSQC experiments (refer to ESI Figures S1–S5 for details).^[^ ^24^ ^]^ A distinctive ^1^H NMR resonance at ‐0.38 ppm was attributed to the Mg–OH, which was assigned by comparison to the previously reported dihydroxide magnesium dimer [^Dipp^NacnacMgOH]_2_ (**7**, Mg–OH *δ* = –0.45 ppm in C_6_D_6_),^[^ ^7^ ^]^ the relative integration of [1H] compared to the ligand resonances, and the absence of a cross‐peak in the HSQC spectrum confirming that this proton resides on a heteroatom. Additionally, DOSY NMR spectroscopy showed that all of the peaks of **4** have the same diffusion coefficient (Figure S6), indicating that the solid‐state structure of dinuclear **4** is maintained in the solution‐state.

It is well‐established that the steric bulk of the N‐substituents of Nacnac ligands influences the reactivity of Mg(I) dimers, with less bulky substituents leading to higher activities.^[^ ^25^ ^]^ Therefore, the less crowded analogues of Dipp‐substituted [^Dipp^NacnacMg]_2_ **1**, namely 2,6‐diethylphenyl (Dep)‐substituted [^Dep^NacnacMg]_2_ (**2**) and 2,4,6‐trimethylphenyl or mesityl (Mes)‐substituted [^Mes^NacnacMg]_2_ (**3**), were investigated to understand if the product formation was influenced by the steric bulk. The reaction with [^Dep^NacnacMg]_2_ **2** yielded colourless prism‐shaped crystals from toluene, and XRD analysis confirmed the formation of a dinuclear Mg complex [^Dep^NacnacMg(OH)(OCH_2_CHCH_2_)Mg^Dep^Nacnac] **5**. Analogous to **1**, **5** also contains a [MgO]_2_ core featuring one hydroxide unit and one allyl alkoxide group (refer to Figure S99 for structural details). For both Dep‐substituted **2** and Mes‐substituted **3**, NMR spectroscopy confirmed the clean and complete conversion to **5** and **6**, respectively (Scheme 2a, Figures S20–S29). Specifically, each ^1^H NMR spectra featured a diagnostic Mg–OH resonance (Mg–OH *δ* = –‐0.38 ppm for **4**; *δ* = –0.44 for **5**; *δ* = –0.41 ppm for **6**, all in C_6_D_6_), along with propene resonances. Overall, the X‐ray and spectroscopic data indicates the ubiquity of this reaction type across multiple Nacnac‐ligated Mg(I) dimers.

**Scheme 3:**
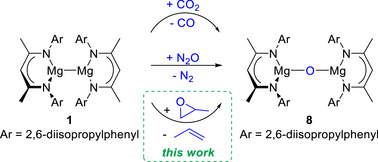
Deoxygenation of CO_2_ and N_2_O (literature) as well as propylene oxide (this work) to generate oxo‐bridged [(^Dipp^NacnacMg)_2_O] **8**. Note that with CO_2_, oxo‐bridged **8** is formed as a reaction intermediate.^[^ ^6^, ^7^ ^]^

While at first glance, the formation of the hydroxide group may be attributed to adventitious water, closer consideration shows that the reaction is stoichiometrically balanced (Scheme 2a). The presence of propene indicates that one equivalent of PO has been deoxygenated. Magnesium(I) dimers are known to abstract oxygen atoms from a variety of small molecules, such as CO_2_ and N_2_O, to produce oxo‐bridged [(^Dipp^NacnacMg)_2_O] (**8**, Scheme 3).^[^ ^6^, ^7^ ^]^ Here, we propose that the reaction occurs via a similar mechanism to that suggested by Harder (Scheme 1D), with reductive C–O bond cleavage to generate a Mg–C–C–O–Mg intermediate followed by alkene elimination to generate oxo‐bridged **8**.^[^ ^13^ ^]^

**Scheme 4:**
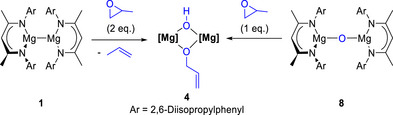
Synthesis of heteroleptic [^Dipp^NacnacMg(OH)(OCH_2_CHCH_2_)Mg^Dipp^Nacnac] **4** via reaction of Mg(I) dimer [^Dipp^NacnacMg]_2_ **1** with 2 (or 4) equivalents of PO (left), or reaction of oxo‐bridged [(^Dipp^NacnacMg)_2_O] **8** with 1 equivalent of PO (right).

To investigate whether [(^Dipp^NacnacMg)_2_O] **8** is a plausible reaction intermediate in the formation of heteroleptic [^Dipp^NacnacMg(OH)(OCH_2_CHCH_2_)Mg^Dipp^Nacnac] **4**, oxo‐bridged **8** was synthesised according to literature procedures.^[^ ^7^ ^]^ Subsequent reaction of [(^Dipp^NacnacMg)_2_O] **8** with 1 equivalent of PO in C_6_D_6_ gave heteroleptic **4**, as confirmed by NMR spectroscopy. Identical ^1^H NMR resonances were observed when Mg(I) dimer **1** was reacted with 2 equivalents of PO, and when oxo‐bridged **8** was reacted with 1 equivalent of PO under identical reaction conditions (Scheme 4). The only difference was the notable absence of propene ^1^H NMR resonances when starting with oxo‐bridged **8** (Scheme 4, right), which were present when starting from Mg(I) dimer **1** (Scheme 4, left, see Figure S76 for additional details). These observations support the initial deoxygenation of PO by [^Dipp^NacnacMg]_2_ **1**, to form oxo‐bridged **8** and propene, followed by deprotonation and rearrangement of the second equivalent of PO to generate heteroleptic **4**.

Oxo‐bridged magnesium species are highly Brønsted basic, and so, we wanted to further investigate whether the Mg–OH unit of **4** was generated from the deprotonation of PO rather than from adventitious water. An excess of deuterated propylene oxide (*d*_6_‐PO) (4 equivalents) was therefore added to [^Dipp^NacnacMg]_2_ **1** in C_6_D_6_ solvent in a J Young's NMR tube. An immediate colour change from yellow to colourless occurred upon addition and mixing of either PO or *d*_6_‐PO to Mg(I) dimer **1**, and ^1^H NMR spectroscopy showed that both reactions were complete by the time the reaction was analysed, even when the reaction was analysed immediately. With *d*_6_‐PO, the resultant ^1^H NMR spectrum showed identical ^Dipp^Nacnac ligand resonances to dinuclear [^Dipp^NacnacMg(OH)(OCH_2_CHCH_2_)Mg^Dipp^Nacnac] **4**, but with a notable absence of the ^1^H NMR resonances for the bridging OH and allyl alkoxide groups as well as propene (Figures S13 and S75). Providing further evidence for the formation of the deuterated analogue of **4**, [^Dipp^NacnacMg(OD)(OCD_2_CDCD_2_)Mg^Dipp^Nacnac] **4D**, ^2^D NMR analysis in C_6_D_6_ revealed a key resonance corresponding to the bridging Mg‐O*D* unit (*δ* = –0.38 ppm), as well as resonances for OCD_2_CDCD_2_ and *d*_6_‐propene (Figures S14 and S15). Crystals of **4D** suitable for X‐ray diffraction were obtained from C_6_D_6_ solvent at room temperature, with the same structural motif as the proteo‐analogue [^Dipp^NacnacMg(OH)(OCH_2_CHCH_2_)Mg^Dipp^Nacnac] **4**. The formation of a terminal alkene unit was evidenced by the short C(32)─C(33) bond length of 1.239(7) Å (Figure S98). Taken together, these observations provide evidence that oxo‐bridged **8** ultimately deprotonates a second equivalent of PO to produce [^Dipp^NacnacMg(OH)(OCH_2_CHCH_2_)Mg^Dipp^Nacnac] **4**.

**Scheme 5:**
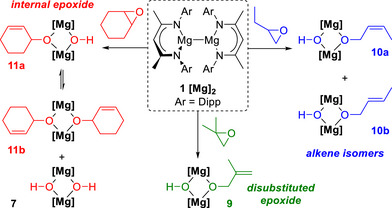
Reactivity of magnesium(I) dimer **1** with isobutylene oxide (center), butylene oxide (right) and cyclohexene oxide (left).

The epoxide substrate scope was subsequently expanded, using [^Dipp^NacnacMg]_2_ **1** as the Mg(I) precursor (Scheme 5). First, isobutylene oxide was selected as the more sterically bulky C4 analogue of propylene oxide (Scheme 5, center). The reaction proceeded in an analogous manner, generating dinuclear **9** with the concomitant elimination of isobutylene, as identified by multinuclear NMR spectroscopy and single crystal X‐ray diffraction (refer to ESI Figures S34–S39 and S100 for details). The linear analogue of isobutylene oxide, butylene oxide, was subsequently tested to determine whether there was a stereochemical preference for the alkene unit generated to adopt a *cis*‐ or a *trans*‐ geometry (Scheme 5, right). Examination of the ^1^H NMR spectra showed the elimination of 1‐butene and the presence of both the *cis*‐ (**10a**) and *trans*‐ (**10b**) alkoxide species in a relative ratio of 3:5 (refer to ESI Figures S40–S44). These were assigned on the basis of the ^1^H NMR spectra, with the relative ratio determined by the integrals of the two distinctive Nacnac‐CH protons at 4.80 and 4.82 ppm. This ratio shows that while a stereochemical preference exists, it is somewhat slight.

Some reports indicate that epoxide deoxygenation is more facile with terminal epoxides than with internal epoxides.^[^ ^26^ ^]^ To probe whether [^Dipp^NacnacMg]_2_ **1** could deoxygenate internal epoxides, the pseudo infinite alkyl chain of cyclohexene oxide (CHO) was thus investigated (Scheme 5, left). Monitoring the reaction by ^1^H NMR spectroscopy in C_6_D_6_ showed the elimination of cyclohexene, as well as the formation of diagnostic resonances attributed to the bridging alkoxide/hydroxide complex **11a**. Specifically, the Mg–OH resonance was observed at –0.32 ppm, in a 1:1 ratio with the resonances from the allyl alkoxide unit and a 2:1 ratio with the ligand:OH resonances. Notably, the Mg–OH resonance is shifted from that of the dihydroxide magnesium dimer [^Dipp^NacnacMgOH]_2_ **7** (*δ* = –0.45 ppm in C_6_D_6_). However, crystallisation from C_6_D_6_ yielded the symmetric complex **11b**,^[^ ^27^ ^]^ which is presumed to form due to disproportionation, along with [^Dipp^NacnacMgOH]_2_ **7** (Scheme 5, left). Analysing the ^1^H NMR spectrum of **11a** in C_6_D_6_ after 3 days at 80 °C showed the complete disappearance of the ^1^H NMR resonances of **11a** (Figure S86), and the formation of dihydroxide magnesium dimer [^Dipp^NacnacMgOH]_2_ (**7**). This indicates that ligand redistribution occurs over time with **11a**. This may be due to the increased steric bulk and/or the enhanced solubility of the cyclohexene oxide bridge compared to the products from the terminal epoxides (**4–6**, **9**), which all crystallised as the heteroleptic hydroxide/alkoxide dinuclear magnesium complexes. Heating complexes **4**–**6**, **9** and **10a**/**b** at 80 °C for 2 days showed that disproportionation occurred in all cases, as evidenced by an increase in the corresponding Mg–OH peak of the dihydroxide magnesium dimer, [^Ar^NacnacMgOH]_2_ (Ar = 2,6‐diisopropylphenyl, 2,6‐diethylphenyl and 2,4,6‐trimethylphenyl), along with some degradation (see Figures S81–85).

The differences in the chemical shifts of these OH resonances in **4** and **9**–**11a** are subtle but reproducible, and different to that of the dihydroxide magnesium dimer [^Dipp^NacnacMgOH]_2_ **7** (Figure S80), suggesting that the dinuclear structures persist in C_6_D_6_ solution. This is further supported by the observation of a single diffusion coefficient for the μ_2_‐OH and μ_2_‐alkoxide resonances in the DOSY spectra (refer to ESI). Complexes **4**–**6** and **9**–**11a** can be viewed as molecular magnesium‐hydroxide sources, overcoming the solubility limitations generally associated with magnesium hydroxide. In contrast, after reacting [^Dipp^NacnacMg]_2_ **1** with PO, butylene oxide or isobutylene oxide, followed by solvent removal in vacuo, the attempted redissolution of the product in C_6_D_6_ only partially solubilised the reaction product. In all cases, ^1^H NMR analysis showed an increased formation of the dihydroxide dimer [^Dipp^NacnacMgOH]_2_ **7** along with heteroleptic products **4**, **9,** and **10**, suggesting that the insoluble component contains the bis‐alkoxide species [^Dipp^NacnacMg(OR)]_2_.

**Scheme 6:**
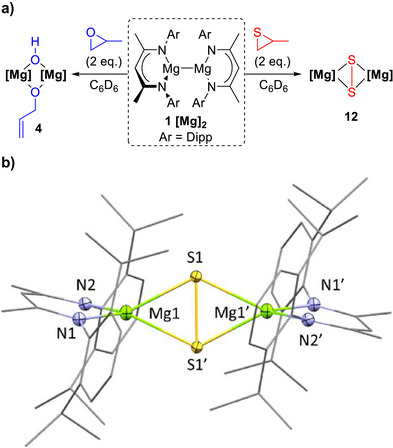
a) Deoxygenation of epoxides (left) versus desulfonation of thiiranes (right) by [^Dipp^NacnacMg]_2_ **1** with a 2:1 ratio in C_6_D_6_ solvent; b) molecular structure of disulfide‐bridged **12**, crystals obtained from a toluene/hexane solvent mixture stored at –30 °C overnight. Thermal ellipsoids shown at 50% probability for heteroatoms. Hydrogen atoms of the ligand system and hexane solvent have been omitted for clarity.

To gain insights into the reactivity differences between epoxides and their heavier sulfur analogues, thiiranes,^[^ ^28^ ^]^ Mg(I) dimer [^Dipp^NacnacMg]_2_ **1** was reacted with 2 equivalents of propylene sulfide (PS) in C_6_D_6_ solvent. ^1^H NMR analysis showed the formation of propene along with a new product, which was crystallized from a toluene/hexane mixed solvent system at ‐30 °C. Single crystal X‐ray analysis revealed this product to be the disulfide‐bridged species [(^Dipp^NacnacMg)S]_2_ (**12**, Scheme 6, right, see Figures S101–S103 for additional structural analysis). It is worth noting that while disulfide‐bridged Mg species have been reported previously, these have generally been formed via reactions with elemental sulfur (S_8_) rather than the desulfurisation of thiiranes.^[^ ^13^, ^29^ ^]^

While [^Dipp^NacnacMg]_2_ **1** deoxygenates PO and desulfurises PS, the two products are noticeably different. Reaction with PO generates the heteroleptic hydroxide/allyl alkoxide species **4**, whereas PS generates disulfide‐bridged [(^Dipp^NacnacMg)S]_2_ **12**. This variance arises from key differences between oxygen and sulfur. First, the p*K*_a_ values of alcohols are much higher those of thiols, with respective p*K*_a_ values of around 16 versus 8.^[^ ^28^ ^]^ It, therefore, stands to reason that oxo‐bridged [(^Dipp^NacnacMg)_2_O] **8** acts as a relatively strong Brønsted base, deprotonating PO or *d*_6_‐PO to generate a Mg–OH or Mg–OD unit, respectively (vide supra, Figure S75), in line with recent reports from the Stasch group (Scheme 1a).^[^ ^4^ ^]^ It is also worth noting that PO is an ether with a β‐methyl group. Multiple studies have shown that Brønsted basic alkyl lithium reagents selectively deprotonate β‐methyl‐substituted ethers at the primary β‐CH bonds, including Et_2_O, 2‐methyl‐tetrahydrofuran (2‐Me‐THF) and 2‐methyl‐tetrahydropyran (2‐Me‐THP).^[^ ^8^, ^30^ ^]^ In the case of the cyclic ethers 2‐Me‐THF and 2‐Me‐THP, subsequent ring‐opening generates lithium alkoxides with terminal alkene units, providing strong literature precedence for the formation of **4** via a deprotonative pathway.^[^ ^8^, ^31^, ^32^, ^33^ ^]^

The formation of disulfide bonds in **12** is perhaps unsurprising, as S─S bonds are prevalent in nature. Indeed, the thermal decomposition of thiirane has been suggested to occur via sulfur‐transfer reactions, where one thiirane abstracts the S atom from a second thiirane ring, to form a thiirane‐1‐sulfide (C_2_H_4_S═S) species along with ethene as a by‐product.^[^ ^34^, ^35^ ^]^ The equivalent decomposition pathways have not been reported for epoxides, which is likely because S─S bonds are approximately twice as strong as the analogous peroxide bonds.^[^ ^36^ ^]^

**Scheme 7:**
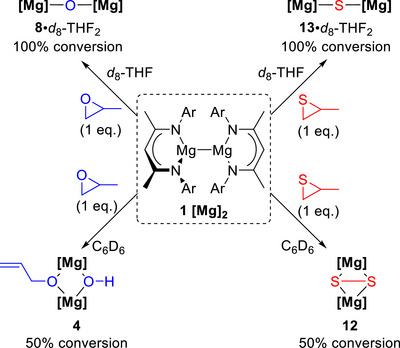
Solvent dependence of the reaction of [^Dipp^NacnacMg]_2_ **1** with 1 equivalent of propylene oxide or propylene sulfide in *d*_8_‐THF (top) or C_6_D_6_ (bottom).

To examine the formation of disulfide‐bridged [(^Dipp^NacnacMg)S]_2_ **12**, Mg(I) dimer [^Dipp^NacnacMg]_2_ **1** was combined with PS, this time in a 1:1 ratio in C_6_D_6_ (Scheme 7, right). ^1^H NMR analysis showed 50% unreacted **1**, 50% conversion to the disulfide‐bridged [(^Dipp^NacnacMg)S]_2_ **12** and the formation of propene (refer to ESI, Figure S92). Upon changing the solvent from C_6_D_6_ to *d*_8_‐THF, addition of 1 equivalent of PS gave a new set of ligand resonances along with diagnostic ^1^H NMR resonances for propene (Scheme 7, Figures S57–S62). This indicates that the reaction initially follows the same pathway as the epoxide analogue, with the formation of sulfido‐bridged [(^Dipp^NacnacMg)_2_S·THF_2_] (**13**·*d*_8_‐THF_2_, S99) instead of oxo‐bridged [(^Dipp^NacnacMg)_2_O] **8** (refer to ESI, Figure S79 for details). There is literature precedent for sulfido‐bridged complexes to act as sulfur transfer agents, and thus, **8** and **13** may have potential as oxygen or sulfur transfer agents, respectively.^[^ ^37^, ^38^ ^]^ Stasch and co‐workers have previously reported sulfido‐bridged [(NacnacMg)_2_S] structures for several Dipp‐N substituted Nacnac ligands with *^i^* Pr groups at the 2‐ and 4‐ positions.^[^ ^39^ ^]^ These complexes include Lewis acid/base adducts with ketone, NHC, and isonitrile donors, which were formed using Ph_3_P═S as the sulfur source.

The different products obtained in *d*_8_‐THF and C_6_D_6_ upon reacting [^Dipp^NacnacMg]_2_ **1** with 1 equivalent of PS correlate with the analogous PO reactions (Scheme 7). The reaction between Mg(I) dimer **1** and 1 equivalent of PO in *d*_8_‐THF generated oxo‐bridged [(^Dipp^NacnacMg)_2_O·*d*_8_‐THF_2_] (**8**·*d*_8_‐THF_2_, Scheme 7 top left). This was confirmed by the identical ^1^H NMR spectra in *d*_8_‐THF obtained for **8**·*d*_8_‐THF_2_ whether it was prepared via the reaction of **1** with PO or with N_2_O (Figure S78). Notably, the PO route reported herein provides a relatively straightforward method of generating oxo‐bridged **8**·*d*_8_‐THF_2_ without the need for a gaseous reagent such as N_2_O.^[^ ^6^, ^7^ ^]^ A small quantity of heteroleptic **4** was also observed; this shows that the conversion of [^Dipp^NacnacMg]_2_ **1** to heteroleptic **4** still occurs in *d*_8_‐THF solvent, yet the deprotonation/rearrangement of the second epoxide is relatively slow compared to the deoxygenation of the first equivalent. In contrast, combining [^Dipp^NacnacMg]_2_ **1** with 1 equivalent of PO in C_6_D_6_ gave 50% conversion to heteroleptic **4**, along with 50% of unreacted **1**; subsequent addition of a second equivalent of PO gave complete formation of **4** (Figure S91). This indicates that deprotonation/rearrangement of the second equivalent of epoxide is far more rapid than epoxide deoxygenation in C_6_D_6_ solvent, providing a contrast with the reactivity in *d*_8_‐THF.

The reactivity of Mg(I) dimers is known to differ in different solvents. The presence of one equivalent of a Lewis base, such as DMAP or NHC, can activate and enhance the reactivity of Mg─Mg bonds.^[^ ^40^, ^41^ ^]^ The reactivity difference observed with 1 equivalent of PO in C_6_D_6_ versus *d*_8_‐THF solvent can thus be attributed to the latter acting as a labile Lewis donor.^[^ ^42^ ^]^ In C_6_D_6_ solvent, the Lewis basic epoxide would be expected to activate the Mg─Mg bond towards epoxide deoxygenation, due to polarisation and elongation of the Mg─Mg bond. The newly formed Mg–O–Mg unit can then undergo rapid deprotonation and rearrangement of a second equivalent of epoxide to generate **4**. In *d*_8_‐THF solvent, the excess of Lewis basic *d*_8_‐THF competes with epoxide coordination, disfavouring the rapid deprotonation and rearrangement of a second epoxide and instead preferentially forming oxo‐bridged **8**·*d*_8_‐THF_2_. Indeed, ^1^H NMR monitoring of **1** with two equivalents of PO in *d*_8_‐THF shows the initial formation of **8**, followed by subsequent formation of **4** over a period of one day (See Figures S94 and S95).

The products obtained for the thiirane reactions indicate a similar solvent dependance (Scheme 7, right). ^1^H NMR analysis showed that combining 1 equivalent of PS with **1** in C_6_D_6_ generated disulfide‐bridged **12** along with propene and unreacted **1** (Scheme 7, bottom right). Whether added sequentially or simultaneously, the addition of 2 equivalents of PS to **1** in C_6_D_6_ gave complete conversion to disulfide **12** (Scheme 7, top right, Figures S87 and S92). In contrast, combining 2 equivalents of PS with **1** in THF gave only monosulfide bridged **13**·THF_2_, even after heating the reaction at 50 ⁰C for 1 day. This suggests that mono‐sulfide **13** is an intermediate in the formation of disulfide‐bridged **12**, and that an excess of Lewis basic THF outcompetes PS coordination preventing subsequent reactivity.

Using Et_2_O solvent, as a weaker Lewis base than THF, addition of 2 equivalents of PS deposited a set of crystals overnight that contained a disordered mixture of monosulfide **12**·(Et_2_O)_2_ (84%) and disulfide‐bridged **13**·(Et_2_O)_2_ (16%) (Figures S104 and S105). These observations indicated that monosulfide **13**·(Et_2_O)_2_ is an intermediate in the synthesis of disulfide‐bridged **12**·(Et_2_O)_2_. Accordingly, 2 equivalents of PS were added to the ether adduct, **13**·(Et_2_O)_2_, in C_6_D_6_ solvent, which gave disulfide **12**·(Et_2_O)_2_ along with propene (Figure S93). The ether could be subequently removed in vacuo to give adduct free **12**.

**Scheme 8:**
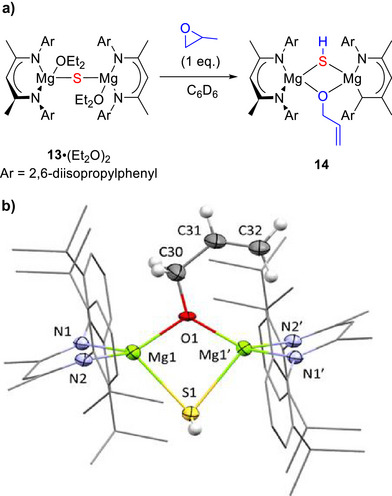
a) Reaction of sulfide‐bridged **13**·(Et_2_O)_2_ with 1 equivalent of propylene oxide in C_6_D_6_. b) Molecular structure of heterochalcogen bridged **14**. Thermal ellipsoids shown at 50% probability for heteroatoms. C‐bound hydrogen atoms of the ligand system have been omitted for clarity.

Adding instead 1 equivalent of PO into sulfide‐bridged **13**·(Et_2_O)_2_ generated the heteroleptic, mixed‐chalcogen species **14** (Scheme 8, Figure S107). This is the first mixed alkoxide/bisulfide bridged Mg complex and demonstrates that with electrophilic epoxide substrates, Mg─S bonds are capable of acting as Brønsted bases. Overall, these observations reveal a key trend; noncoordinating solvents favour the immediate reaction of Mg(I) dimer **1** with 2 equivalents of PO or PS, even when a 1:1 stoichiometry is employed, whereas more strongly coordinating solvents preferentially react with 1 equivalent to form the bridging oxo‐ or sulfide‐species. Insertion of the second equivalent of PO or PS is thus decelerated by coordinating solvents, and isolation of the sulfide‐bridged species enables the formation of heteroleptic mixed‐chalcogen species.

## Conclusion

Overall, these studies show that Mg(I) dimer [^Dipp^NacnacMg]_2_ **1** can deoxygenate epoxides to form an oxo‐bridged magnesium species [^Dipp^NacnacMg‐O‐Mg^Dipp^Nacnac] **8**, which can subsequently deprotonate and rearrange a second epoxide to form heteroleptic [^Dipp^NacnacMg(OH)(OCH_2_CHCH_2_)Mg^Dipp^Nacnac] **4**. The reaction is ubiquitous with different Mg(I) precursors, specifically [^Dep^NacnacMg]_2_ **2** and [^Mes^NacnacMg]_2_ **3**, as well as several epoxides including propylene oxide, butylene oxide, isobutylene oxide and cyclohexene oxide. In contrast, reaction of [^Dipp^NacnacMg]_2_ **1** with two equivalents of propylene sulfide generates a disulfide bridged species. This reflects both the lower Brønsted basicity of thiols compared to alkoxides, and the propensity of thiiranes to form S─S bonds. These studies demonstrate that metal‐hydroxides, which are often dismissed as decomposition products in organometallic chemistry, can be formed by metal oxo‐bridged species acting as potent Brønsted bases. Overall, these initial studies indicate that there is a potentially unrecognised and untapped potential to exploit the Brønsted basicity of bridging metal‐oxo species in a range of chemical transformations, with applications in both stoichiometric and catalytic processes.

## Supporting Information

The authors have cited additional references within the Supporting Information.^[^ ^7^, ^13^, ^27^, ^29^, ^34^, ^35^, ^39^, ^42^, ^44^, ^45^, ^46^, ^47^, ^48^, ^49^, ^50^, ^51^, ^52^, ^53^ ^]^

## Conflict of Interests

The authors declare no conflict of interest.

## Supporting information



Supporting Information

Supporting Information

## Data Availability

The data that support the findings of this study are available in the Supporting Information of this article.
