# Supplementary material for: Reactivity of Low Valent Magnesium(I) Complexes with Epoxides and Episulfides
Source: Angew Chem Int Ed Engl. 2025 Nov 2;64(52):e20259. doi: 10.1002/anie.202520259 (PMC12723457; doi:10.1002/anie.202520259)
Supplement: Supplementary file 1 — Supporting Information [file ANIE-64-e20259-s001.docx]

Reactivity of Low Valent Magnesium(I) Complexes with Epoxides and Episulfides

**Electronic Supporting Information**

Peter J. Shaw, Gary S. Nichol and Jennifer A. Garden*

School of Chemistry, University of Edinburgh, Edinburgh, EH9 3FJ, United Kingdom
E-mail: j.garden@ed.ac.uk

**Table of Contents**

[**General Experimental Details** 3](#_Toc210905642)

[**Complex Synthesis and Characterisation** 4](#_Toc210905643)

[**Synthesis of [^Dipp^NacnacMg(OH)(OCH_2_CHCH_2_)Mg^Dipp^Nacnac] (4)** 4](#_Toc210905644)

[**Synthesis of [^Dipp^NacnacMg(OD)(OCD_2_CDCD_2_)Mg^Dipp^Nacnac] (4D)** 12](#_Toc210905645)

[**Synthesis of [^Dep^NacnacMg(OH)(OCH_2_CHCH_2_)Mg^Dep^Nacnac] (5)** 16](#_Toc210905646)

[**Synthesis of [^Mes^NacnacMg(OH)(OCH_2_CHCH_2_)Mg^Mes^Nacnac] (6)** 19](#_Toc210905647)

[**Literature synthesis of [(^Dipp^NacnacMg)_2_O] (8)** 22](#_Toc210905648)

[**Alternative NMR scale synthesis of [(^Dipp^NacnacMg)_2_O**•**THF_2_] (8**•**THF_2_)** 24](#_Toc210905649)

[**Synthesis of [^Dipp^NacnacMg(OH)(OCH_2_C(CH_2_)(CH_3_))Mg^Dipp^Nacnac] (9)** 26](#_Toc210905650)

[**Synthesis of [^Dipp^NacnacMg(OH)(OCH_2_CH=CHCH_2_)Mg^Dipp^Nacnac] (10a(-*cis*)/10b(-*trans*))** 30](#_Toc210905651)

[**Synthesis of [^Dipp^NacnacMg(OH)(OCH_2_CHCHCH_2_CH_2_CH_2_)Mg^Dipp^Nacnac] (11a)** 34](#_Toc210905652)

[**Synthesis of [^Dipp^NacnacMgS]_2_ (12) in various solvents** 38](#_Toc210905653)

[**Synthesis of [(^Dipp^NacnacMg)_2_S**•**THF_2_] (13**•**THF_2_)** 42](#_Toc210905654)

[**Synthesis of [(^Dipp^NacnacMg)_2_S**•**(OEt_2_)_2_] (13**•**(Et_2_O)_2_)** 46](#_Toc210905655)

[**Synthesis of [^Dipp^NacnacMg(SH)(OCH_2_CHCH_2_)Mg^Dipp^Nacnac] (14)** 50](#_Toc210905656)

[**NMR Spectroscopic Investigations** 54](#_Toc210905657)

[**Comparison of Complexes 4 and 4D** 54](#_Toc210905658)

[**Synthesis of Complex 4 from [(^Dipp^NacnacMg)_2_O] (8)** 55](#_Toc210905659)

[**Alternative Synthesis of [(^Dipp^NacnacMg)_2_O**•***d*_8_-THF_2_] (8**•***d*_8_-THF_2_)** 56](#_Toc210905660)

[**Comparison of [(^Dipp^NacnacMg)_2_O**•**THF_2_] (8**•**THF_2_) and [(^Dipp^NacnacMg)_2_S**•**THF_2_] (13**•**THF_2_) in *d*_8_-THF** 58](#_Toc210905661)

[**Comparison of Hydroxide Shifts in ^Dipp^Nacnac Magnesium Complexes 4 and 9-11** 59](#_Toc210905662)

[**Stability of Heteroleptic Complexes** 60](#_Toc210905663)

[**NMR Scale Synthesis of [^Dipp^NacnacMgS]_2_ (12) Showing Elimination of Propene** 64](#_Toc210905664)

[**NMR Scale Synthesis of [(^Dipp^NacnacMg)_2_S**•**THF_2_] (13**•**THF_2_) Showing Elimination of Propene** 65](#_Toc210905665)

[**Comparison of [^Dipp^NacnacMg]_2_ with PO (2 eq) and PS (2 eq)** 66](#_Toc210905666)

[**Reactivity of [^Dipp^NacnacMg]_2_ with PO in *d*_8_-THF** 67](#_Toc210905667)

[**Reactivity of [^Dipp^NacnacMg]_2_ with PO in C_6_D_6_** 68](#_Toc210905668)

[**Reactivity of [^Dipp^NacnacMg]_2_ with PS in C_6_D_6_** 69](#_Toc210905669)

[**Synthesis of Complex 12 from 13**•**(Et_2_O)_2_** 70](#_Toc210905670)

[**Proposed Mechanisms for the Reactivity of [^Dipp^NacnacMg]_2_ with Propylene Oxide** 71](#_Toc210905671)

[**Single Crystal X-Ray Diffraction Data** 73](#_Toc210905672)

[**References** 85](#_Toc210905673)

## **General Experimental Details**

All manipulations involving air or water sensitive compounds were performed either in a glovebox or using standard Schlenk techniques under an argon atmosphere. All reagents and solvents were obtained from Merck, Fisher Scientific, Fluorochem Ltd, Qmx Laboratories and used without further purification unless stated otherwise. Dry solvents (THF, diethyl ether, toluene and hexane) were collected from a solvent purification system (Innovative Technologies), dried over activated 4 Å molecular sieves and stored under argon. *d*_6_-benzene and *d*_8_-THF solvents for NMR spectroscopy studies were dried over CaH_2,_ degassed by three freeze−pump−thaw cycles, distilled under reduced pressure and stored over activated 4 Å molecular sieves under an argon atmosphere. Propylene oxide, cyclohexene oxide, isobutylene oxide, butylene oxide and propylene sulfide were dried over CaH_2,_ degassed by three freeze−pump−thaw cycles, distilled under reduced pressure and stored under an argon atmosphere. *d*_6_*-*Propylene oxide was supplied in an ampoule under vacuum and used without further purification. Ligands ^Dipp^NacnacH, ^Dep^NacnacH, ^Mes^NacnacH,^[44]^ magnesium iodide ether adducts [^Dipp^NacnacMgI.OEt_2_],^[45]^ [^Dep^NacnacMgI.OEt_2_],^[46]^  [^Mes^NacnacMgI.OEt_2_],^[45]^  dimers [^Dipp^NacnacMg]_2_ **1**, [^Dep^NacnacMg]_2_ **2**, [^Mes^NacnacMg]_2_ **3**,^[47]^ and oxo-bridged [(^Dipp^NacnacMg)_2_O] **8** ^[7]^ were prepared using adapted literature procedures. Propylene sulfide was synthesised according to literature.^[48]^

Diffusion-Ordered Spectroscopy (DOSY) NMR experiments were performed at 300 K on a Bruker Ascend 2 channel instrument operating at a frequency of 500 MHz for proton resonance under TopSpin (version 3.2, Bruker Biospin, Karlsruhe) and equipped with a z-gradient DCH/5mm tuneable “CryoProbe”^TM^ probe and a GRASP II gradient spectroscopy accessory providing a maximum gradient output of 53.5 G/cm (5.35G/cmA). Diffusion ordered NMR data was acquired using the Bruker pulse program dstebpgp3s with a spectral width of 10330 Hz (centred on 6.175 ppm) and 32768 data points. A relaxation delay of 2 s was employed along with a diffusion time (D) of 100 ms and a longitudinal eddy current delay (LED) of 5 ms. Bipolar gradients pulses (d/2) of 1.5 ms and homospoil gradient pulses of 0.6 ms were used. The gradient strengths of the 3 homospoil pulses were -13.17%, -17.13%, -15.37%. 16 experiments were collected with the bipolar gradient strength, initially at 5% (1^st^ experiment), linearly increased to 95% (16^th^ experiment). All gradient pulses were smooth-square shaped (SMSQ10.100) and after each application a recovery delay of 200 µs used. The experiment was run with 16 scans per increment, employing one stimulated echo with two spoiling gradients.

DOSY plots were generated by using the DOSY processing module of TopSpin. DOSY NMR spectra were formatted in TopSpin 4.3.0. Parameters were optimised empirically to find the best quality of data for presentation purposes. Diffusion coefficients were calculated by fitting intensity data to the Stejskal-Tanner expression.

Crystals for single-crystal X-ray diffraction experiments were first coated in Paratone oil, then a suitable sample was selected on a Mitigen tip and mounted on the diffractometer. Data for **4**, **4D**, **5**, **9**, **12**•**(Et_2_O)_2_**, **13**•**(Et_2_O)_2_**, **13**•**THF_2_** and **14**, was collected using either a Bruker D8 VENTURE diffractometer or Rigaku XtaLAB Synergy, HyPix-Arc 100 diffractometer, with Mo or Cu K-alpha radiation and a crystal temperature of 100 K. The structures were solved by direct or dual-space methods using **ShelXS**^[49]^  or **ShelXT 2018/2**.^[50]^ The model was refined with **ShelXL**^[51]^  or olex2.refine^[52]^  using full matrix least squares minimisation on ***F*^2^**. **Olex2 1.5-beta**^[53]^  was used as the graphical interface to both programs.

We note that the molecular structure of **11b** has been previously reported.^[27]^

## **Complex Synthesis and Characterisation**

### **Synthesis of [^Dipp^NacnacMg(OH)(OCH_2_CHCH_2_)Mg^Dipp^Nacnac] (4)**

**Method A (in C_6_D_6_):**

In a glovebox, [^Dipp^NacnacMg]_2_ (10.7 mg, 12.1 μmol, 1 eq) was dissolved in C_6_D_6_ (0.6 mL) and transferred to a J Youngs tap NMR tube followed by the addition of propylene oxide (2.8 mg, 48.3 μmol, 3.38 μL, 4 eq). Upon shaking the solution turned from yellow to colourless and the reaction mixture was analysed by NMR spectroscopy. The tube was left overnight at room temperature yielding clear, colourless crystals which were characterised by single crystal XRD analysis (Figure S96).

Complex **4**: ^1^H NMR (500 MHz, C_6_D_6,_ 300 K) δ 7.17 – 7.12 (m, 4H, Ar-*H*)*, 7.04 (d, *J* = 7.6 Hz, 8H, Ar-*H*), 6.08 (ddt, *J* = 16.6, 10.1, 6.0 Hz, 1H, OCH_2_C*H*CH_2_), 5.08 (dd, *J* = 16.6, 1.7 Hz, 1H, OCH_2_CHC*H*_2_), 5.05 – 5.00 (m, 1H, OCH_2_CHC*H*_2_)^#^, 4.81 (s, 2H, Nacnac-C*H*), 4.29 (dt, *J* = 6.0, 1.7 Hz, 2H, OC*H*_2_CHCH_2_), 3.15 (hept, *J* = 6.9 Hz, 8H, C*H*(CH_3_)_2_), 1.45 (s, 12H, Nacnac-C*H*_3_), 1.13 (d, *J* = 6.9 Hz, 24H, CH(C*H*_3_)_2_), 0.83 (d, *J* = 6.9 Hz, 24H, CH(C*H*_3_)_2_), -0.38 (s, 1H, O*H*).

^13^C NMR (126 MHz, C_6_D_6,_ 300 K) δ 169.3 (N*C*CH_3_), 147.2 (Ar-*C*), 142.6 (Ar-*C*), 142.1 (OCH_2_*C*HCH_2_), 125.5 (Ar-*C*), 124.0 (Ar-*C*), 113.7 (OCH_2_CH*C*H_2_), 95.3 (Nacnac-*C*H), 66.2 (O*C*H_2_CHCH_2_), 28.3 (*C*H_2_(CH_3_)_2_), 24.8 (Nacnac-*C*H_3_), 24.3 (CH_2_(*C*H_3_)_2_), 24.2 (CH_2_(*C*H_3_)_2_).

*Overlap with C_6_D_6_
^#^Overlap with propene


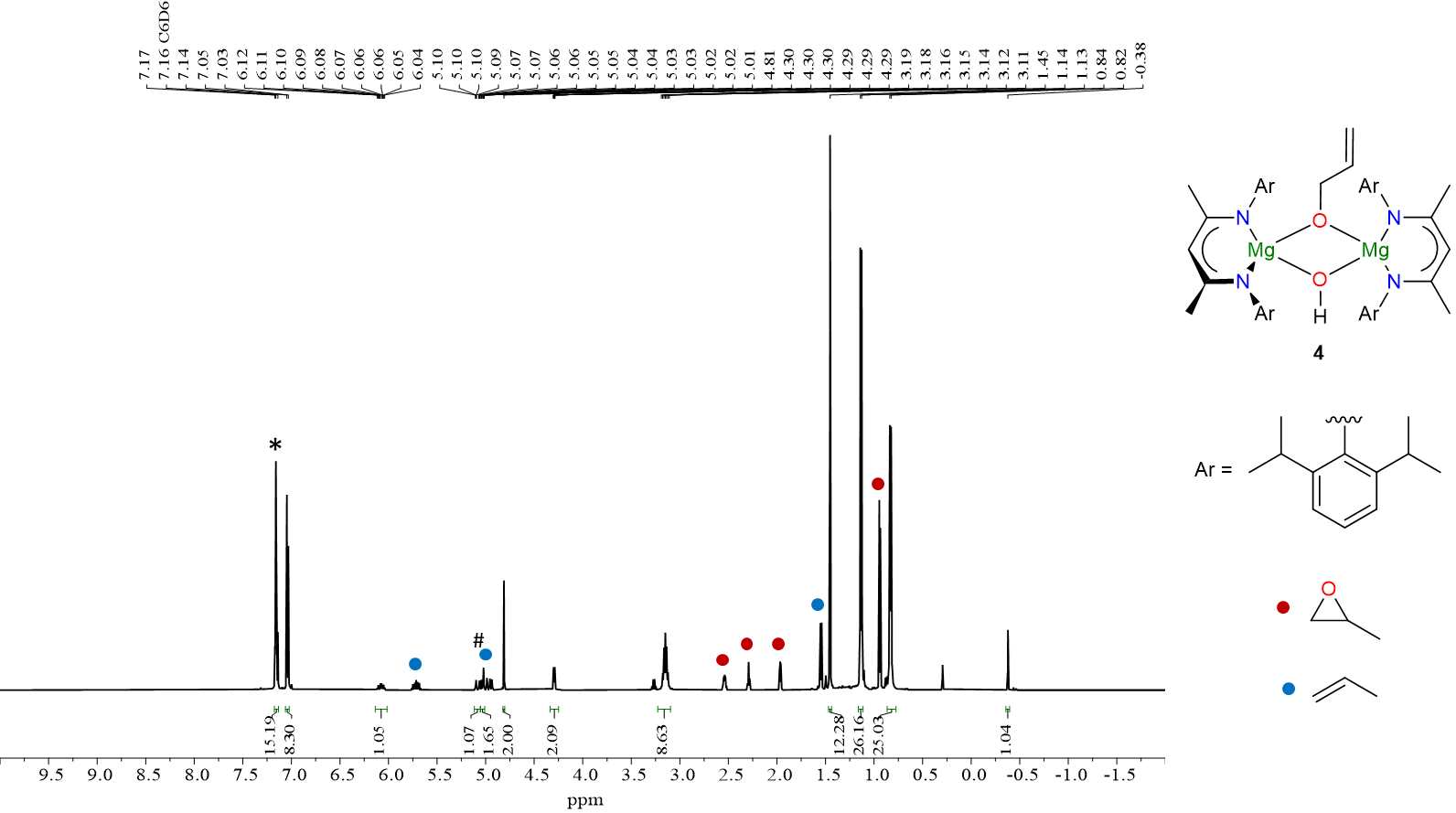


**Figure S1:** ^1^H NMR spectrum of **4** in C_6_D_6_ recorded at 500 MHz, 300 K. Propene and propylene oxide resonances are identified. *Overlapping peak with C_6_D_6_. ^#^Overlapping peak with propene.


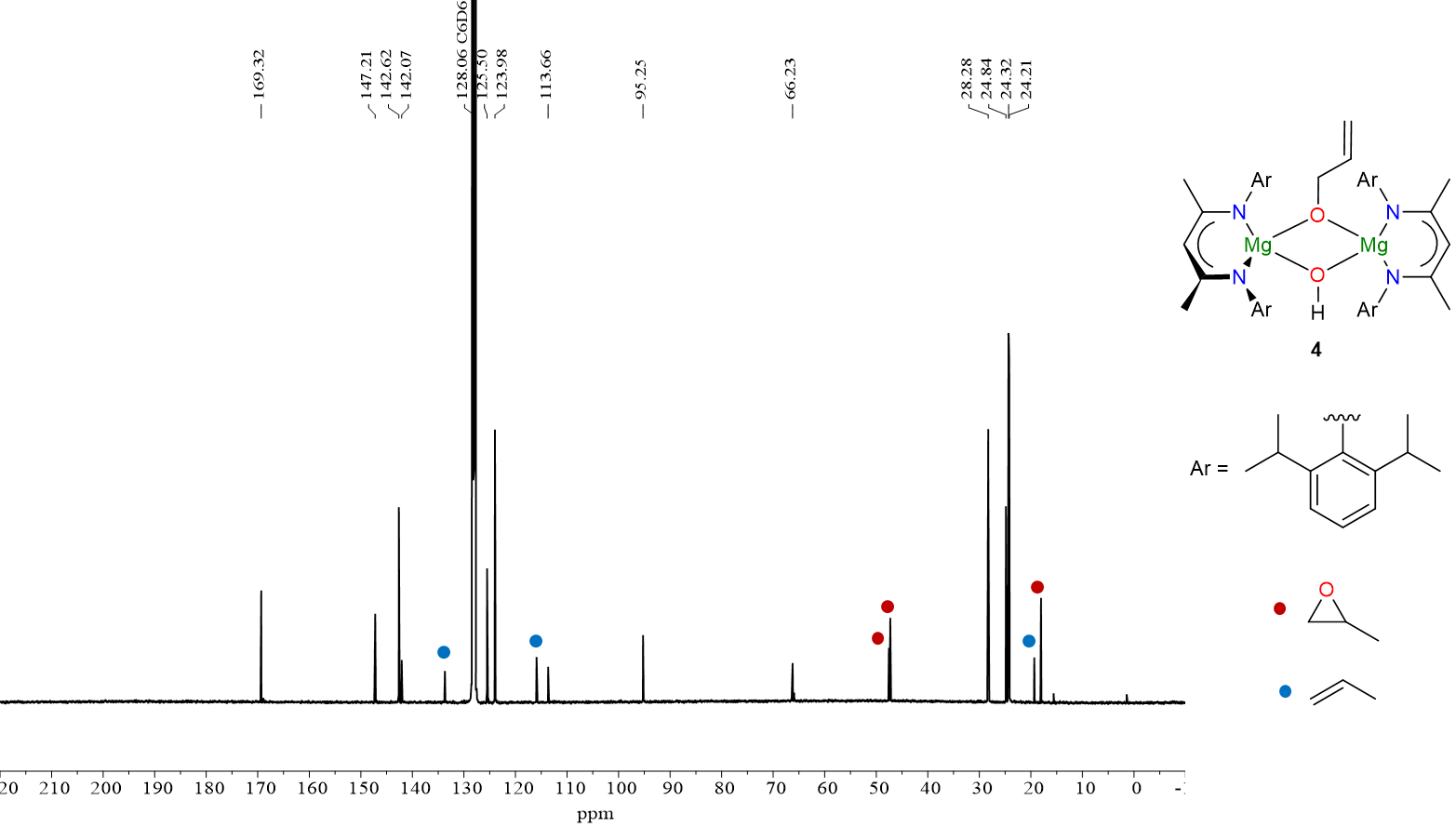


**Figure S2:** ^13^C{^1^H} NMR spectrum of **4** in C_6_D_6_ recorded at 126 MHz, 300 K. Propene and propylene oxide resonances are identified.

**Figure S3:** ^1^H-^1^H COSY NMR spectrum of **4** with propene and propylene oxide in C_6_D_6_.

**Figure S4:** ^1^H-^13^C HSQC NMR spectrum of **4** with propene and propylene oxide in C_6_D_6_.

**Figure S5:** ^1^H-^13^C HMBC NMR spectrum of **4** with propene and propylene oxide in C_6_D_6_.


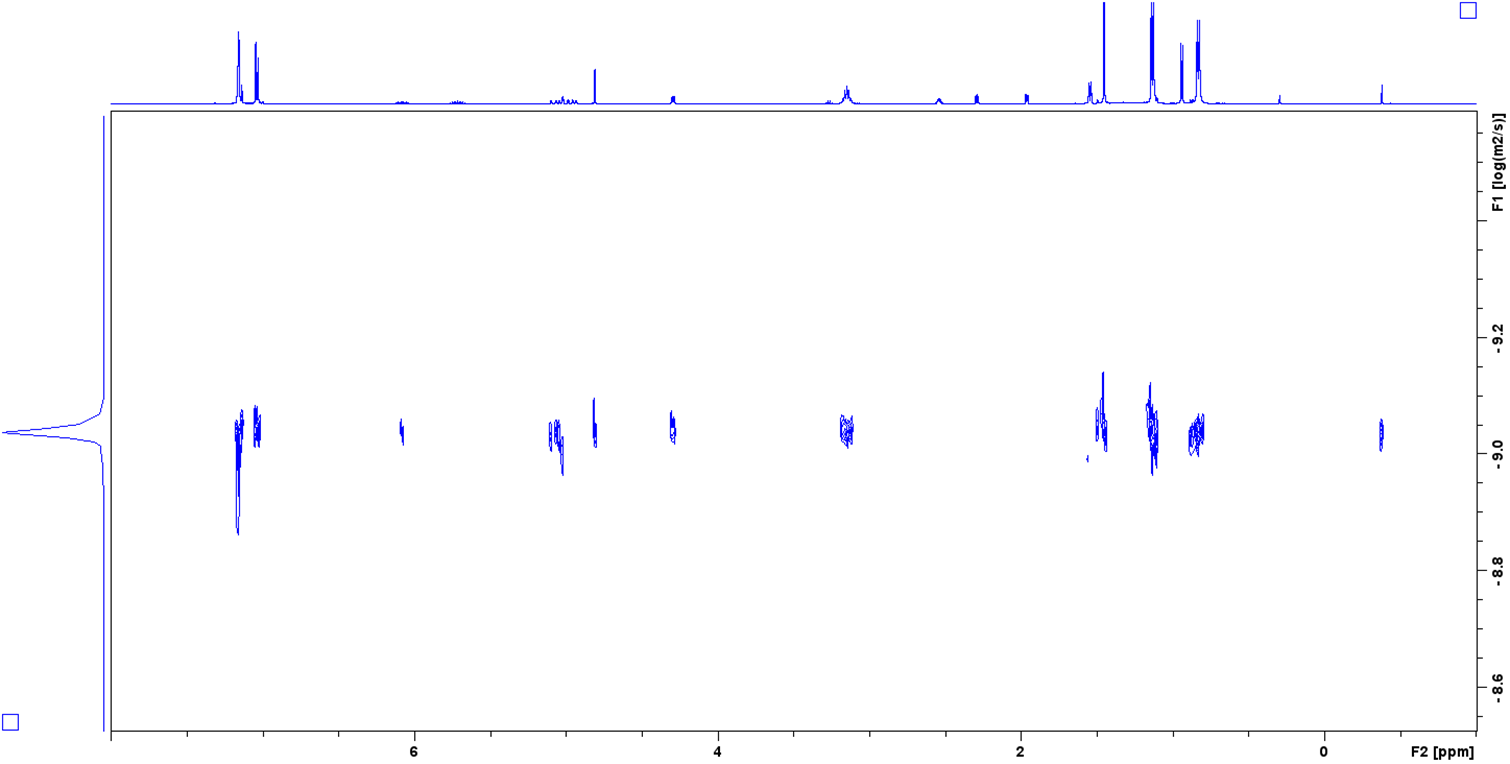


**Figure S6:** DOSY ^1^H NMR spectrum of **4** with propene and propylene oxide in C_6_D_6_. All peaks arising from **4** appear at the same diffusion coefficient, including the hydroxide and alkoxide peaks.

**Method B (in d_8_-THF):**

In a glovebox, [^Dipp^NacnacMg]_2_ (10.9 mg, 12.3 μmol, 1 eq) was dissolved in *d*_8_-THF (0.5 mL) and transferred to a J Youngs tap NMR tube. Propylene oxide (1.4 mg, 24.7 μmol, 14.3 μL of a 100 mg mL^-1^ solution in C_6_D_6_, 2 eq) was subsequently added. Upon shaking, the solution changed colour from orange to colourless. NMR spectroscopic analysis of the reaction mixture showed signals corresponding to oxo-bridged [(^Dipp^NacnacMg)_2_O•*d*_8_-THF_2_] **8**•*d*_8_-THF_2_ and excess PO. After 5 days at room temperature, colourless needle crystals formed in the NMR tube. NMR spectroscopic analysis of the reaction mixture and single crystal XRD confirmed the consumption of **8**•*d*_8_-THF_2_ and production of heteroleptic [^Dipp^NacnacMg(OH)(OCH_2_CHCH_2_)Mg^Dipp^Nacnac] **4** (Figure S97).

Complex **4**: ^1^H NMR (500 MHz, *d*_8_-THF, 300K) δ 7.05 (dd, *J* = 8.1, 7.1 Hz, 4H, Ar-*H*), 6.94 (d, *J* = 7.6 Hz, 8H, Ar-*H*), 5.89 – 5.73 (m, 1H, OCH_2_C*H*CH_2_)^#^, 4.89 (m, 2H, OCH_2_CHC*H*_2_)^#^, 4.75 (s, 2H, Nacnac-C*H*), 4.06 (dt, *J* = 6.1, 1.4 Hz, 2H, OC*H*_2_CHCH_2_), 3.02 (hept, *J* = 6.8 Hz, 8H, C*H*(CH_3_)_2_), 1.39 (s, 12H, Nacnac-C*H*_3_), 1.00 (d, *J* = 6.8 Hz, 24H, CH(C*H*_3_)_2_), 0.73 (d, *J* = 6.8 Hz, 24H, CH(C*H*_3_)_2_), 0.54 (s, 1H, O*H*).

^13^C NMR (126 MHz, *d*_8_-THF, 300K) δ 169.8 (N*C*CH_3_), 148.0 (Ar-*C*), 143.3 (OCH_2_*C*HCH_2_), 143.1 (Ar-*C*), 126.0 (Ar-*C*), 124.4 (Ar-*C*), 113.6 (OCH_2_CH*C*H_2_), 95.5 (Nacnac-*C*H), 66.9 (O*C*H_2_CHCH_2_), 28.9 (Nacnac-*C*H_3_), 25.1 (Nacnac-*C*H_3_), 24.7 (CH_2_(*C*H_3_)_2_), 24.6 (CH_2_(*C*H_3_)_2_).

^#^Overlap with propene

^†^O*H* peak for [^Dipp^NacnacMgOH]_2_


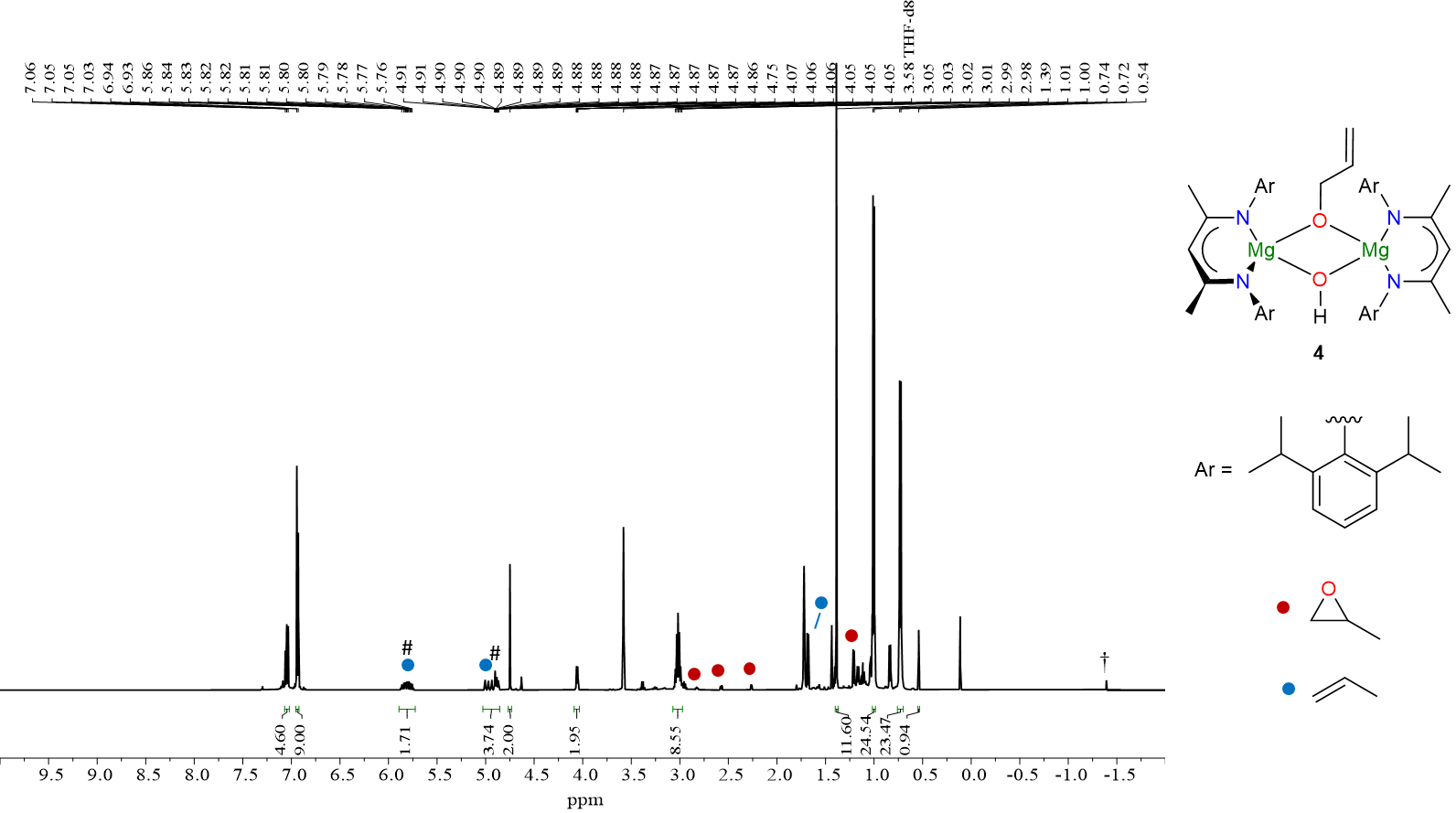


**Figure S7:** ^1^H NMR spectrum of **4** in d_8_-THF recorded at 500 MHz, 300 K. Propene and propylene oxide resonances are identified. ^#^Overlapping peak with propene, ^†^OH peak for [^Dipp^NacnacMgOH]_2_ **7**.


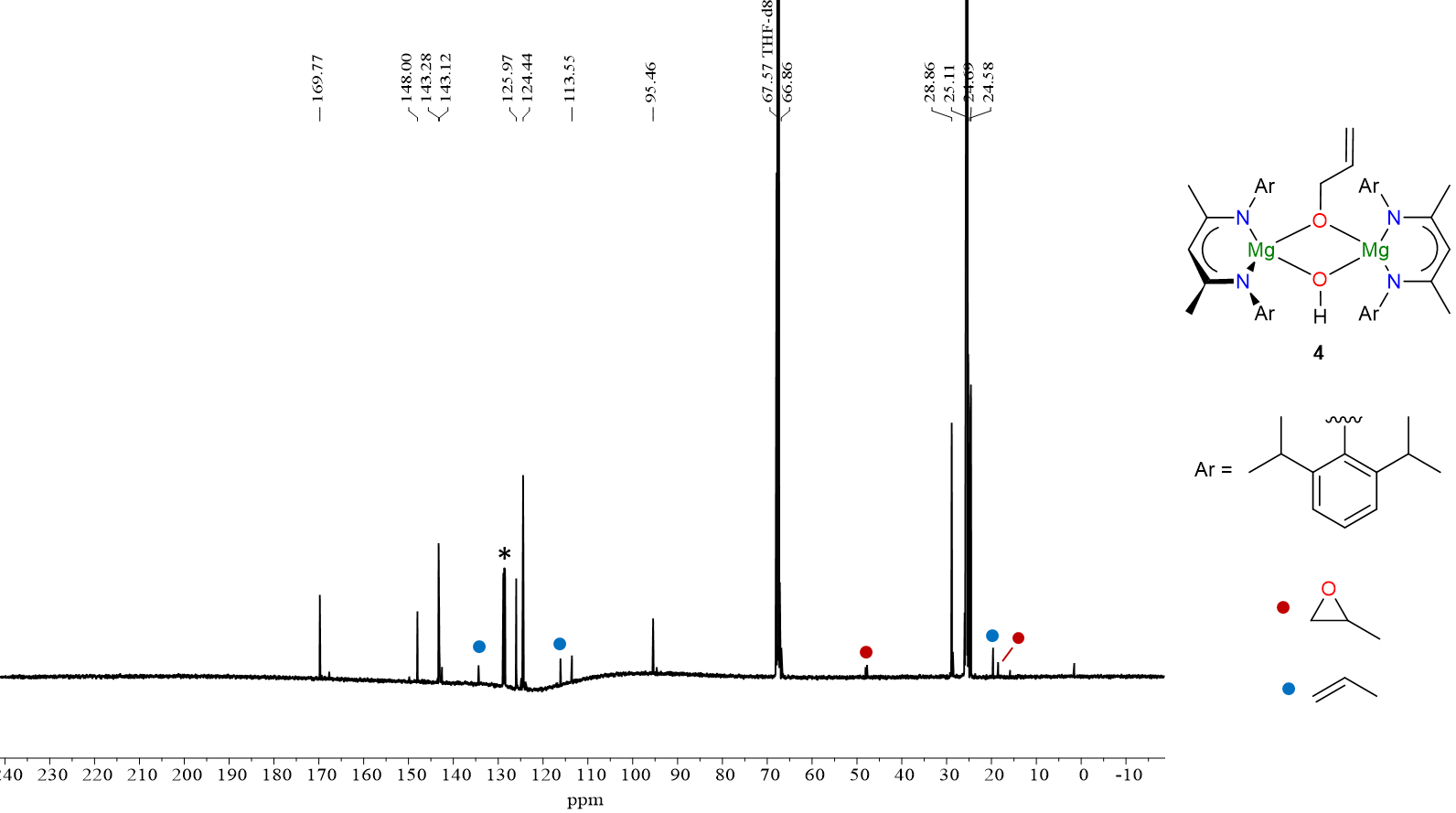


**Figure S8:** ^13^C{^1^H} NMR spectrum of **4** in d_8_-THF recorded at 126 MHz, 300 K. Propene and propylene oxide resonances are identified. *C_6_D_6_ present from PO stock solution (vide supra).

**Figure S9:** ^1^H-^1^H COSY NMR spectrum of **4** with propene and propylene oxide in d_8_-THF.

**Figure S10:** ^1^H-^13^C HSQC NMR spectrum of **4** with propene and propylene oxide in d_8_-THF.

**Figure S11:** ^1^H-^13^C HMBC NMR spectrum of **4** with propene and propylene oxide in d_8_-THF.


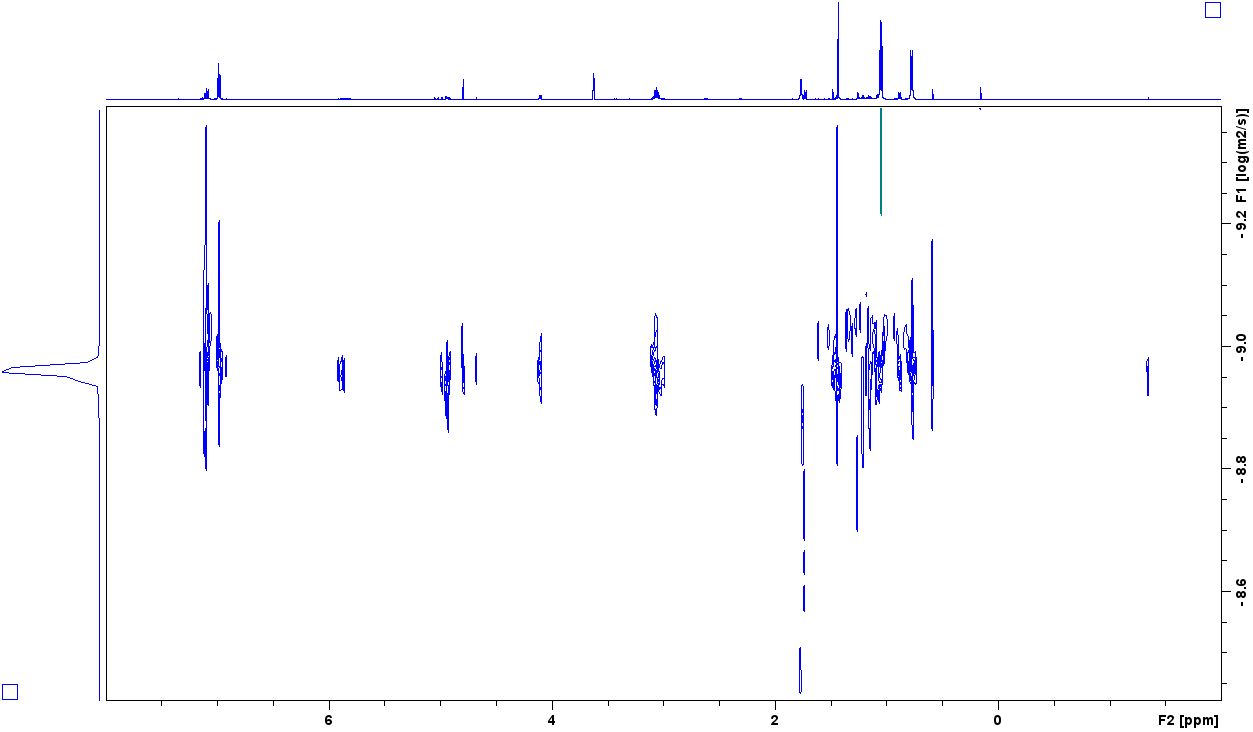


**Figure S12:** DOSY ^1^H NMR spectrum of **4** with propene and propylene oxide in d_8_-THF. All peaks arising from **4** appear at the same diffusion coefficient, including the hydroxide and alkoxide peaks.

**Synthesis of [^Dipp^NacnacMg(OD)(OCD_2_CDCD_2_)Mg^Dipp^Nacnac] (4D)**

In a glovebox, [^Dipp^NacnacMg]_2_ (11.1 mg, 12.6 μmol, 1 eq) was dissolved in C_6_D_6_ (0.6 mL) and transferred to a J Youngs tap NMR tube followed by the addition of *d*_6_-propylene oxide (3.22 mg, 50.2 μmol, 3.76 μL, 4 eq). Upon shaking, the solution turned from yellow to colourless and the reaction mixture was analysed by ^1^H and ^2^D NMR spectroscopy. Comparison of the ^1^H NMR spectrum of **4D** to **4** shows the absence of resonances attributed to the OH and OCH_2_CHCH_2_ group, as well as the resonances from propene and unreacted propylene oxide (see Figure S75). The ^2^D NMR spectrum shows resonances from the OD and OCD_2_CDCD_2_ groups, as well as *d*_6_-propene and *d*_6_-propylene oxide (see Figure S14). The NMR tube was left overnight at room temperature, which yielded clear, colourless crystals that were characterised by single crystal XRD analysis (Figure S98).

This synthetic protocol was repeated using [^Dipp^NacnacMg]_2_ (10.8 mg, 12.2 μmol, 1 eq) and *d*_6_-propylene oxide (3.12 mg, 48.7 μmol, 3.76 μL, 4 eq) in C_6_H_6_ (0.6 mL) spiked with C_6_D_6_ (1 µL) in a J Youngs tap NMR tube. The reaction mixture was analysed by ^2^D NMR spectroscopy (see Figure S15).

Complex **4D**: ^1^H NMR (500 MHz, C_6_D_6,_ 300 K) δ 7.20 – 7.12 (m, 4H, Ar-*H*)*, 7.04 (d, *J* = 7.6 Hz, 8H, Ar-*H*), 4.81 (s, 2H, Nacnac-C*H*), 3.15 (hept, *J* = 6.8 Hz, 8H, C*H*(CH_3_)_2_), 1.45 (s, 12H, Nacnac-C*H*_3_), 1.13 (d, *J* = 6.8 Hz, 24H, CH(C*H*_3_)_2_), 0.83 (d, *J* = 6.8 Hz, 24H CH(C*H*_3_)_2_).

^13^C NMR (201 MHz, C_6_D_6,_ 300 K) δ 169.3 (N*C*CH_3_), 147.2 (Ar-*C*), 142.6 (Ar-*C*), 125.5 (Ar-*C*), 124.0 (Ar-*C*), 95.2 (Nacnac-*C*H), 28.3 (*C*H_2_(CH_3_)_2_), 24.8 (Nacnac-*C*H_3_), 24.3 CH_2_(*C*H_3_)_2_), 24.2 (CH_2_(*C*H_3_)_2_).
^2^H NMR (123 MHz, C_6_D_6,_ 300 K) δ 6.05 (s, 1D, OCD_2_C*D*CD_2_), 5.05 (s, 1D, OCD_2_CDC*D*_2_), 4.93 (s, 1D, OCD_2_CDC*D*_2_)^#^, 4.21 (s, 2D, OC*D*_2_CDCD_2_), -0.38 (s, 1D, O*D*).

*Overlap with C_6_D_6_^#^Overlap with *d*_6_-propene


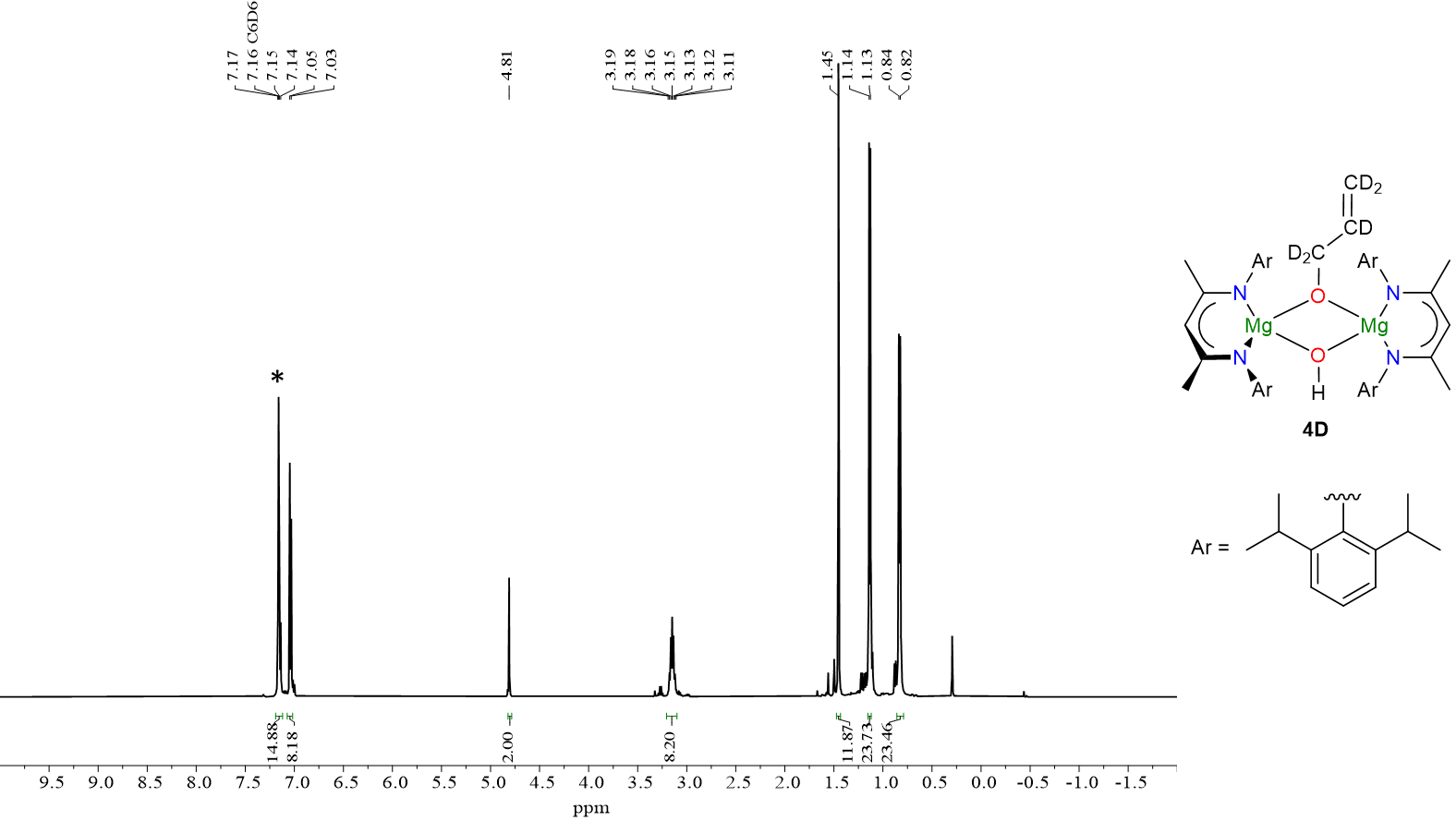


**Figure S13:** ^1^H NMR spectrum of **4D** in C_6_D_6_ recorded at 500 MHz, 300 K. *Overlapping peak with C_6_D_6_.


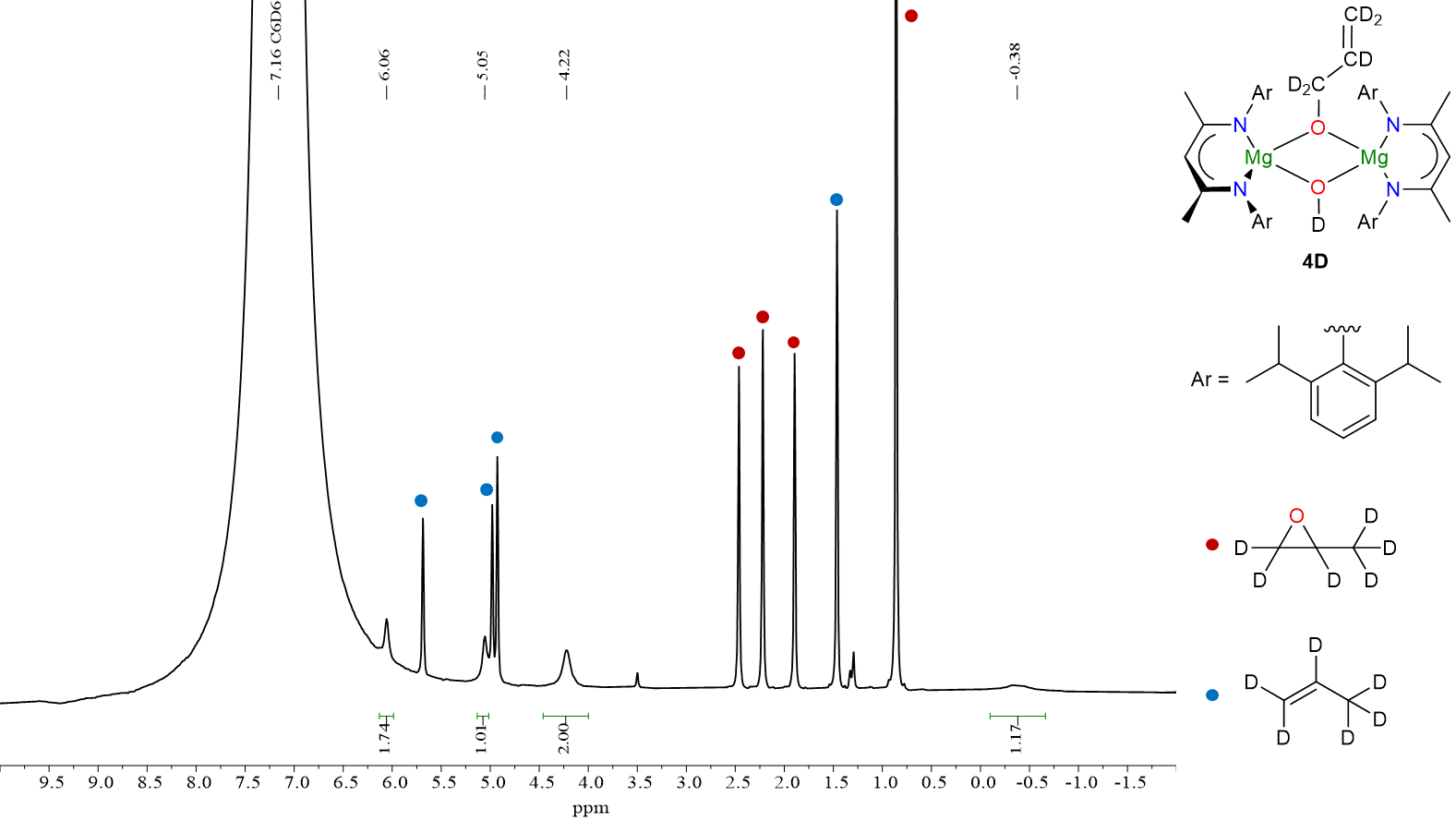


**Figure S14:** ^2^D NMR spectrum of **4D** in C_6_D_6_ recorded at 123 MHz, 300 K. d_6_-Propene and d_6_-propylene oxide are identified.


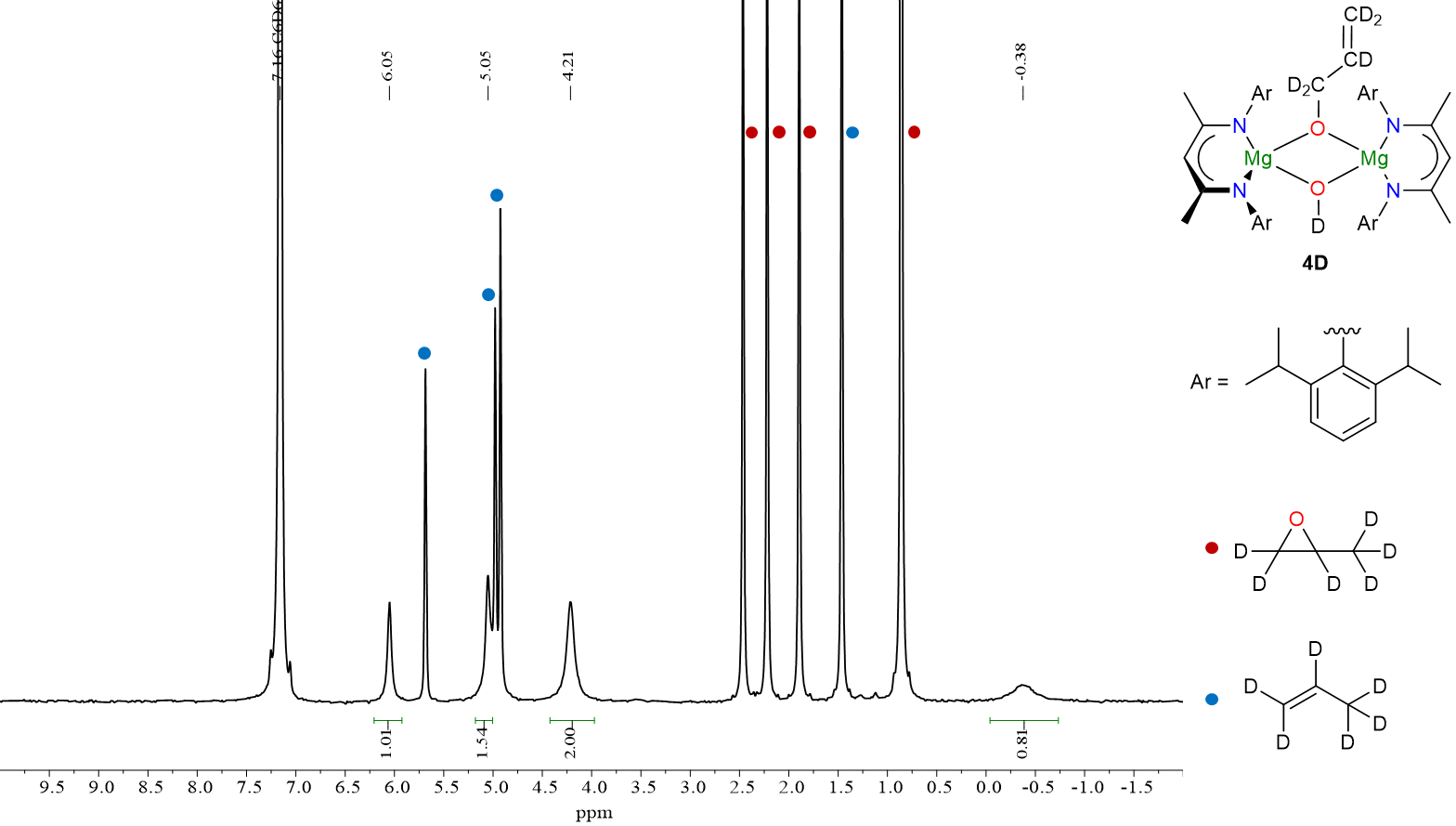


**Figure S15:** ^2^D NMR spectrum of **4D** in C_6_H_6_ with a C_6_D_6_ spike recorded at 123 MHz, 300 K. d_6_-Propene and d_6_-propylene oxide are identified.


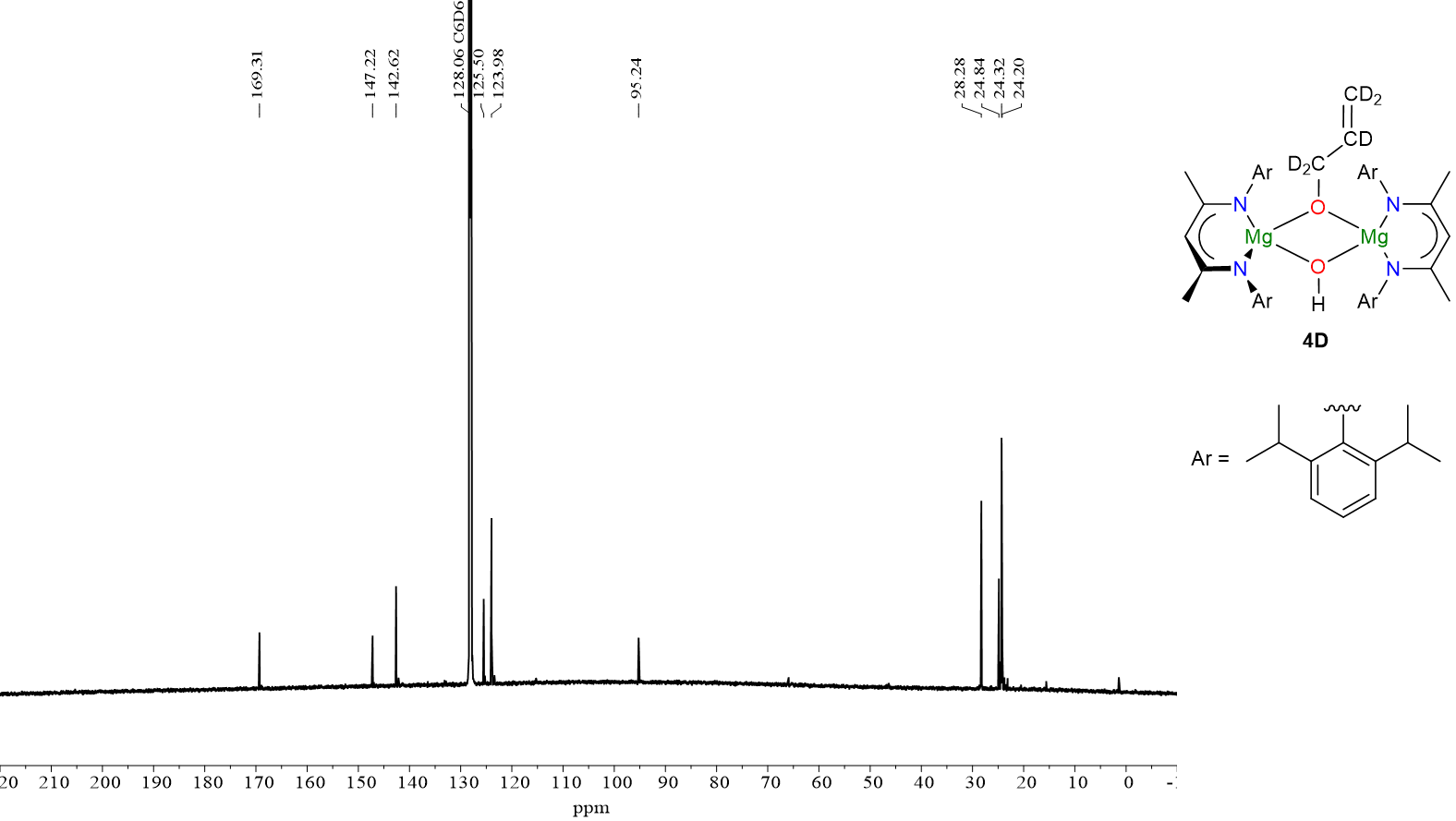


**Figure S16:** ^13^C{^1^H} NMR spectrum of **4D** in C_6_D_6_ recorded at 201 MHz, 300 K. Carbons coupled to deuterium were not identified due to weak signal intensities.

**Figure S17:** ^1^H-^1^H COSY NMR spectrum of **4D** with propene and propylene oxide in C_6_D_6_.

**Figure S18:** ^1^H-^13^C HSQC NMR spectrum of **4D** with propene and propylene oxide in C_6_D_6_.

**Figure S19:** ^1^H-^13^C HMBC NMR spectrum of **4D** with propene and propylene oxide in C_6_D_6_.

**Synthesis of [^Dep^NacnacMg(OH)(OCH_2_CHCH_2_)Mg^Dep^Nacnac] (5)**

In a glovebox, [^Dep^NacnacMg]_2_ (13.6 mg, 17.6 μmol, 1 eq) was dissolved in C_6_D_6_ (0.6 mL) and transferred to a J Youngs tap NMR tube. Propylene oxide (4.08 mg, 70.3 μmol, 4.01 μL, 4 eq) was added. Upon shaking, the solution turned from yellow to colourless and the reaction mixture was analysed by NMR spectroscopy.

To obtain crystals of **5** the reaction was repeated at a larger scale in toluene solvent:

[^Dep^NacnacMg]_2_ (103.1 mg, 134 μmol, 1 eq) was dissolved in toluene (10 mL). Propylene oxide (4.08 mg, 70.3 μmol, 4.01 μL, 4 eq) was added and the reaction mixture was stirred for 2 hours at room temperature. The reaction mixture was subsequently dried *in vacuo*, redissolved in toluene (1 mL), filtered and stored at -35 °C overnight which yielded colourless crystals that were characterised by single crystal XRD analysis (Figure S99).

Complex **5**: ^1^H NMR (500 MHz, C_6_D_6,_ 300 K) δ 7.14 (dd, *J* = 8.3, 6.9 Hz, 4H, Ar-*H*)*, 7.06 (d, *J* = 7.5 Hz, 8H, Ar-*H*), 5.80 – 5.64 (m, 1H, CH_2_C*H*CH_3_)^#^, 4.90 (dq, *J* = 6.5, 1.6 Hz, 1H, OCH_2_CHC*H*_2_), 4.87 (t, *J* = 1.6 Hz, 1H), 4.79 (s, 2H, Nacnac-C*H*), 3.97 (dt, *J* = 6.5, 1.6 Hz, 2H, OC*H*_2_CHCH_2_), 2.46 (dq, *J* = 15.2, 7.6 Hz, 8H, C*H*_2_CH_3_), 2.13 (dq, *J* = 15.2, 7.6 Hz, 8H, C*H*_2_CH_3_), 1.47 (s, 12H, Nacnac-C*H*_3_), 1.09 (t, *J* = 7.6 Hz, 24H, CH_2_C*H*_3_), -0.44 (s, 1H, O*H*).

^13^C NMR (201 MHz, C_6_D_6,_ 300 K) δ 168.6 (N*C*CH_3_), 147.6 (Ar-*C*), 142.3 (OCH_2_*C*HCH_2_), 137.2 (Ar-*C*), 126.2 (Ar-*C*), 124.4 (Ar-*C*), 113.1 (OCH_2_CH*C*H_2_), 94.7 (Nacnac-*C*H), 65.7 (O*C*H_2_CHCH_2_), 23.9 and 23.7 (*C*H_2_CH_3_ and Nacnac-*C*H_3_), 14.2 (CH_2_*C*H_3_).

*Overlap with C_6_D_6_

^#^Overlap with propene


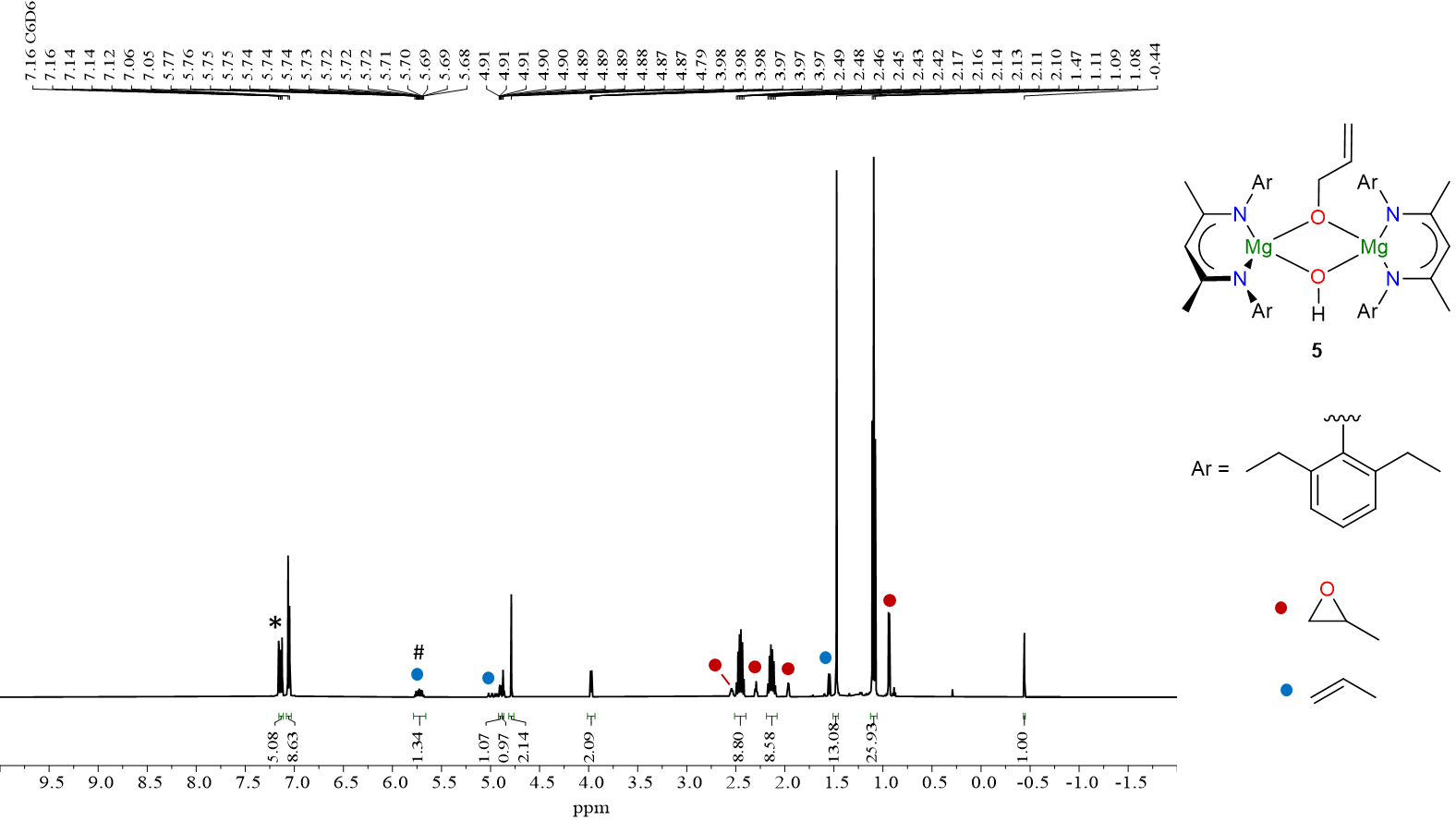


**Figure S20:** ^1^H NMR spectrum of **5** in C_6_D_6_ recorded at 500 MHz, 300 K. Propene and propylene oxide are identified. *Overlapping peak with C_6_D_6_, ^#^overlapping peak with propene.


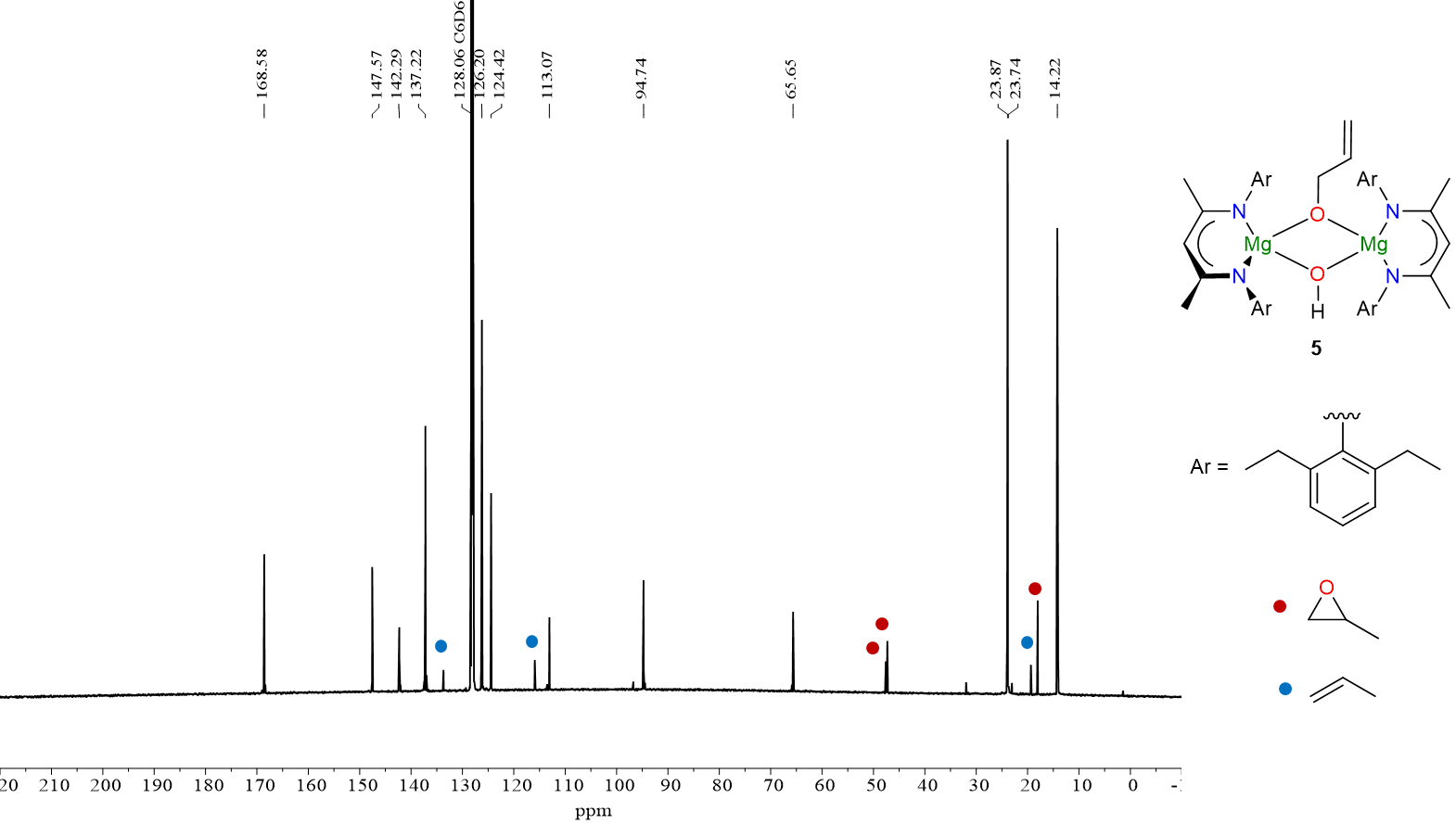


**Figure S21:** ^13^C{^1^H} NMR spectrum of **5** in C_6_D_6_ recorded at 201 MHz, 300 K. Propene and propylene oxide resonances are identified.

**Figure S22:** ^1^H-^1^H COSY NMR spectrum of **5** with propene and propylene oxide in C_6_D_6_.

**Figure S23:** ^1^H-^13^C HSQC NMR spectrum of **5** with propene and propylene oxide in C_6_D_6_.

**Figure S24:** ^1^H-^13^C HMBC NMR spectrum of **5** with propene and propylene oxide in C_6_D_6_.

**Synthesis of [^Mes^NacnacMg(OH)(OCH_2_CHCH_2_)Mg^Mes^Nacnac] (6)**

In a glovebox [^Mes^NacnacMg]_2_ (10.2 mg, 14.3 μmol, 1 eq) was dissolved in C_6_D_6_ (0.6 mL) and transferred to a J Youngs tap NMR tube, followed by the addition of propylene oxide (3.32 mg, 57.2 μmol, 4.00 μL, 4 eq). Upon shaking, the solution turned from yellow to colourless and the reaction mixture was analysed by NMR spectroscopy.

Complex **6**: ^1^H NMR (500 MHz, C_6_D_6,_ 300 K) δ 6.84 (s, 8H, Ar-*H*), 5.77 – 5.66 (m, 1H, OCH_2_C*H*CH_2_)^#^, 4.84 (dq, *J* = 10.3, 1.7 Hz, 1H, OCH_2_CHC*H*_2_), 4.82 (s, 2H, Nacnac-C*H*), 4.80 (dq, *J* = 17.2, 1.7 Hz, 1H, OCH_2_CHC*H*_2_), 3.98 (dt, *J* = 5.3, 1. Hz, 2H, OC*H*_2_CHCH_2_), 2.38 (s, 12H, Ar-C*H*_3_), 1.94 (s, 24H, Ar-C*H*_3_), 1.52 (s, 12H, Nacnac-C*H*_3_), -0.41 (s, 1H).

^13^C NMR (201 MHz, C_6_D_6,_ 300 K) δ 168.2 (N*C*CH_3_), 146.2 (Ar-*C*), 142.2 (OCH_2_*C*HCH_2_), 132.2 (Ar-*C*), 131.6 (Ar-*C*), 129.6 (Ar-*C*), 113.0 (OCH_2_CH*C*H_2_), 94.6 (Nacnac-*C*H), 65.2 (O*C*H_2_CHCH_2_), 23.2 (Nacnac-*C*H_3_), 21.2 (Ar-*C*H_3_), 18.4 (Ar-*C*H_3_).

^#^Overlap with propene


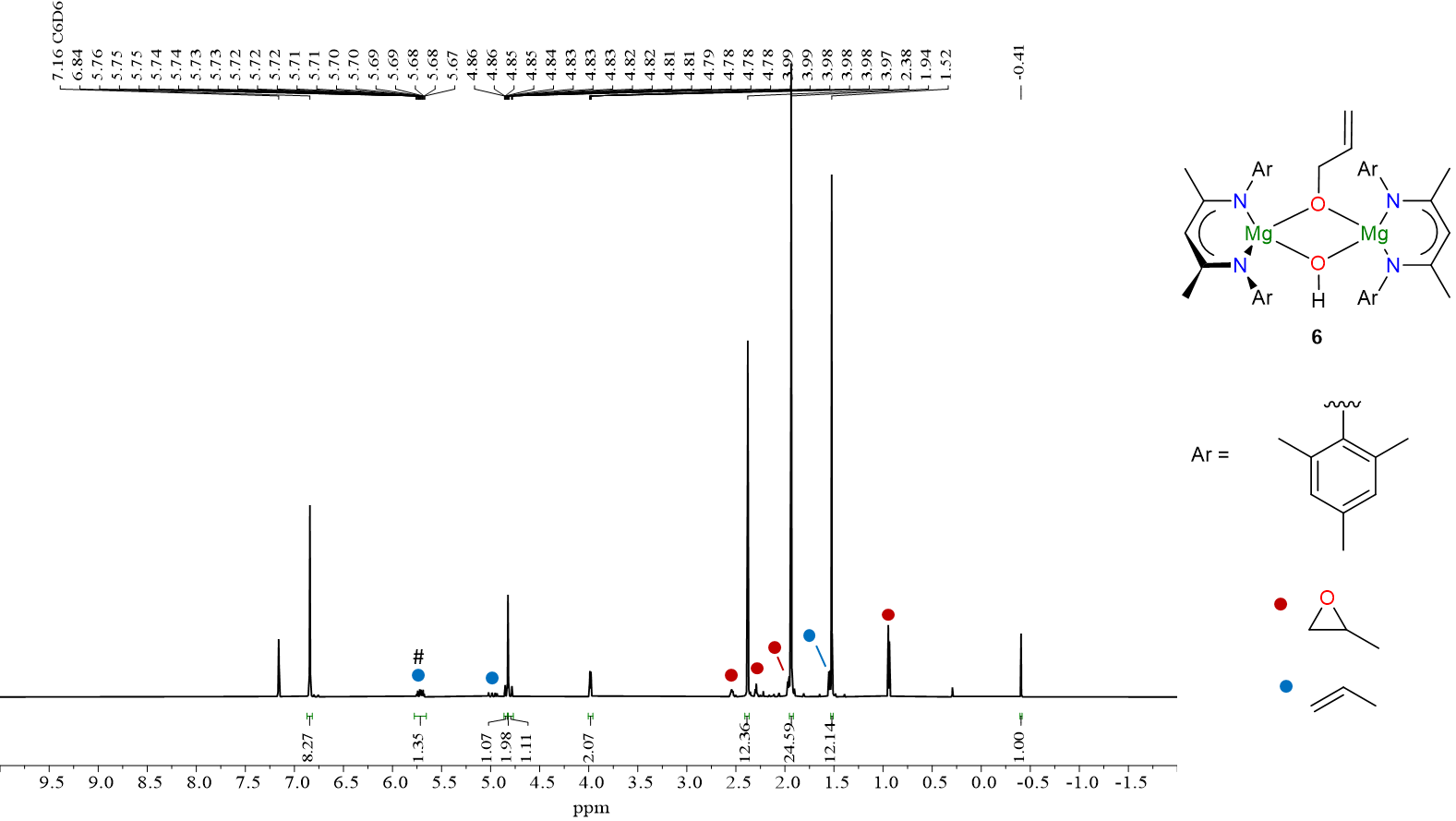


**Figure S25:** ^1^H NMR spectrum of **6** in C_6_D_6_ recorded at 500 MHz, 300 K. Propene and propylene oxide resonances are identified. ^#^Overlapping peak with propene.


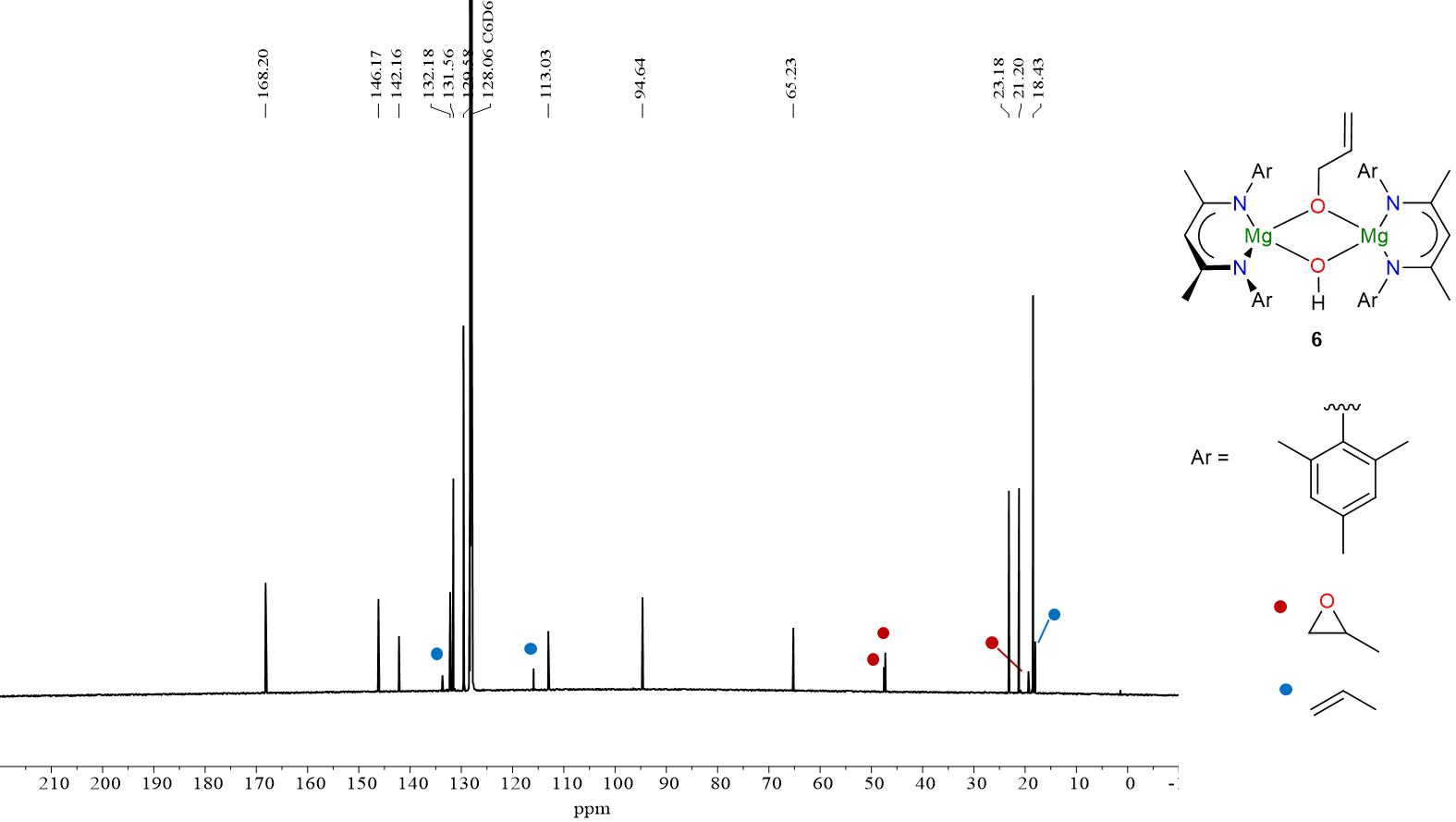


**Figure S26:** ^13^C{^1^H} NMR spectrum of **6** in C_6_D_6_ recorded at 201 MHz, 300 K. Propene and propylene oxide resonances are identified.

**Figure S27:** ^1^H-^1^H COSY NMR spectrum of **6** with propene and propylene oxide in C_6_D_6_.

**Figure S28:** ^1^H-^13^C HSQC NMR spectrum of **6** with propene and propylene oxide in C_6_D_6_.

**Figure S29:** ^1^H-^13^C HMBC NMR spectrum of **6** with propene and propylene oxide in C_6_D_6_.

**Literature synthesis of [(^Dipp^NacnacMg)_2_O] (8)**

[^Dipp^NacnacMg]_2_ (210.9 mg, 239 μmol, 1 eq) was dissolved in toluene (10 mL) and cooled to -30 °C. The head space was evacuated, backfilled with N_2_O (excess), sealed and warmed to room temperature over 30 minutes resulting in a milky white suspension. The suspension was dried *in vacuo*, washed with hexane (10 mL, 60 min) and filtered. The hexane supernatant was dried *in vacuo* to give **8** (125.9 mg, 58%) with about 10% [^Dipp^NacnacMgOH]_2_ impurity by ^1^H NMR spectroscopy (as previously noted).^[7]^

Analysis in *d*_8_-THF gave [(^Dipp^NacnacMg)_2_O•*d*_8_-THF]_2_ (8•*d*_8_-THF_2_) which was in agreement for reported [^Dipp^NacnacMg•THF]_2_ (without proton signals for coordinated THF).^[7]^

Complex **8**•*d*_8_-THF_2_: ^1^H NMR (500 MHz, *d*_8_-THF) δ 7.01 – 6.88 (m, 12H, Ar-*H*), 4.63 (s, 2H, Nacnac-C*H*), 2.97 (hept, *J* = 6.9 Hz, 8H, C*H*(CH_3_)_2_), 1.40 (s, 12H, Nacnac-C*H*_3_), 1.03 (d, *J* = 6.9 Hz, 24H, CH(C*H*_3_)_2_), 0.82 (d, *J* = 6.9 Hz, 24H, CH(C*H*_3_)_2_).

^13^C NMR (126 MHz, C_6_D_6,_ 300 K) δ 168.0 (N*C*CH_3_), 148.7 (Ar-*C*), 142.9 (Ar-*C*), 124.9 (Ar-*C*), 123.9 (Ar-*C*), 94.7 (Nacnac-*C*H), 28.5 (*C*H_2_(CH_3_)_2_), 25.8, 24.9, 24.6 (Nacnac-*C*H_3_, 2 x CH_2_(*C*H_3_)_2_).


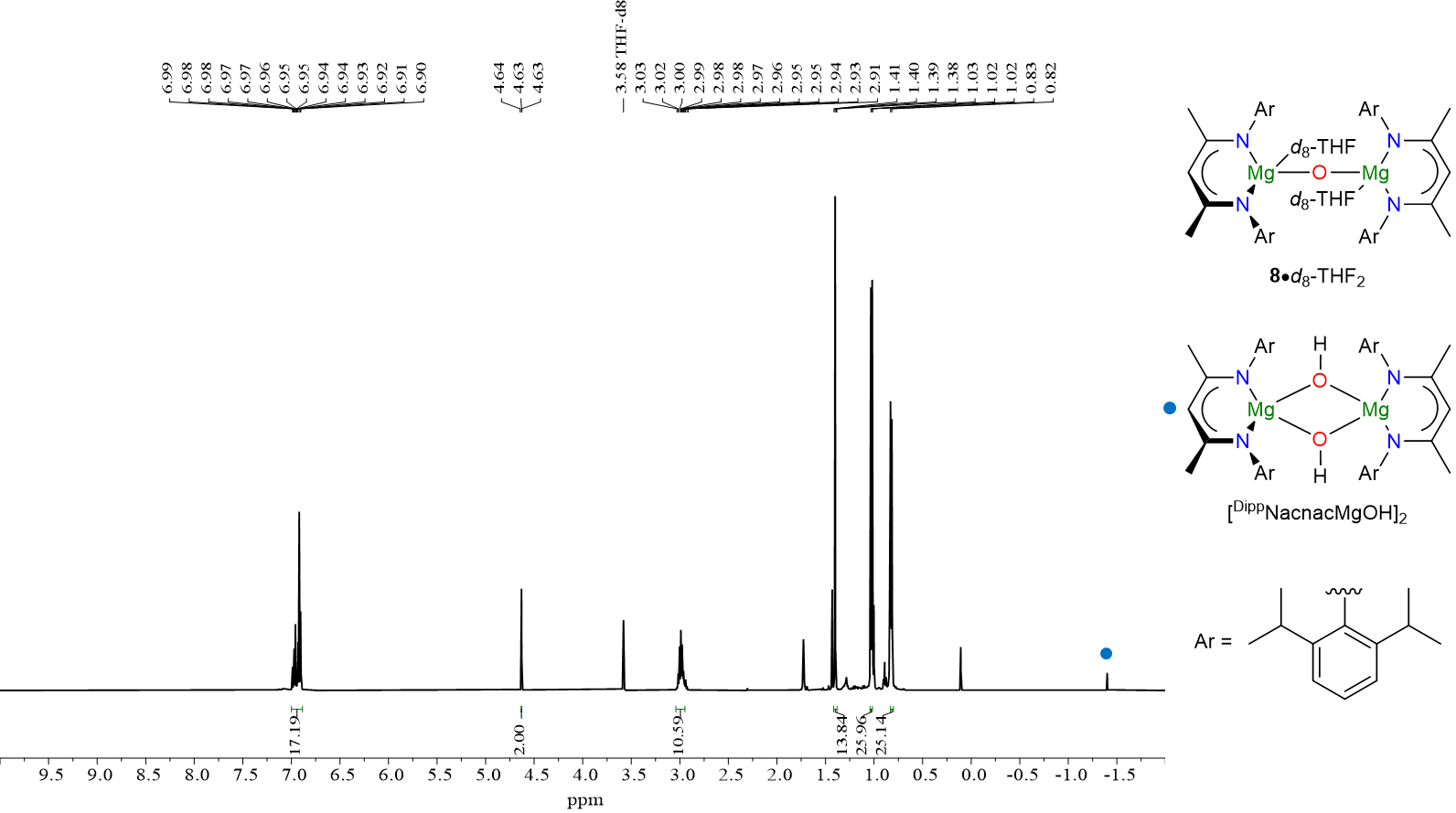


**Figure S30:** ^1^H NMR spectrum of **8**•d_8_-THF_2_ in d_8_-THF recorded at 500 MHz, 300 K. The OH peak for [^Dipp^NacnacMgOH]_2_ is identified with a blue dot.


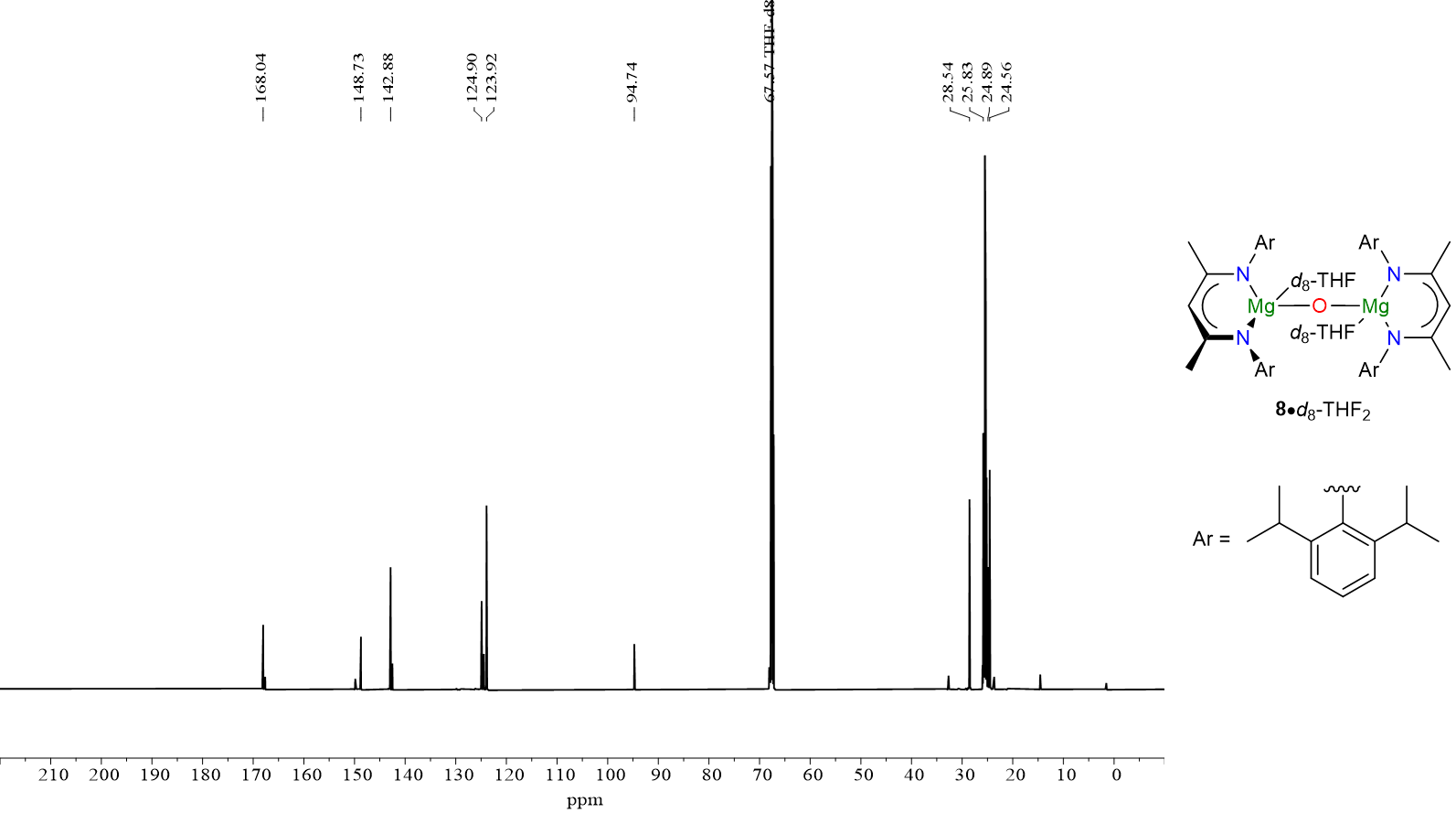


**Figure S31:** ^13^C{^1^H} NMR spectrum of **8**•d_8_-THF_2_ in d_8_-THF recorded at 126 MHz, 300 K (trace [^Dipp^NacnacMgOH]_2_ present).

**Alternative NMR scale synthesis of [(^Dipp^NacnacMg)_2_O•THF_2_] (8•THF_2_)**

In a glovebox, [^Dipp^NacnacMg]_2_ (11.6 mg, 13.1 μmol, 1 eq) was dissolved in THF (0.5 mL) and transferred to a J Youngs tap NMR tube followed by the addition of propylene oxide (0.76 mg, 13.1 μmol, 7.6 μL of a 100 mg mL^-1^ solution in C_6_D_6_, 1 eq). Upon shaking and leaving the reaction to stand for 60 min, a colour change from orange to colourless was observed. The solution was dried *in vacuo*. The reaction mixture was subsequently redissolved in *d*_8_-THF and analysed by NMR spectroscopy.

Analysis in *d*_8_-THF gave good agreement with reported [^Dipp^NacnacMg•THF]_2_ (**8**•THF_2_).^[7]^

Complex **8**•THF_2_: ^1^H NMR (500 MHz, *d*_8_-THF) δ 7.01 – 6.88 (m, 12H, Ar-*H*), 4.63 (s, 2H, Nacnac-C*H*), 3.64 – 3.60 (m, 8H, OC*H*_2_CH_2_), 2.97 (hept, *J* = 6.9 Hz, 8H, C*H*(CH_3_)_2_), 1.82 – 1.74 (m, 8H, OCH_2_C*H*_2_), 1.40 (s, 12H, Nacnac-C*H*_3_), 1.03 (d, *J* = 6.9 Hz, 24H, CH(C*H*_3_)_2_), 0.82 (d, *J* = 6.9 Hz, 24H, CH(C*H*_3_)_2_).

^13^C NMR (126 MHz, C_6_D_6,_ 300 K) δ 168.1 (N*C*CH_3_), 148.7 (Ar-*C*), 142.9 (Ar-*C*), 124.9 (Ar-*C*), 123.9 (Ar-*C*), 94.7 (Nacnac-*C*H), 28.5 (*C*H_2_(CH_3_)_2_), 26.6, 24.9, 24.6 (Nacnac-*C*H_3_, 2 x CH_2_(*C*H_3_)_2_).


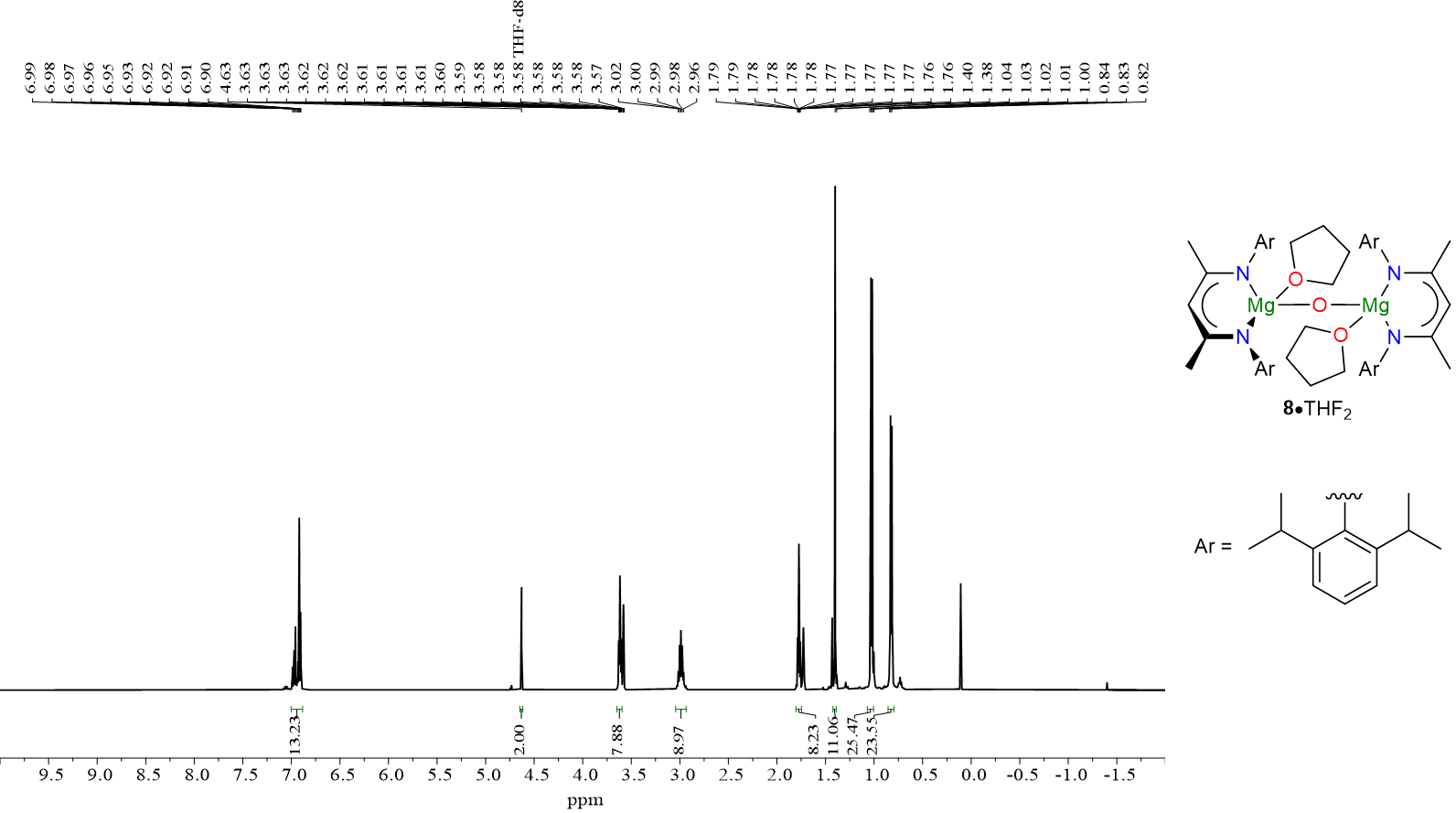


**Figure S32:** ^1^H NMR spectrum of **8**•THF_2_ in d_8_-THF recorded at 500 MHz, 300 K.


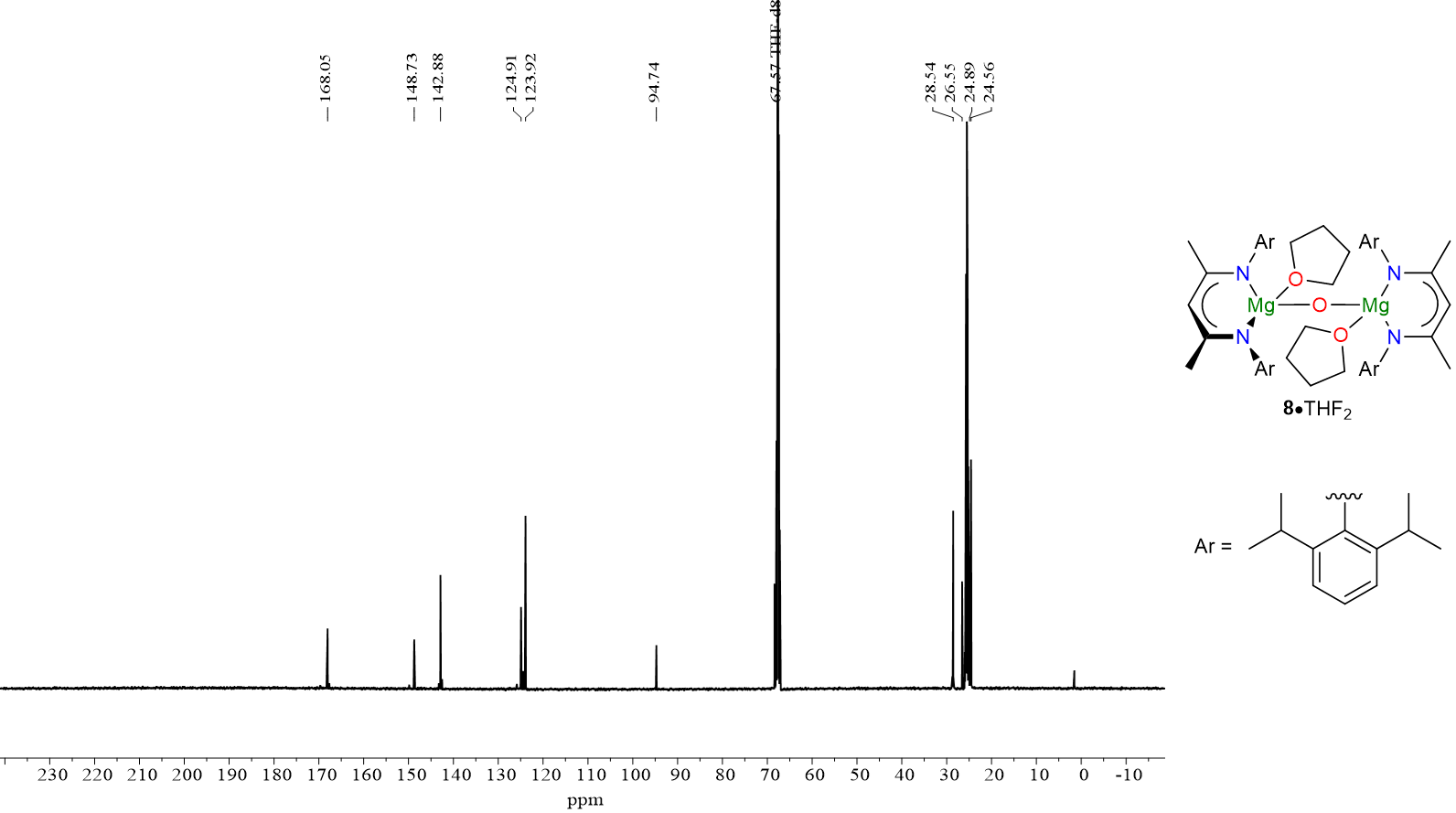


**Figure S33:** ^13^C{^1^H} NMR spectrum of **8**•THF_2_ in d_8_-THF recorded at 126 MHz, 300 K.

**Synthesis of [^Dipp^NacnacMg(OH)(OCH_2_C(CH_2_)(CH_3_))Mg^Dipp^Nacnac] (9)**

In a glovebox, [^Dipp^NacnacMg]_2_ (11.1 mg, 12.6 μmol, 1 eq) was dissolved in C_6_D_6_ (0.6 mL) and transferred to a J Youngs tap NMR tube. Isobutylene oxide (3.6 mg, 50.2 μmol, 4.46 μL, 4 eq) was subsequently added. Upon shaking the solution turned from yellow to colourless, and the reaction mixture was analysed by NMR spectroscopy. The NMR tube was left overnight at room temperature, which yielded colourless crystals that were characterised by single crystal XRD analysis (Figure S100).

Complex **9**: ^1^H NMR (500 MHz, C_6_D_6,_ 300 K) δ 7.18 – 7.13 (m, 4H, Ar-*H*)*, 7.04 (d, *J* = 7.6 Hz, 8H, Ar-*H*), 5.00 (br. d, *J* = 1.9 Hz, 1H, OCH_2_C(C*H*_2_)(CH_3_)), 4.93 (br. d, *J* = 1.9 Hz, 1H, OCH_2_C(C*H*_2_)(CH_3_)), 4.84 (s, 2H, Nacnac-C*H*), 4.22 (br. s, 2H, OC*H*_2_C(CH_2_)(CH_3_)), 3.16 (hept, *J* = 6.8 Hz, 8H, C*H*(CH_3_)_2_), 1.65 (s, 3H, OCH_2_C(CH_2_)(C*H*_3_)), 1.45 (s, 12H, Nacnac-C*H*_3_), 1.13 (d, *J* = 6.8 Hz, 24H, CH(C*H*_3_)_2_), 0.81 (d, *J* = 6.8 Hz, 24H, CH(C*H*_3_)_2_), -0.34 (s, 1H, O*H*).

^13^C NMR (126 MHz, C_6_D_6,_ 300 K) δ 169.4 (N*C*CH_3_), 147.6 (Ar-*C*), 147.4 (OCH_2_*C*(CH_2_)(CH_3_)), 142.7 (Ar-*C*), 125.5 (Ar-*C*), 124.0 (Ar-*C*), 105.4 (OCH_2_C(*C*H_2_)(CH_3_)), 95.6 (Nacnac-*C*H), 68.1 (O*C*H_2_C(CH_2_)(CH_3_)), 28.4 (*C*H(CH_3_)_2_), 24.9 (N*C*CH_3_), 24.4 and 24.2 (2 x CH(*C*H_3_)_2_), 20.2 (OCH_2_C(CH_2_)(*C*H_3_)).

*Overlap with C_6_D_6_ solvent peak

^#^Residual diethyl ether from [^Dipp^NacnacMg]_2_ purification


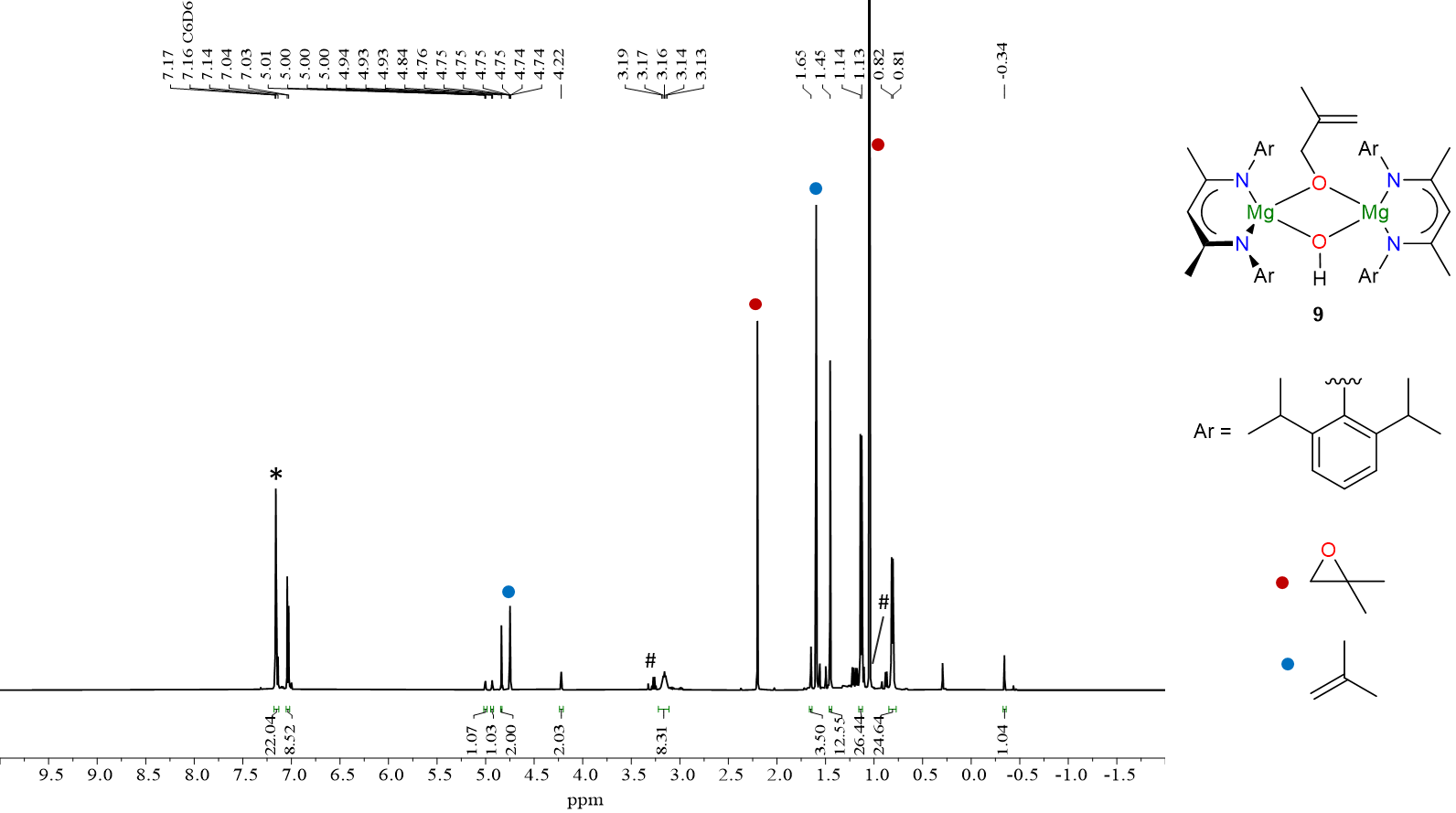


**Figure S34:** ^1^H NMR spectrum of **9** in C_6_D_6_ recorded at 500 MHz, 300 K. Isobutene and isobutylene oxide resonances are identified. *Overlapping peak with C_6_D_6_. ^#^Residual diethyl ether.


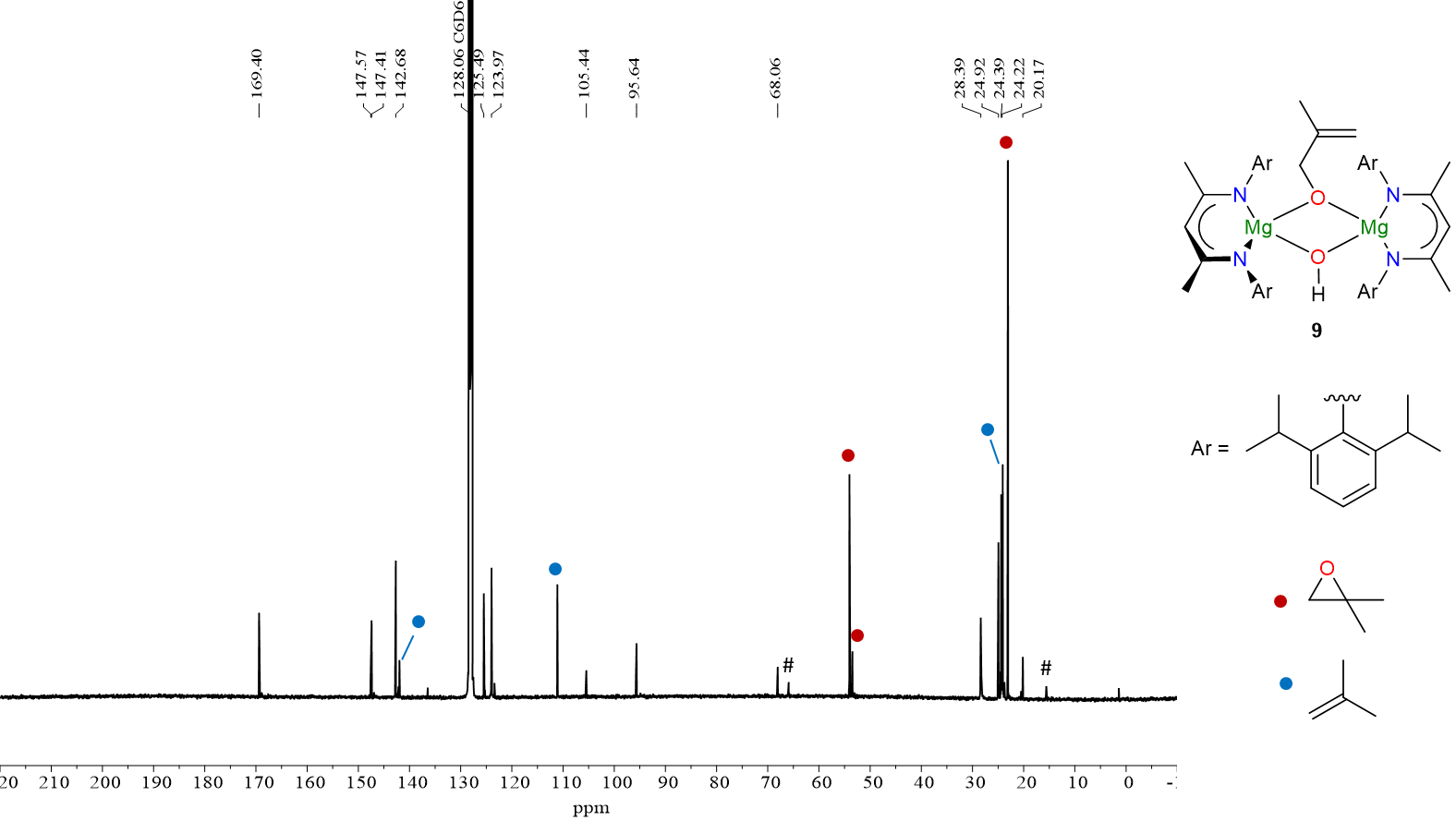


**Figure S35:** ^13^C{^1^H} NMR spectrum of **9** in C_6_D_6_ recorded at 126 MHz, 300 K. Isobutene and isobutylene oxide resonances are identified. ^#^Residual diethyl ether.

**Figure S36:** ^1^H-^1^H COSY NMR spectrum of **9** with isobutene and isobutylene oxide in C_6_D_6_.

**Figure S37:** ^1^H-^13^C HSQC NMR spectrum of **9** with isobutene and isobutylene oxide in C_6_D_6_.

**Figure S38:** ^1^H-^13^C HMBC NMR spectrum of **9** with isobutene and isobutylene oxide in C_6_D_6_.


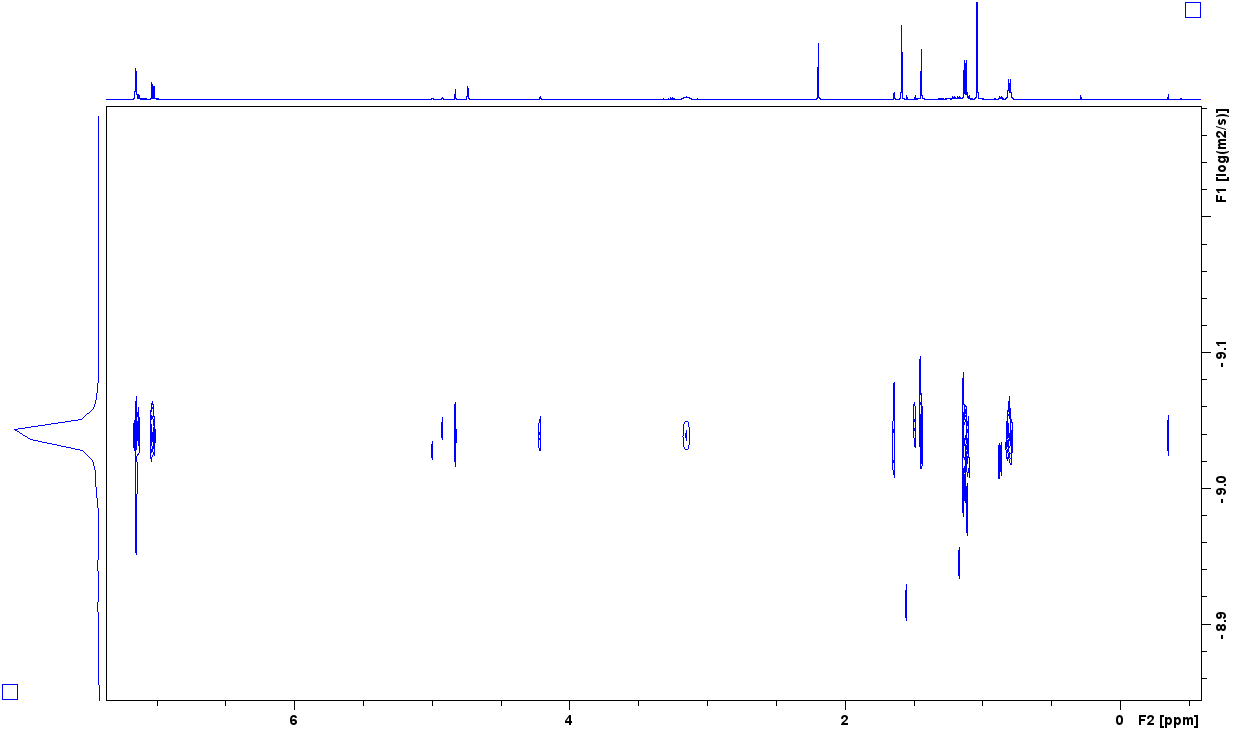


**Figure S39:** DOSY ^1^H NMR spectrum of **9** with isobutene and isobutylene oxide in C_6_D_6_. All peaks belonging to **9** appear at the same diffusion coefficient including the hydroxide and alkoxide peaks.

**Synthesis of [^Dipp^NacnacMg(OH)(OCH_2_CH=CHCH_2_)Mg^Dipp^Nacnac] (10a(-*cis*)/10b(-*trans*))**

In a glovebox, [^Dipp^NacnacMg]_2_ (10.5 mg, 11.9 μmol, 1 eq) was dissolved in C_6_D_6_ (0.6 mL) and transferred to a J Youngs tap NMR tube. Butylene oxide (3.4 mg, 47.6 μmol, 4.00 μL, 4 eq) was subsequently added. The solution was shaken, which gave a colour change from yellow to colourless. The reaction mixture was analysed by NMR spectroscopy.

The ^1^H and ^13^C NMR spectra show the presence of two products, assigned as the *cis*- and *trans*- isomers (**10** and **10b**). This was further supported by 2D COSY, HSQC and HMBC experiments. Based on the distinctive OH resonances, the relative integrals show that these species are present in a 3:5 ratio of **10a**:**10b**. Assignments are based on the couplings of the alkenyl OCH_2_CH=CHCH_2_ unit (typically ~10Hz for *cis*- and ~16 Hz for *trans*-alkenes), in conjunction with the relative integrals and the COSY, HSQC and HMBC analysis.

Complex **10a**: ^1^H NMR (500 MHz, C_6_D_6,_ 300 K) δ 7.20 – 7.12 (m, 4H, Ar-*H*)*^†^, 7.04 (d, *J* = 7.6 Hz, 8H, Ar-*H*)^†^, 5.59 (dtq, *J* 11.3, 5.6, 1.7 Hz, 1H, OCH_2_C*H*CHCH_3_)^†^, 5.43 (dqt, *J* = 11.3, 6.9, 1.9 Hz, 1H, OCH_2_CHC*H*CH_2_), 4.82 (s, 2H, Nacnac-C*H*), 4.42 (dt, *J* = 5.6, 1.9 Hz, 2H, OC*H*_2_CHCHCH_2_), 3.16 (m, 8H, C*H*(CH_3_)_2_)^†^, 1.57 (dd, *J* = 6.9, 1.7 Hz, 3H, OCH_2_CHCHC*H*_3_), 1.46 (s, 12H, Nacnac-C*H*_3_), 1.14 (d, *J* = 6.8 Hz, 24H, CH(C*H*_3_)_2_)^†^, 0.84 (d, *J* = 6.8 Hz, 24H CH(C*H*_3_)_2_)^†^, -0.38 (s, 1H, O*H*).

^13^C NMR (201 MHz, C_6_D_6,_ 300 K) δ 169.3/169.2 (N*C*CH_3_), 147.3/142.6 (Ar-*C*)^†^, 136.4 (OCH_2_*C*HCHCH_3_), 125.5/124.0 (Ar-*C*)^†^, 121.7 (OCH_2_CH*C*HCH_3_), 95.3/95.2 (Nacnac-*C*H), 60.8 (O*C*H_2_CHCHCH_3_), 28.34/28.27 (*C*H(CH_3_)), 24.84/24.81 (Nacnac-*C*H_3_), 24.4/24.2/24.1 (CH(*C*H_3_))^†^, 13.8 (OCH_2_CHCH*C*H_3_).

Complex **10b**: ^1^H NMR (500 MHz, C_6_D_6,_ 300 K) δ 7.20 – 7.12 (m, 4H, Ar-*H*)*, 7.04 (d, *J* = 7.6 Hz, 8H, Ar-*H*)^†^, 5.73 (dtq, *J* = 14.4, 6.4, 1.6 Hz, 1H, OCH_2_C*H*CHCH_3_)^#^, 5.53 (dqt, 14.4, 6.4, 1.3 Hz, 1H, OCH_2_CHC*H*CH_3_)^†^, 4.80 (s, 2H, Nacnac-C*H*), 4.22 (dt, *J* = 6.4, 1.3 Hz, 2H, OC*H*_2_CHCHCH_3_), 3.16 (m, 8H, C*H*(CH_3_)_2_)^†^, 1.67 (dd, *J* = 6.4, 1.6 Hz, 3H, OCH_2_CHCHC*H*_3_), 1.45 (s, 12H, Nacnac-C*H*_3_), 1.14 (d, *J* = 6.8 Hz 24H, CH(C*H*_3_)_2_)^†^, 0.84 (d, *J* = 6.8 Hz, 24H, CH(C*H*_3_)_2_), -0.40 (s, 1H, O*H*).

^13^C NMR (201 MHz, C_6_D_6,_ 300 K) δ 169.3/169.2 (N*C*CH_3_), 147.3/142.6 (Ar-*C*)^†^, 134.8 (OCH_2_*C*HCHCH_3_), 126.1 (OCH_2_CH*C*HCH_3_), 125.5/124.0 (Ar-*C*)^†^, 95.3/95.2 (Nacnac-*C*H), 65.2 (O*C*H_2_CHCHCH_3­_), 28.34/28.27 (*C*H(CH_3_)), 24.84/24.81 (Nacnac-*C*H_3_), 24.4/24.2/24.1 (CH(*C*H_3_))^†^, 18.4 (OCH_2_CHCH*C*H_3_).

*Overlap with C_6_D_6_
^#^Overlap with 1-butene
^†^Overlap of **10a** and **10b**For ^13^C NMR x ppm/y ppm indicates the peak belongs to either **10a** or **10b**.


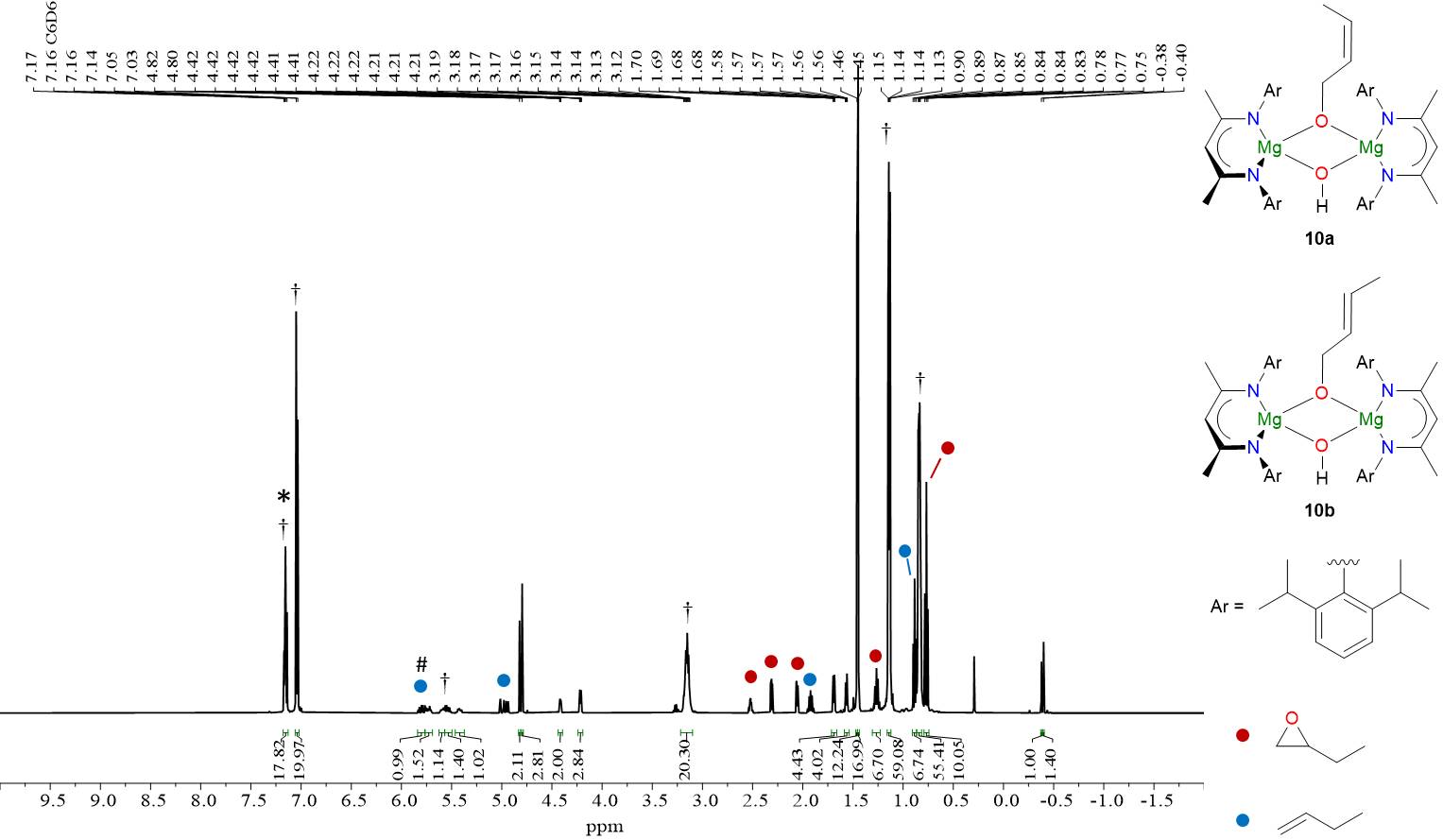


**Figure S40:** ^1^H NMR spectrum of **10a**/**10b** in C_6_D_6_ recorded at 500 MHz, 300 K. Butene and butylene oxide resonances are identified. *Overlapping peak with C_6_D_6_. ^#^Overlapping peak with butene. ^†^Overlapping peaks of **10a**/**10b**.


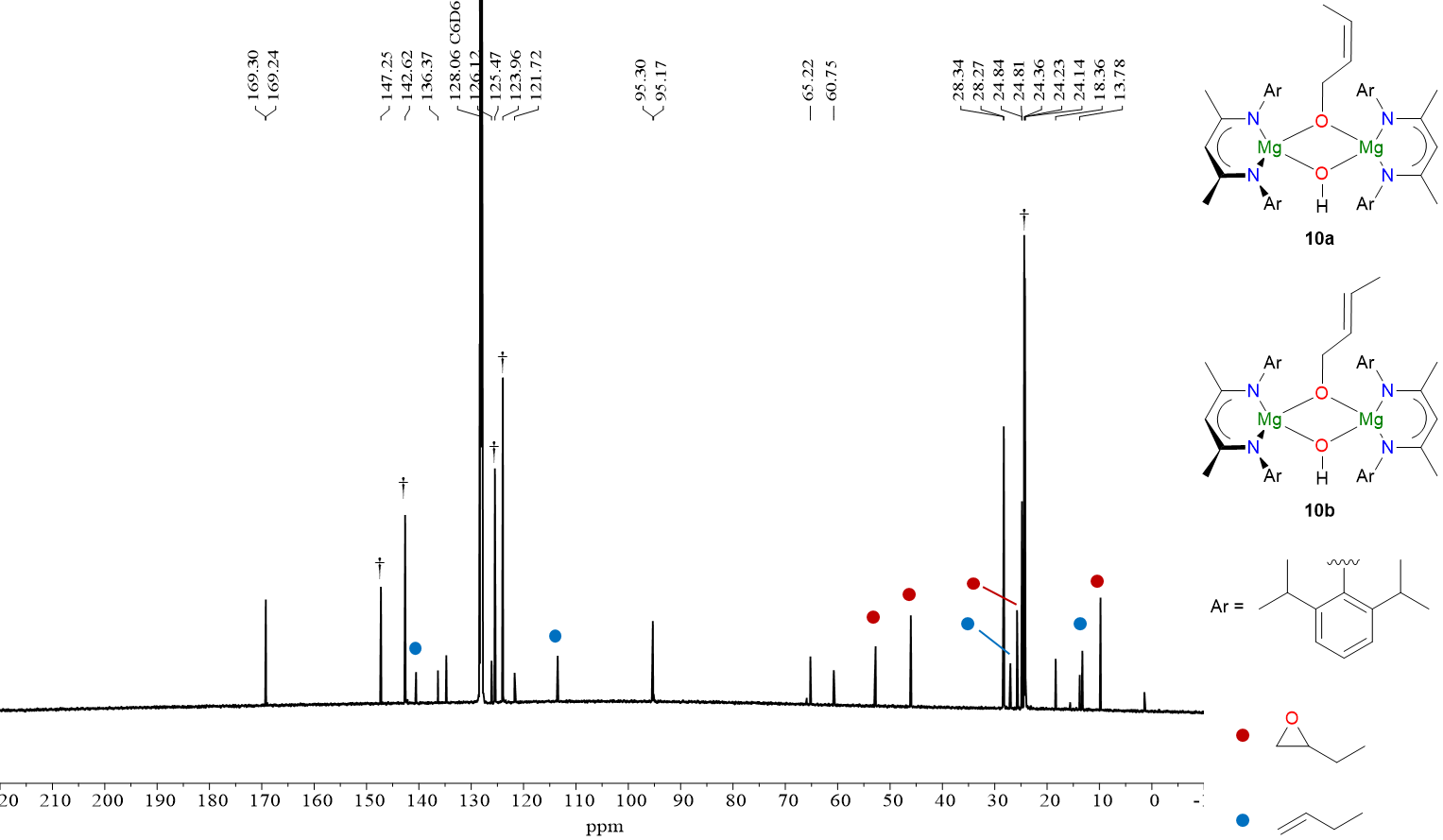


**Figure S41:** ^13^C{^1^H} NMR spectrum of **10a**/**10b** in C_6_D_6_ recorded at 201 MHz, 300 K. Butene and butylene oxide resonances are identified. ^†^Overlapping peak of **10a**/**10b**.

**Figure S42:** ^1^H-^1^H COSY NMR spectrum of **10a**/**10b** with butene and butylene oxide in C_6_D_6_.

**Figure S43:** ^1^H-^13^C HSQC NMR spectrum of **10a**/**10b** with butene and butylene oxide in C_6_D_6_.

**Figure S44:** ^1^H-^13^C HMBC NMR spectrum of **10a**/**10b** with butene and butylene oxide in C_6_D_6_.

### **Synthesis of [^Dipp^NacnacMg(OH)(OCH_2_CHCHCH_2_CH_2_CH_2_)Mg^Dipp^Nacnac] (11a)**

In a glovebox, [^Dipp^NacnacMg]_2_ (10.8 mg, 12.2 μmol, 1 eq) was dissolved in C_6_D_6_ (0.6 mL) and the solution was transferred to a J Youngs tap NMR tube. Cyclohexene oxide (2.4 mg, 24.2 μmol, 2.47 μL, 2 eq) was added. Upon shaking, the solution turned from yellow to colourless and the reaction mixture was analysed by NMR spectroscopy. The NMR tube was kept at room temperature for three days yielding colourless crystals that were characterised by single crystal XRD analysis as the alkoxide dimer **11b**.

N.B. Complex **11b** was previously synthesised from the addition of cyclohexene oxide to [^Dipp^NacnacMg(HMDS)] (HMDS=hexamethyldisilazane) and characterised by XRD analysis.^[27]^

Complex **11a**: ^1^H NMR (500 MHz, C_6_D_6,_ 300 K) δ 7.15 (d, *J* = 7.6 Hz, 4H, Ar-*H*)*, 7.13 – 6.98 (br. m, 8H, Ar-*H*), 5.82 (br. d, *J* = 10.0 Hz, 1H, Cy C*H*, OCC*H* alkene), 5.65 (br. d, *J* = 10.0 Hz, 1H, Cy C*H*, OCCHC*H* alkene), 4.82 (s, 2H, Nacnac-C*H*), 4.18 (br. s, 1H, Cy, C*H,* alkyl), 3.36 – 3.03 (br. m, 8H, C*H*(CH_3_)_2_), 2.14 – 2.08 (br. m, 1H, Cy, C*H*_2_*,* alkyl), 1.91 (br. m, 1H, Cy, C*H*_2_ *,* alkyl)^#^, 1.81 (br. s, 1H, Cy, C*H*_2_ *,* alkyl), 1.77 (br. s, 1H, Cy, C*H*_2_ *,* alkyl), 1.61 – 1.53 (br. m, 1H, Cy, C*H*_2_*,* alkyl), 1.47 (s, 12H, Nacnac-C*H*_3_), 1.43 (s, 1H, Cy, C*H*_2_ *,* alkyl), 1.13 (br. d, *J* = 6.3 Hz, 24H, CH(C*H*_3_)_2_), 0.90 (br., 12H, CH(C*H*_3_)_2_)^†^, 0.78 (br., 12H, CH(C*H*_3_)_2_), -0.32 (s, 1H, O*H*).

^13^C NMR (126 MHz, C_6_D_6,_ 300 K) δ 169.4 (N*C*CH_3_), 147.6 (Ar-*C*), 142.8 (Ar-*C*), 142.7 (Ar-*C*), 137.0 (Cy, *C*H, OC*C*H alkene), 126.7 (Cy, *C*H, OCCH*C*H alkene), 125.5 (Ar-*C*), 124.2 (Ar-*C*), 123.8 (Ar-*C*), 95.2 (*C*H), 67.4 (Cy, *C*H, alkyl), 35.3 (Cy, *C*H_2_), 29.0-28.0 (br., Nacnac-C*H*), 25.4 (Cy, *C*H_2_), 25.2 (br., *C*H_3_), 24.8-24.3 (br., Nacnac-*C*H_3_), 22.3 (Cy, *C*H_2_).

*Overlap with C_6_D_6_

^#^Overlap with cyclohexene

^†^Overlap with cyclohexene oxide

Cy = cyclohexenyl

Broad peaks indicate fluxionality in solution.


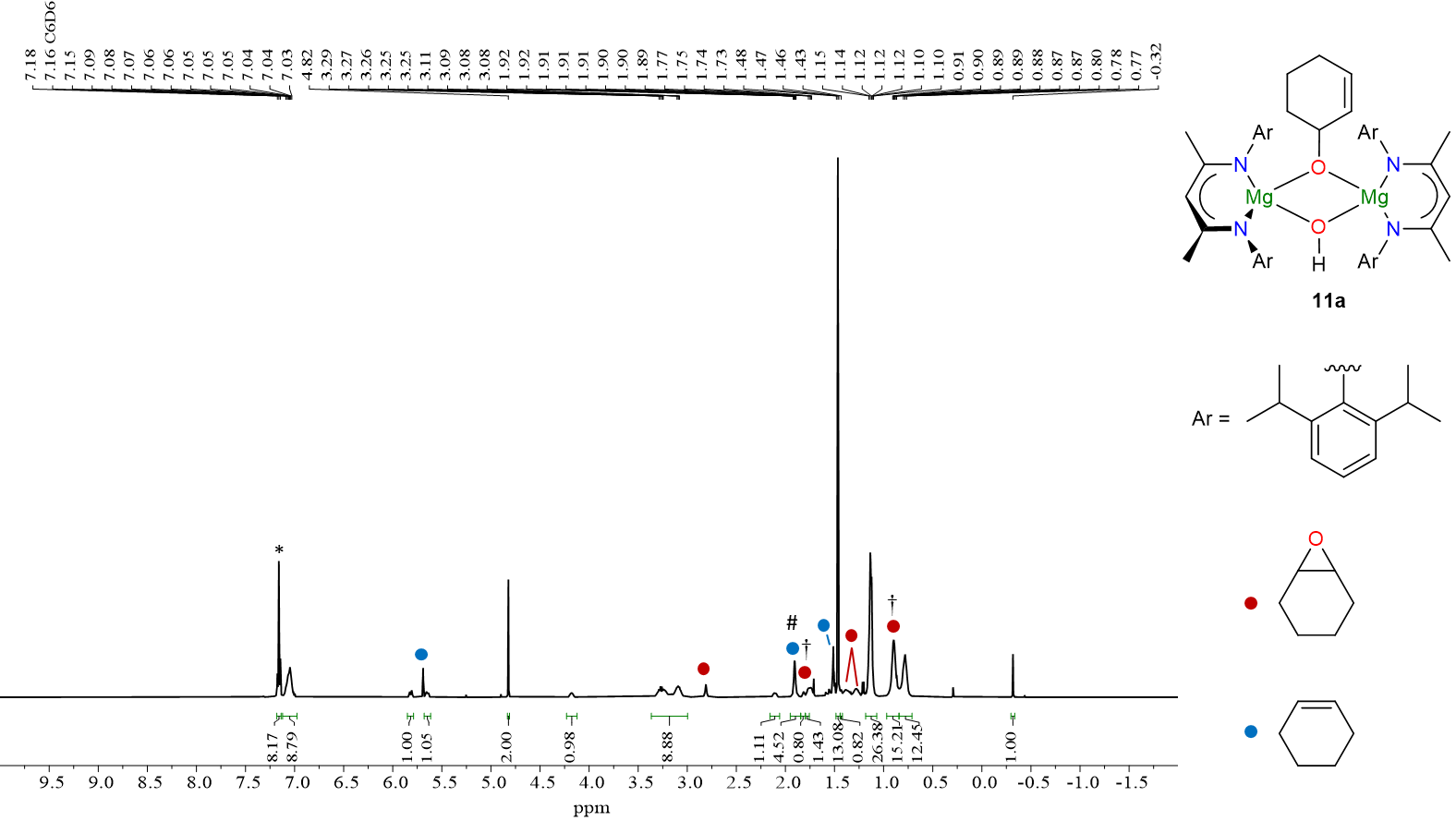


**Figure S45:** ^1^H NMR spectrum of **11a** in C_6_D_6_ recorded at 500 MHz, 300 K. Cylohexene and cyclohexene oxide resonances are identified. *Overlapping peak with C_6_D_6_. ^#^Overlapping peak with cyclohexene. ^†^Overlapping peak with cyclohexene oxide.


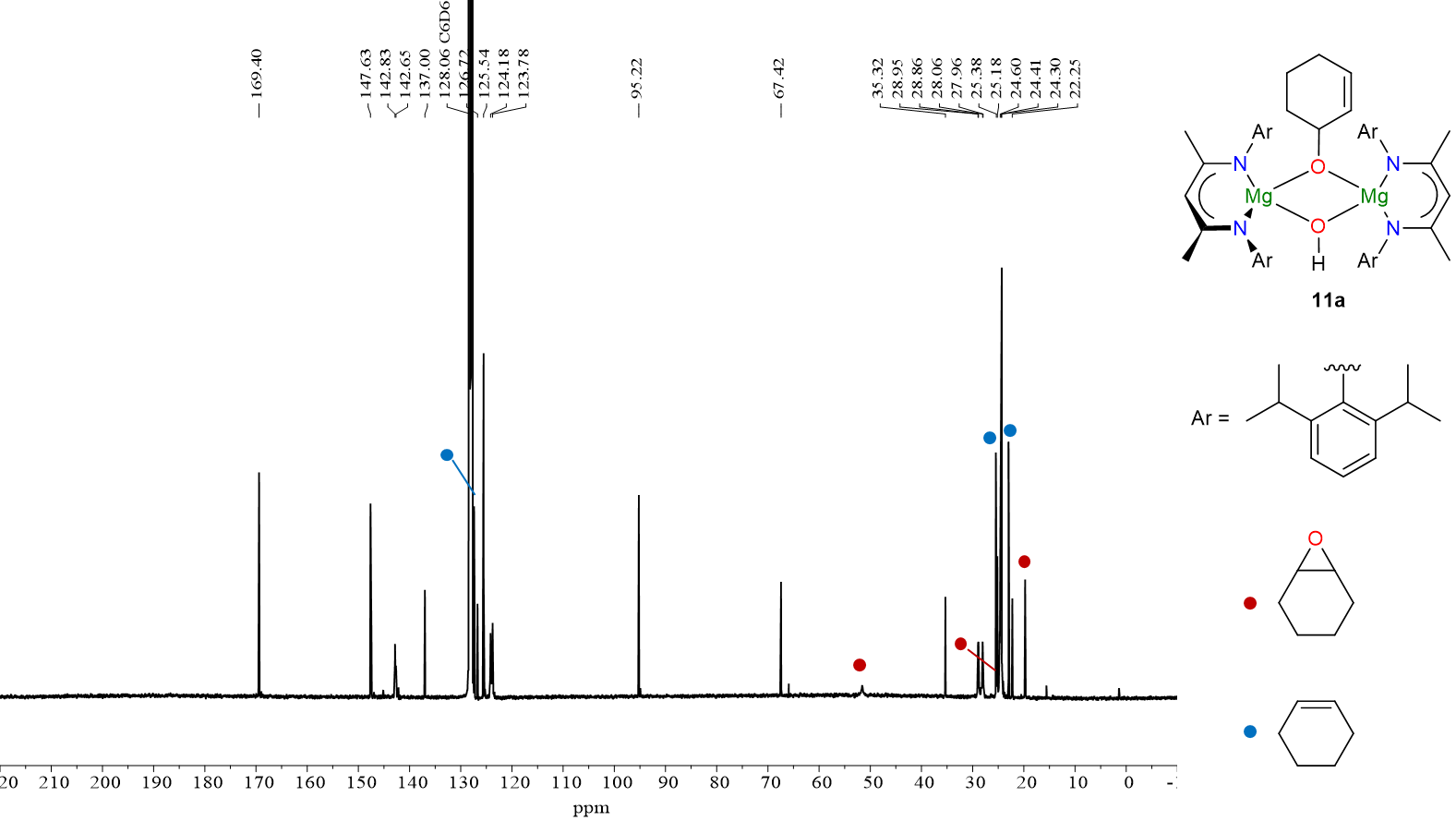


**Figure S46:** ^13^C{^1^H} NMR spectrum of **11a** in C_6_D_6_ recorded at 126 MHz, 300 K. Cyclohexene and cyclohexene oxide resonances are identified.

**Figure S47:** ^1^H-^1^H COSY NMR spectrum of **11a** with cyclohexene and cyclohexene oxide in C_6_D_6_.

**Figure S48:** ^1^H-^13^C HSQC NMR spectrum of **11a** with cyclohexene and cyclohexene oxide in C_6_D_6_.

**Figure S49:** ^1^H-^13^C HMBC NMR spectrum of **11a** with cyclohexene and cyclohexene oxide in C_6_D_6_.

**
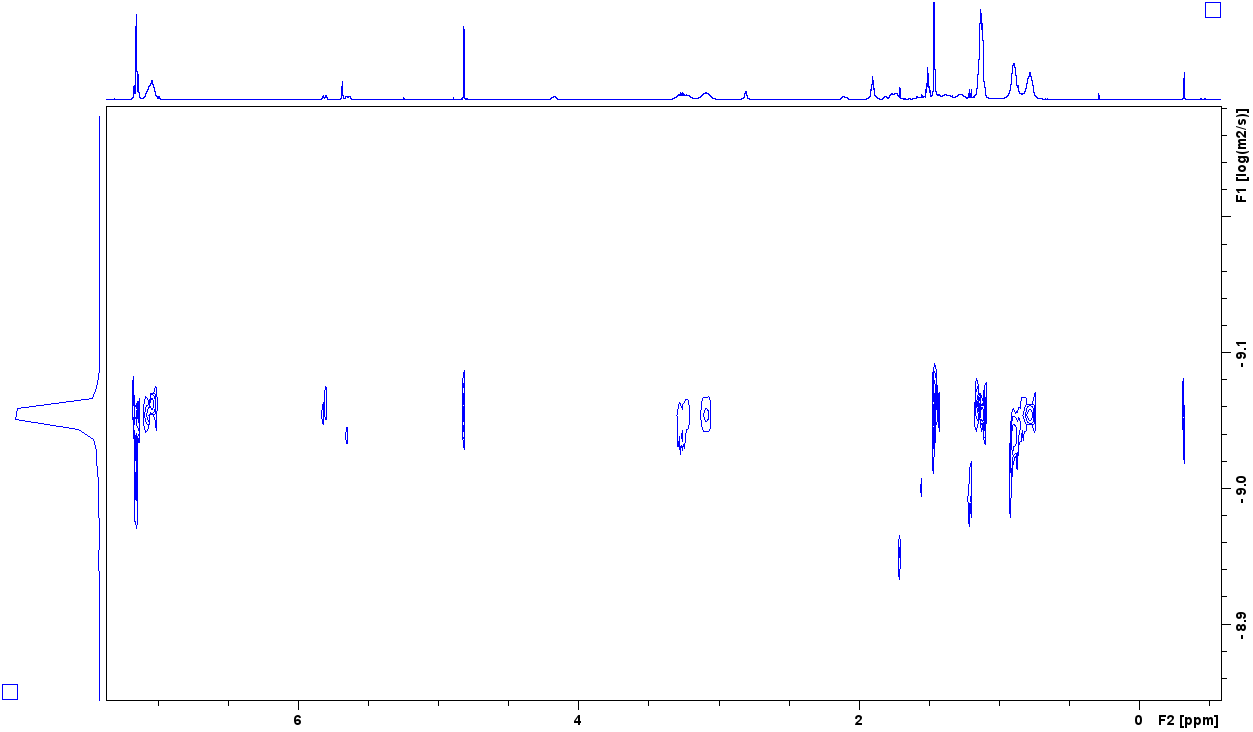
**

**Figure S50:** DOSY NMR spectrum of **11a** with cyclohexene and cyclohexene oxide in C_6_D_6_. All peaks belonging to **11a** appear at the same diffusion coefficient.

### **Synthesis of [^Dipp^NacnacMgS]_2_ (12) in various solvents**

Toluene solvent: [^Dipp^NacnacMg]_2_ (198.3 mg, 224 μmol, 1 eq) was dissolved in toluene (10 mL). The subsequent addition of propylene sulfide (36.8 mg, 496 μmol, 39.2 μL, 2.2 eq) gave a colour change from yellow to colourless, and back to yellow. The reaction mixture was stirred for 1 h, then dried *in vacuo* to give an orange powder. This crude product was suspended in hexane (2 mL), dissolved *via* dropwise addition of toluene (approx. 4 mL) to give a yellow solution, filtered and stored in a freezer (-30 °C), which gave orange crystals of **12** after 16 h (142 mg, 67%). Two sets of crystals were deposited from this reaction. XRD analysis confirmed these two structures to be the solvate free analogue of **12** and the hexane solvate of **12** (Figures S94-S95).

THF: The related THF adduct [^Dipp^NacnacMgS•THF]_2_ of **12** (**12**•THF_2_) has been previously reported, formed *via* the reaction of an azobenzenyl anion radical complex of magnesium [^Dipp^NacnacMg(PhNNPh)•THF] with elemental sulfur.^[29]^

Et_2_O: [^Dipp^NacnacMg]_2_ (10.5 mg, 11.9 μmol, 1 eq) was partially dissolved in Et_2_O (1 mL) to give a pale yellow suspension. Propylene sulfide (1.76 mg, 23.8 μmol, 1.87 μL, 2 eq) was added and the reaction mixed, which formed a colourless solution. The reaction mixture was kept at room temperature overnight, which yielded pale yellow crystals, that were characterised by XRD to be a disordered mix of products, where **12**•(Et_2_O)_2_ was formed as the minor species (16%, Figure S104) and **13**•(Et_2_O)_2_ (84%, Figure S105) was present as the major species.

Complex **12**: ^1^H NMR (500 MHz, C_6_D_6_, 300 K) δ 7.15 – 7.13 (m, 4H, Ar-*H*)*, 7.05 (d, *J* = 7.6 Hz, 8H, Ar-*H*), 4.88 (s, 2H, Nacnac-C*H*), 3.13 (hept, *J* = 6.8 Hz, 8H, C*H*(CH_3_)_2_), 1.64 (s, 12H, Nacnac-C*H*_3_), 1.14 (d, *J* = 6.8 Hz, 24H, CH(C*H*_3_)_2_), 1.06 (d, *J* = 6.8 Hz, 24H, CH(C*H*_3_)_2_).

^13^C NMR (126 MHz, C_6_D_6_, 300 K) δ 170.4 (N*C*CH_3_), 143.8 (Ar-*C*), 142.3 (Ar-*C*), 125.7 (Ar-*C*), 124.0 (Ar-*C*), 94.8 (Nacnac-*C*H), 28.6 (*C*H_2_(CH_3_)_2_), 24.9 (Nacnac-*C*H_3_), 24.4 (CH_2_(*C*H_3_)_2_), 24.0 (CH_2_(*C*H_3_)_2_).

*Overlap with C_6_D_6_

Elemental analysis calcd for C_58_H_82_Mg_2_N_4_O_2_S_2_: C 73.48, H 9.16, N 5.91, S 6.76, found: C 75.23, H 9.11, N 5.91, S 6.50.


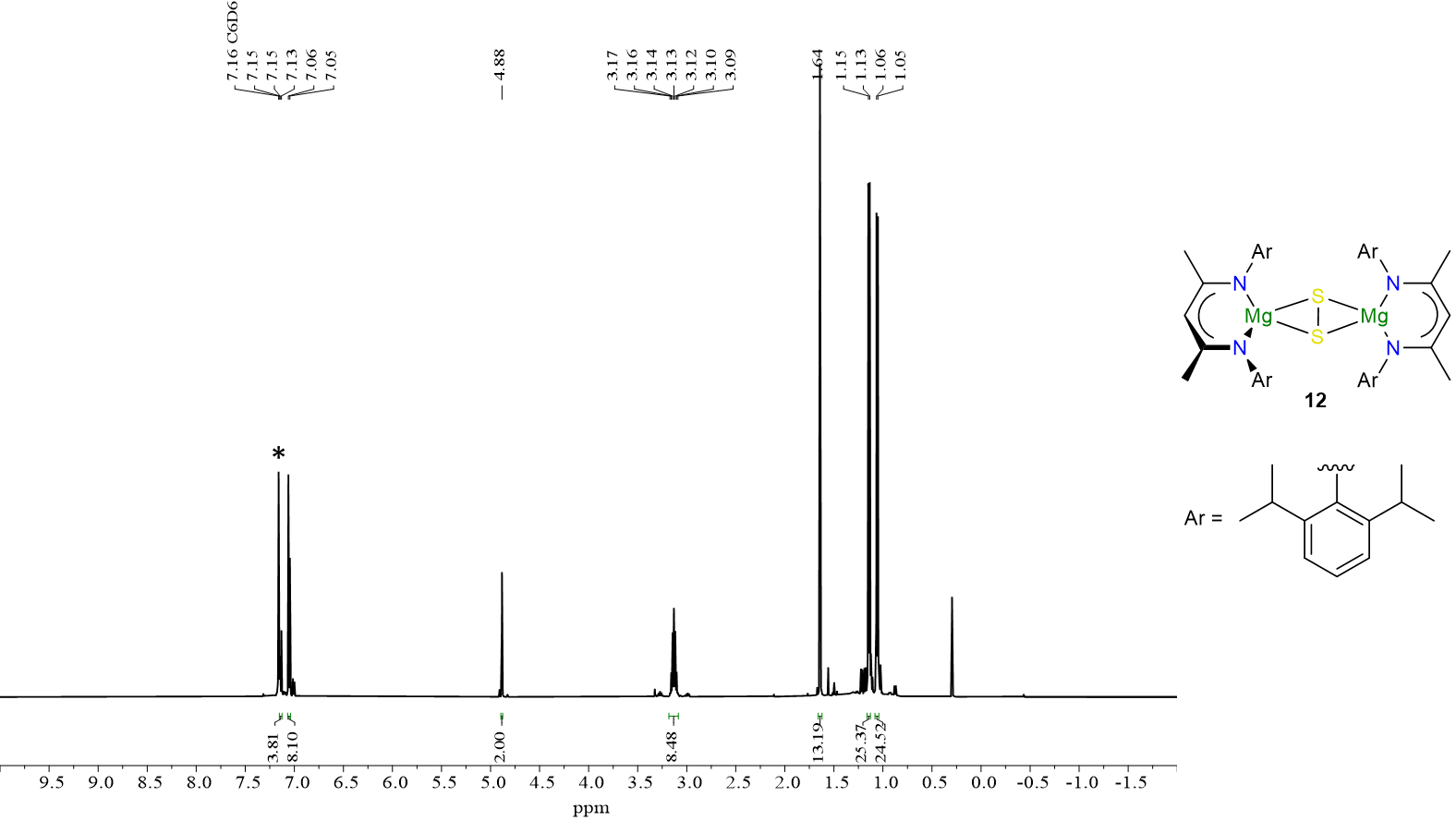


**Figure S51:** ^1^H NMR spectrum of **12** in C_6_D_6_ recorded at 500 MHz, 300 K. Overlapping peak with C_6_D_6_.


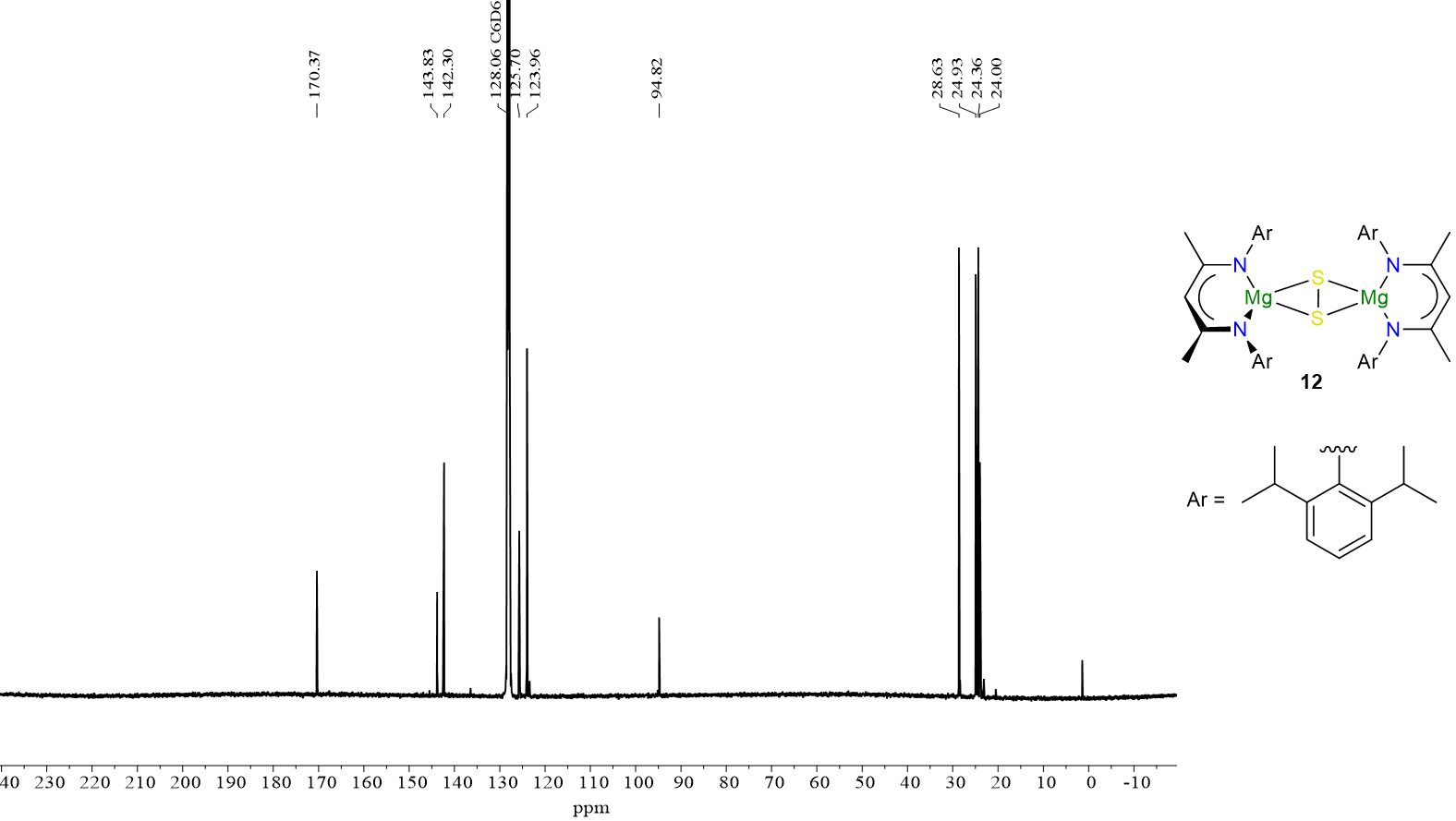


**Figure S52:** ^13^C{^1^H} NMR spectrum of **12** in C_6_D_6_ recorded at 126 MHz, 300 K.

**Figure S53:** ^1^H-^1^H COSY NMR spectrum of **12** in C_6_D_6_.

**Figure S54:** ^1^H-^13^C HSQC NMR spectrum of **12** in C_6_D_6_.

**Figure S55:** ^1^H-^13^C HMBC NMR spectrum of **12** in C_6_D_6_.

**
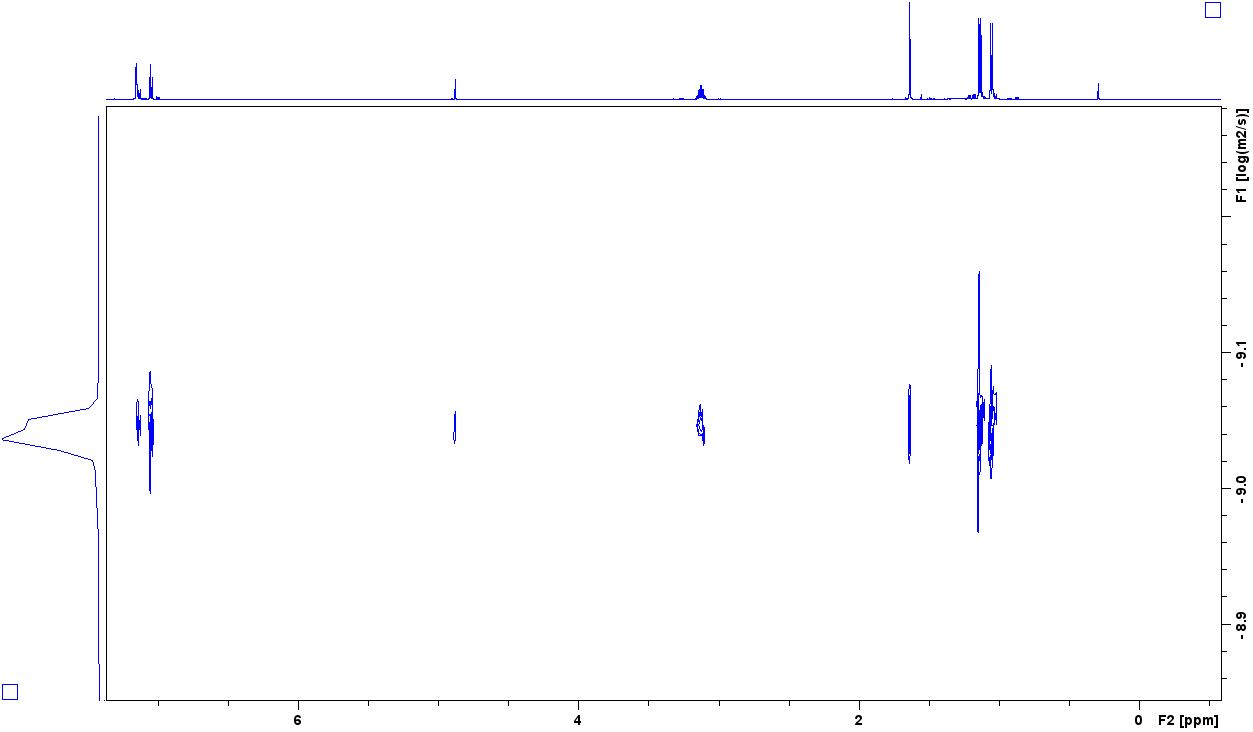
**

**Figure S56:** DOSY NMR spectrum of **12** in C_6_D_6_. All peaks belonging to **12** appear at the same diffusion coefficient.

### **Synthesis of [(^Dipp^NacnacMg)_2_S•THF_2_] (13•THF_2_)**

[^Dipp^NacnacMg]_2_ (98.5 mg, 111 μmol, 1 eq) was dissolved in THF (5 mL) and propylene sulfide (9.9 mg, 133 μmol, 10.5 μL, 1.2 eq) was subsequently added. This gave a colour change from yellow to colourless. The reaction mixture was stirred for 30 mins, then dried *in vacuo* to give **13** (74.9 mg, 65%).

Crystals suitable for single crystal XRD analysis were obtained by suspending **13** (25 mg) in hexane (2 mL), with dropwise addition of toluene (approx. 0.2 mL) until a colourless solution formed. The solution was subsequently filtered and stored in a freezer (-30 ⁰C), which deposited colourless crystals of **13** after 3 days (Figure S99).

Complex **13**•THF_2_: ^1^H NMR (500 MHz, C_6_D_6_, 300 K) δ 7.13 – 7.08 (m, 12H, Ar-*H*), 4.74 (s, 2H, Nacnac-C*H*), 3.57 – 3.49 (m, 8H, THF, OC*H*_2_CH_2_), 3.18 (br., 8H, C*H*(CH_3_)_2_), 1.64 (s, 12H, Nacnac-C*H*_3_), 1.47 – 1.39 (m, 8H, THF, OCH_2_C*H*_2_), 1.24 (d, *J* = 6.9 Hz, 24H, CH(C*H*_3_)_2_), 1.14 (d, *J* = 6.9 Hz, 24H, CH(C*H*_3_)_2_).

^13^C NMR (126 MHz, C_6_D_6_, 300 K) δ 167.7 (N*C*CH_3_), 146.5 (Ar-*C*), 142.7 (Ar-*C*), 124.5 (Ar-*C*), 123.4 (Ar-*C*), 94.0 (Nacnac-*C*H), 69.9 (THF, O*C*H_2_CH_2_), 28.2 (*C*H_2_(CH_3_)_2­_), 25.6 (CH(C*H*_3_)_2_), 25.5 (THF, OCH_2_*C*H_2_), 24.6 (CH(C*H*_3_)_2_), 24.4 (Nacnac-*C*H_3_).

Elemental analysis calcd for C_66_H_98_Mg_2_N_4_O_2_S: C 74.77, H 9.32, N 5.28, S 3.02, found: C 77.57, H 9.80, N 5.55, S 1.91.


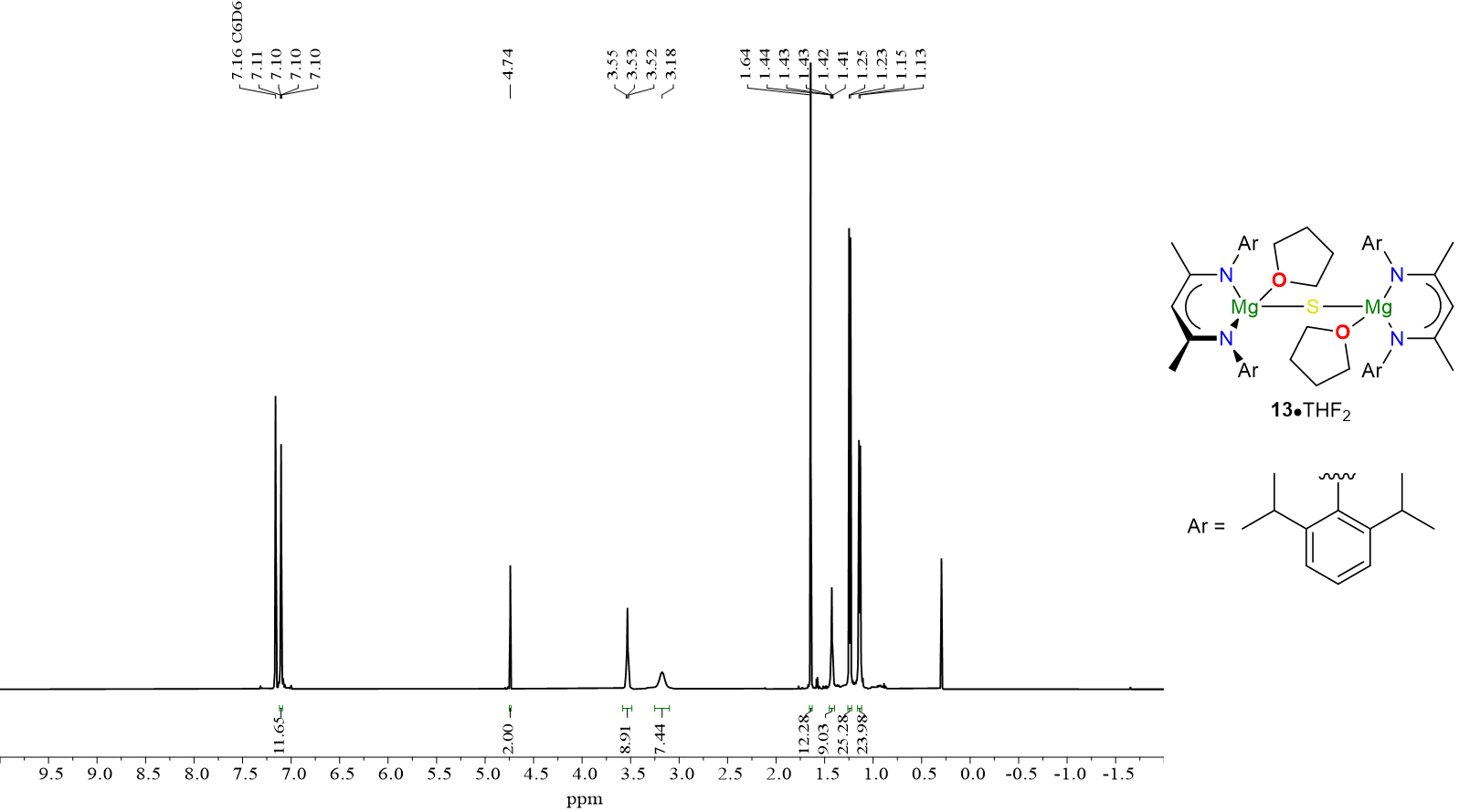


**Figure S57:** ^1^H NMR spectrum of **13**•THF_2_ in C_6_D_6_ recorded at 500 MHz, 300 K.


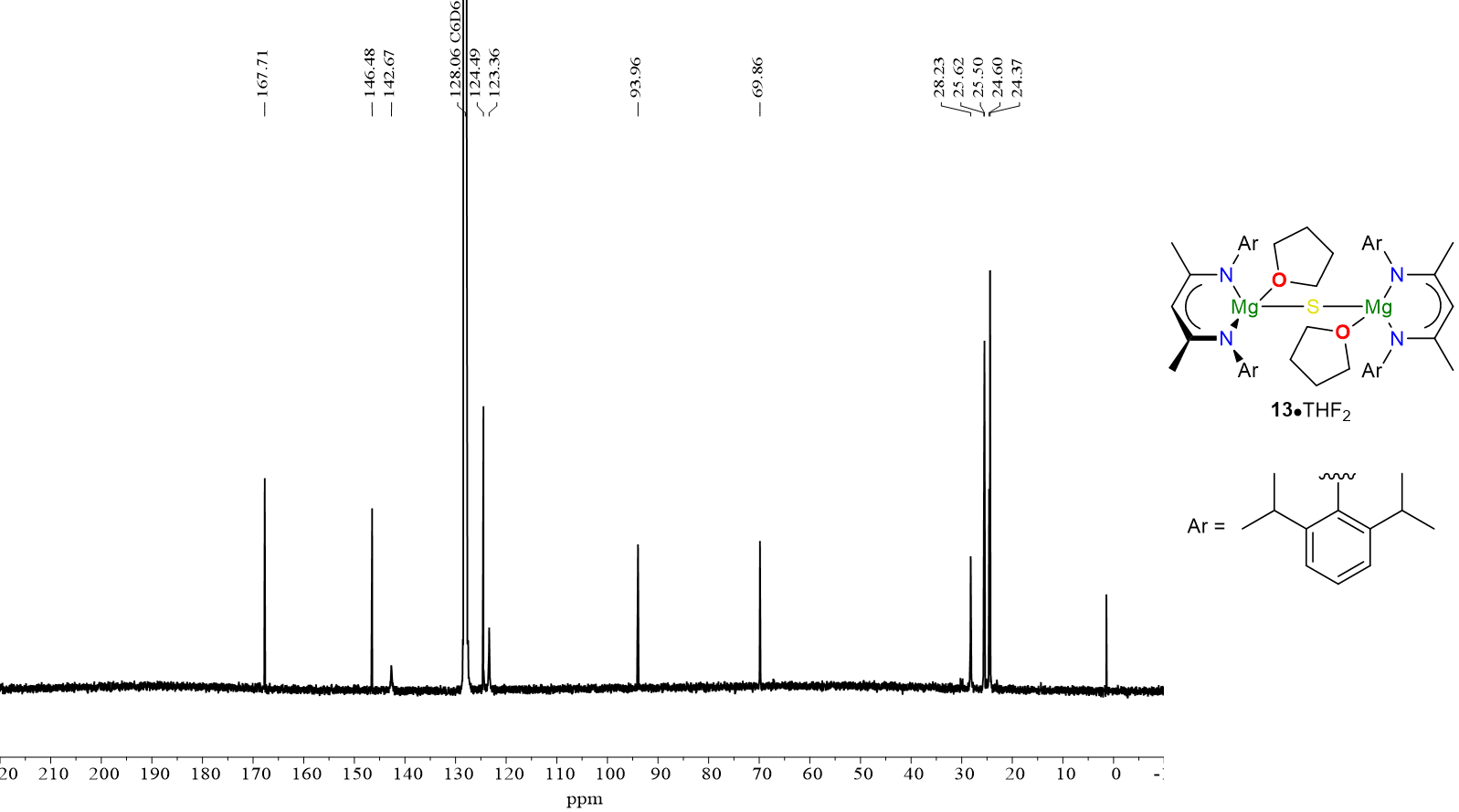


**Figure S58:** ^13^C{^1^H} NMR spectrum of **13**•THF_2_ in C_6_D_6_ recorded at 126 MHz, 300 K.

**Figure S59:** ^1^H-^1^H COSY NMR spectrum of **13**•THF_2_ in C_6_D_6_.

**Figure S60:** ^1^H-^13^C HSQC NMR spectrum of **13**•THF_2_ in C_6_D_6_.

**Figure S61:** ^1^H-^13^C HMBC NMR spectrum of **13**•THF_2_ in C_6_D_6_.

**
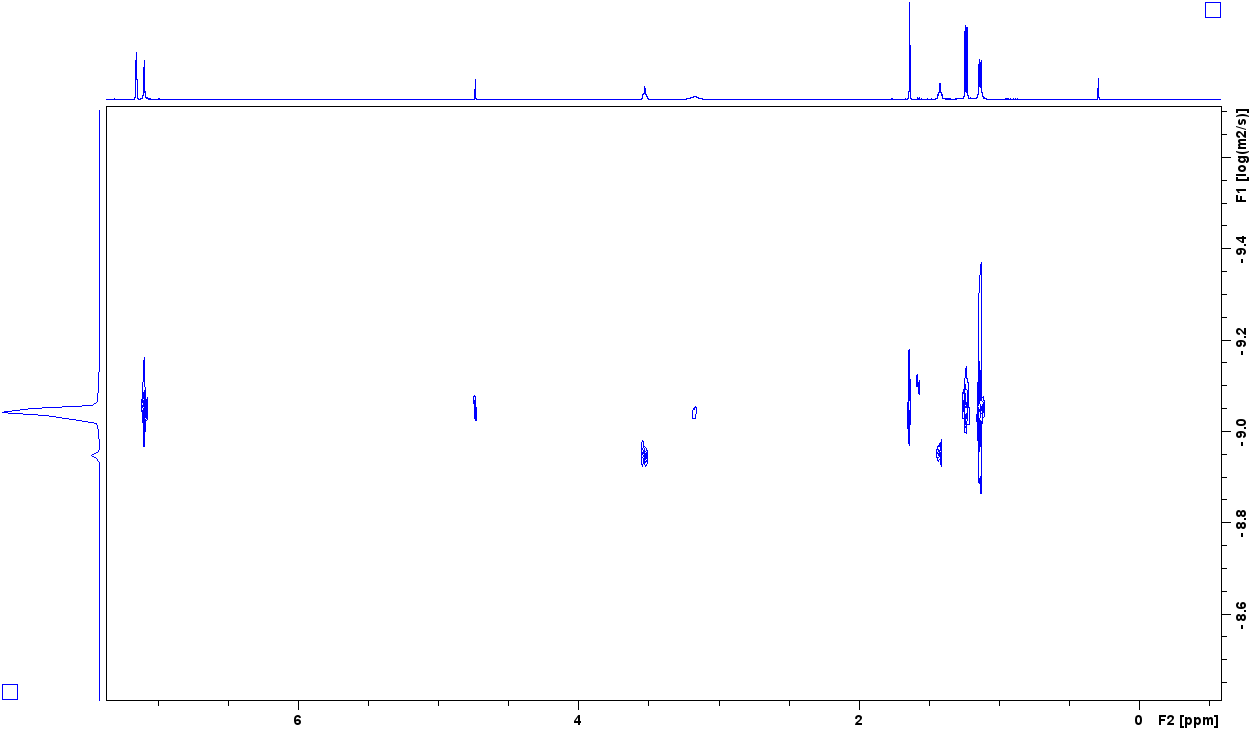
**

**Figure S62:** DOSY NMR spectrum of **13**•THF_2_ in C_6_D_6_. All peaks belonging to **13** appear at the same diffusion coefficient. The lower logD value of THF indicates that there is some lability of the Mg-THF adduct in C_6_D_6_ solvent.

### **Synthesis of [(^Dipp^NacnacMg)_2_S•(OEt_2_)_2_] (13•(Et_2_O)_2_)**

[^Dipp^NacnacMg]_2_ (12.4 mg, 140 μmol, 1 eq) was partially dissolved and suspended in Et_2_O (1 mL) to give a pale yellow suspension. Propylene sulfide (2.08 mg, 281 μmol, 2.21 μL, 2 eq) was subsequently added and the reaction was mixed using a syringe (approx. 1 min) to give a clear colourless solution, which was transferred to a J Youngs tap NMR tube. The reaction mixture was dried *in vacuo* and dissolved in C_6_D_6_ to give **13**•(Et_2_O)_2_.

Complex **13**•(Et_2_O)_2_: ^1^H NMR (500 MHz, C_6_D_6_, 300 K) δ 7.18 – 7.14 (m, 4H, Ar-*H*)*, 7.14 – 7.06 (m, 8H, Ar-*H*), 4.78 (s, 2H, Nacnac-C*H*), 3.50 (q, *J* = 6.9 Hz, 8H, Et_2_O, OC*H*_2_CH_3_), 3.15 (hept, *J* = 6.9 Hz, 8H, C*H*(CH_3_)_2_), 1.57 (s, 12H, Nacnac-C*H*_3_), 1.20 (d, *J* = 6.9 Hz, 24H, CH(C*H*_3_)_2_), 1.11 (d, *J* = 6.9 Hz, 24H, CH(C*H*_3_)_2_), 0.99 (t, *J* = 6.9 Hz, 12H, Et_2_O, OCH_2_C*H*_3_).
^13^C NMR (126 MHz, C_6_D_6_, 300 K) δ 169.2 (N*C*CH_3_), 145.3 (Ar-*C*), 142.4 (Ar-*C*), 125.2 (Ar-*C*), 123.8 (Ar-*C*), 94.9 (Nacnac-*C*H), 65.4 (Et_2_O, O*C*H_2_CH_3_), 28.3 (*C*H_2_(CH_3_)_2­_), 25.6 (CH(C*H*_3_)_2_), 24.3 (CH(C*H*_3_)_2_), 24.2 (CH(C*H*_3_)_2_), 14.7 (Et_2_O, OCH_2_*C*H_3_).

*Overlap with C_6_D_6_


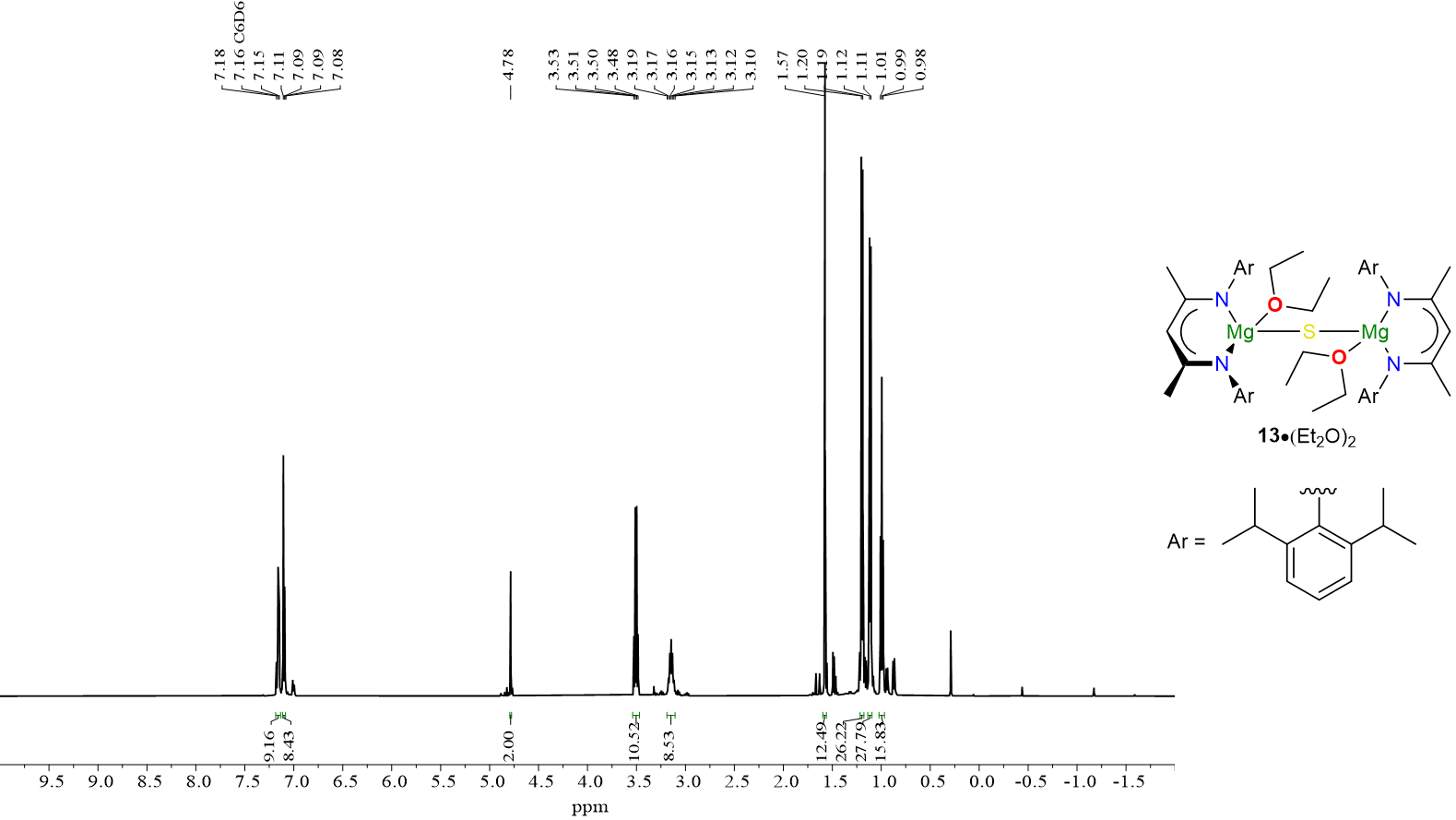


**Figure S63:** ^1^H NMR spectrum of **13**•(Et_2_O)_2_ in C_6_D_6_ recorded at 500 MHz, 300 K.


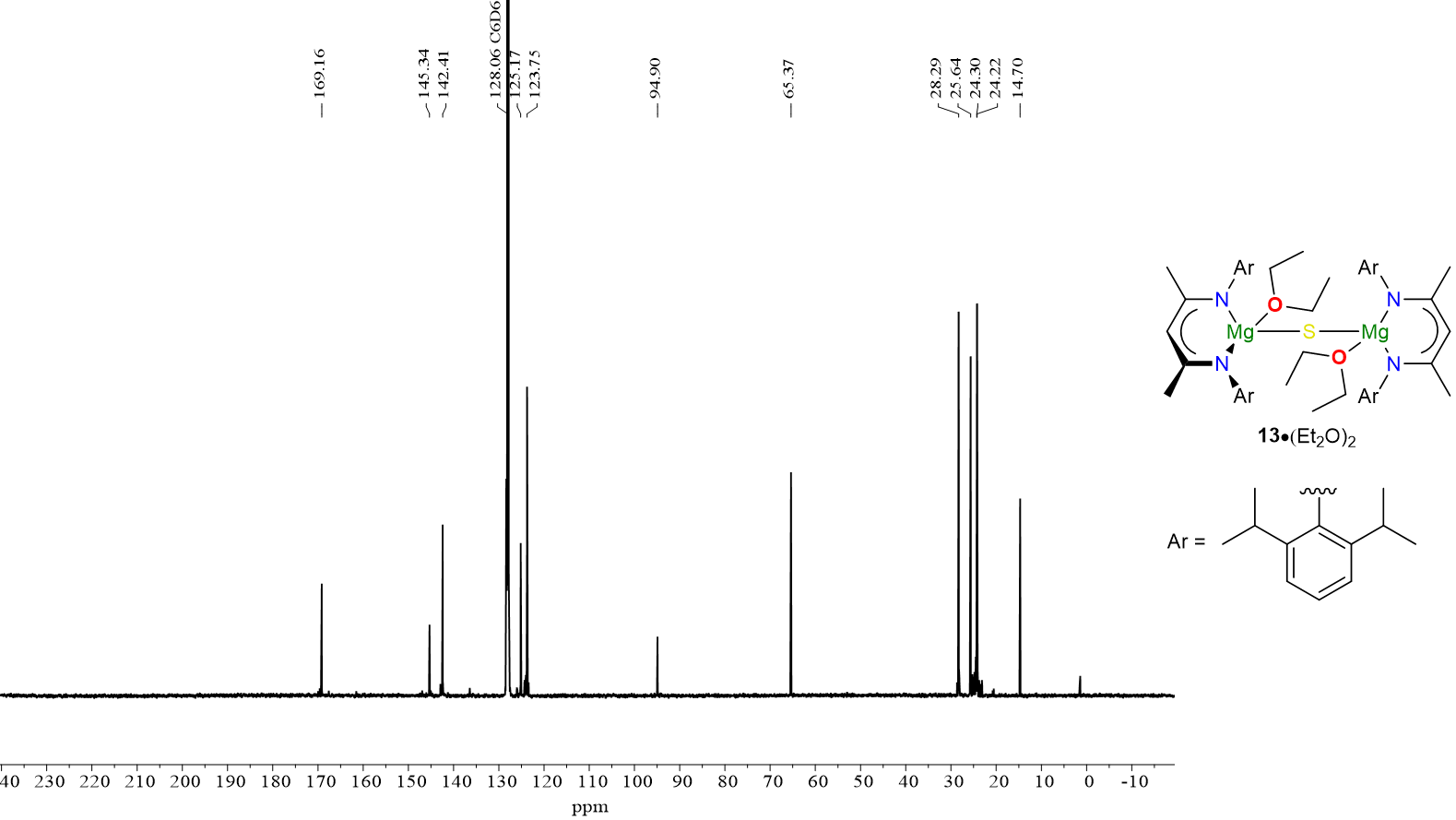


**Figure S64:** ^13^C{^1^H} NMR spectrum of **13**•(Et_2_O)_2_ in C_6_D_6_ recorded at 126 MHz, 300 K.

**Figure S65:** ^1^H-^1^H COSY NMR spectrum of **13**•(Et_2_O)_2_ in C_6_D_6_.

**Figure S66:** ^1^H-^13^C HSQC NMR spectrum of **13**•(Et_2_O)_2_ in C_6_D_6_.

**Figure S67:** ^1^H-^13^C HMBC NMR spectrum of **13**•(Et_2_O)_2_ in C_6_D_6_.

**
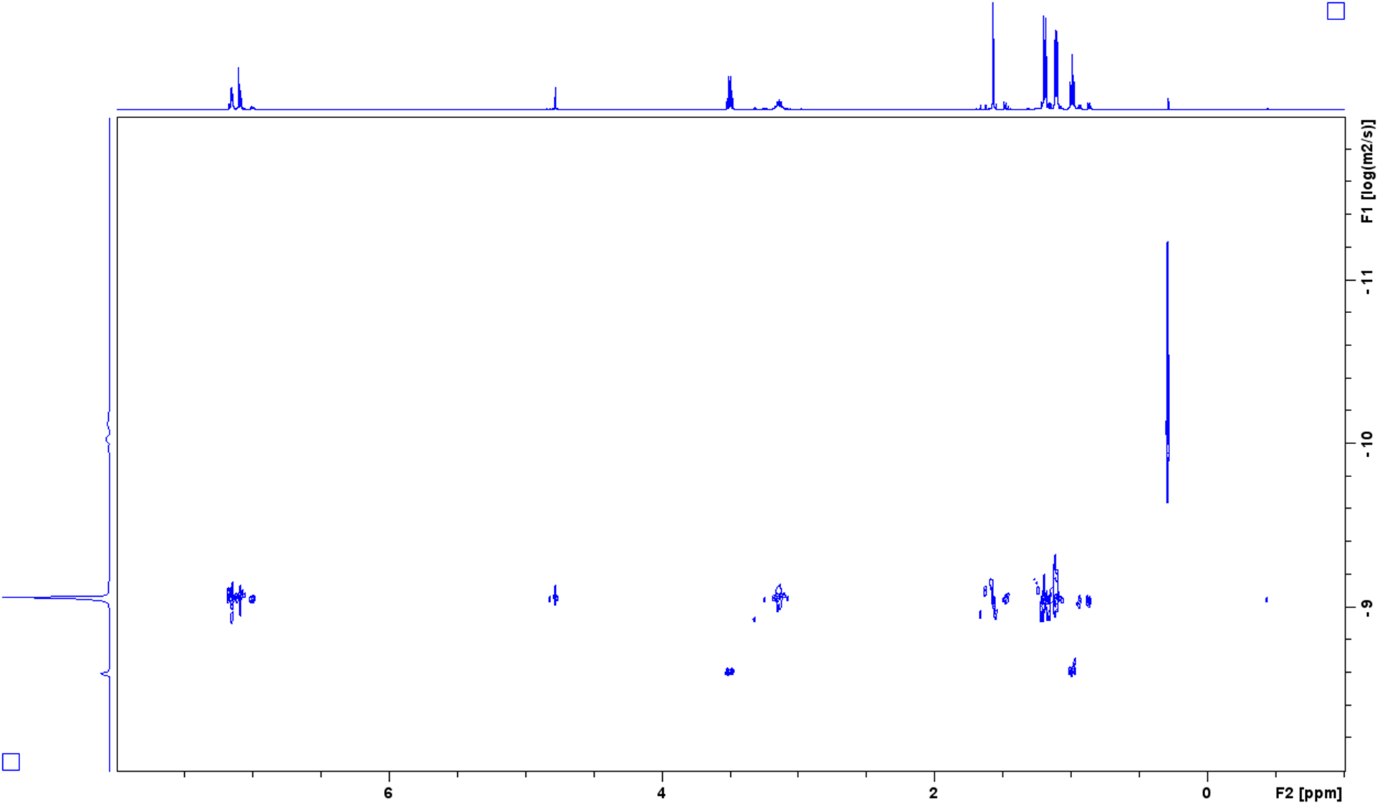
**

**Figure S68:** DOSY NMR spectrum of **13**•(Et_2_O)_2_ in C_6_D_6_. All peaks belonging to **13** appear at the same diffusion coefficient. The lower logD value of Et_2_O indicates that there is some lability of the Mg-OEt_2_ adduct in C_6_D_6_ solvent.

### **Synthesis of [^Dipp^NacnacMg(SH)(OCH_2_CHCH_2_)Mg^Dipp^Nacnac] (14)**

[^Dipp^NacnacMg]_2_ (10.3 mg, 11.6 μmol, 1 eq) was partially dissolved to form a pale yellow suspension in Et_2_O (1 mL). Propylene sulfide (1.3 mg, 17.5 μmol, 1.39 μL, 1.5 eq) was added and the reaction was mixed using a syringe (for approx. 1 min). This gave a colourless solution that was transferred to a J Youngs tap NMR tube. The reaction mixture was dried *in vacuo* and the product, **13**•(Et_2_O)_2_, was dissolved in C_6_D_6_ (0.6 mL). Propylene oxide (0.74 mg, 12.8 µmol, 1.5 eq, 7.4 uL of 100 mg mL^-1^ solution in C_6_D_6_) was subsequently added, and the J Youngs NMR tube was shaken and kept at room temperature overnight. The reaction mixture was then dried *in vacuo* and dissolved in C_6_D_6_ solvent to give mixed-chalcogen product **14**. The rection mixture was dried *in vacuo*, redissolved in *d*_8_-toluene (0.5 mL), layered with hexane (0.2 mL) and stored at -30 °C, yielding colourless crystals of **14** suitable for single crystal XRD over three days (Figure S107).

Complex **14**: ^1^H NMR (500 MHz, C_6_D_6_, 300 K) δ 7.14 (d, *J* = 7.6 Hz, 4H, Ar-*H*), 7.05 (ddd, *J* = 12.4, 7.6, 1.6 Hz, 8H, Ar-*H*), 6.09 (ddt, *J* = 16.4, 10.2, 6.1 Hz, 1H, OCH_2_C*H*CH_2_), 5.13 – 5.03 (m, 2H, OCH_2_CHC*H*_2_), 4.78 (s, 2H, Nacnac-C*H*), 4.31 (dt, *J* = 6.3, 1.4 Hz, 2H, OC*H*_2_CHCH_2_), 3.32 (hept, *J* = 7.2 Hz, 4H, C*H*(CH_3_)_2_), 3.21 (hept, *J* = 6.8 Hz, 4H, C*H*(CH_3_)_2_), 1.46 (s, 12H, Nacnac-C*H*_3_), 1.12 (dd, *J* = 8.8, 6.8 Hz, 24H, CH(C*H*_3_)_2_), 0.91 (br. m, 24H, CH(C*H*_3_)_2_), -1.55 (s, 1H, S*H*).

^13^C NMR (126 MHz, C_6_D_6,_ 300 K) δ 169.6 (N*C*CH_3_), 146.4 (Ar-*C*), 143.3 (Ar-*C*), 142.6 (Ar-*C*) 140.3 (OCH_2_*C*HCH_2_), 125.9 (Ar-*C*), 124.4 (Ar-*C*), 123.8 (Ar-*C*), 115.2 (OCH_2_CH*C*H_2_), 95.5 (Nacnac-*C*H), 66.5 (O*C*H_2_CHCH_2_), 28.5 (*C*H_2_(CH_3_)_2_), 28.2 (*C*H_2_(CH_3_)_2_), 25.0 (CH_2_(*C*H_3_)_2_), 24.8 (CH_2_(*C*H_3_)_2_), 24.7 (Nacnac-*C*H_3_), 24.4 (CH_2_(*C*H_3_)_2_), 24.2 (CH_2_(*C*H_3_)_2_).

*Overlap with C_6_D_6_


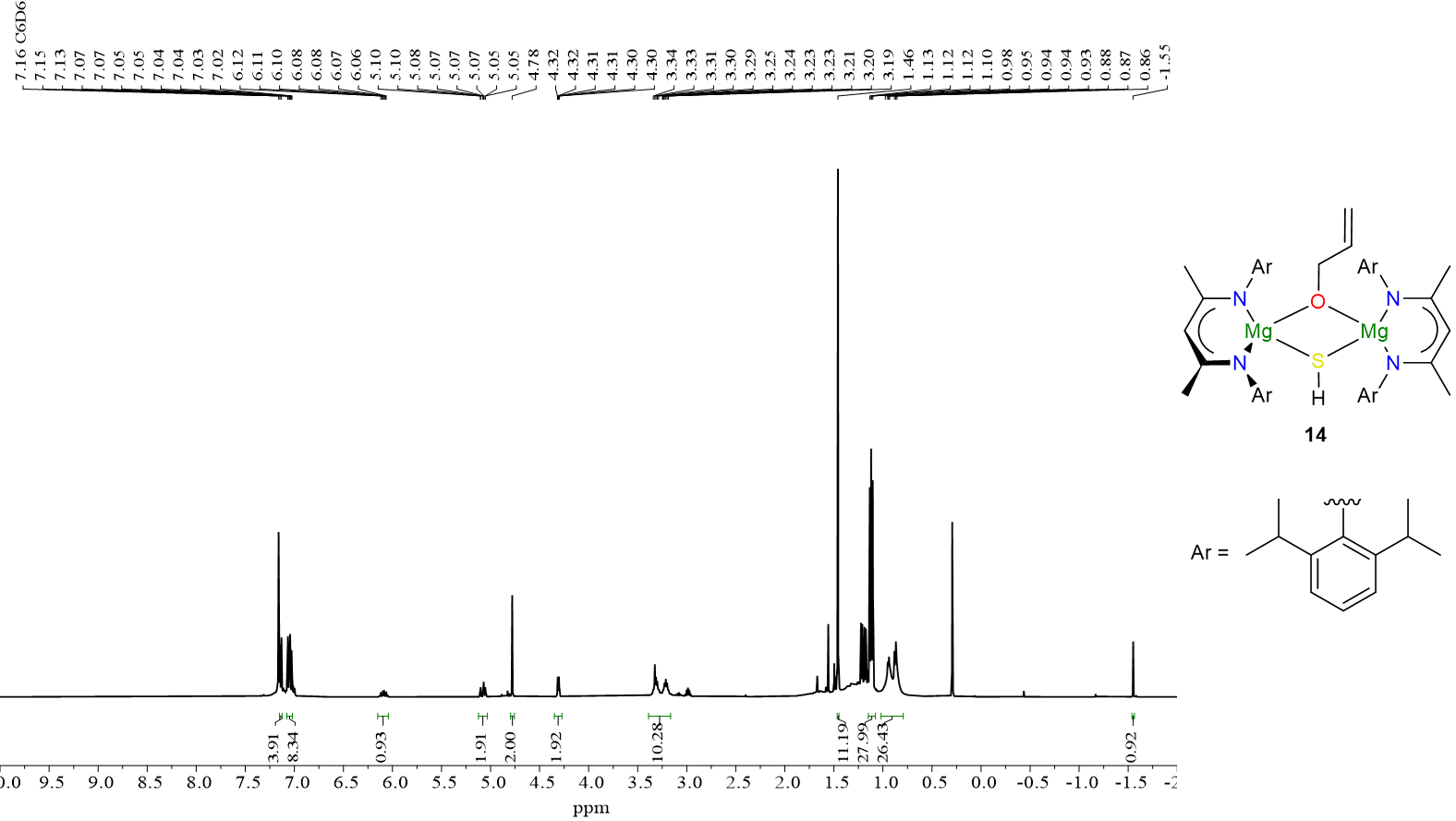


**Figure S69:** ^1^H NMR spectrum of **14** in C_6_D_6_ recorded at 500 MHz, 300 K.


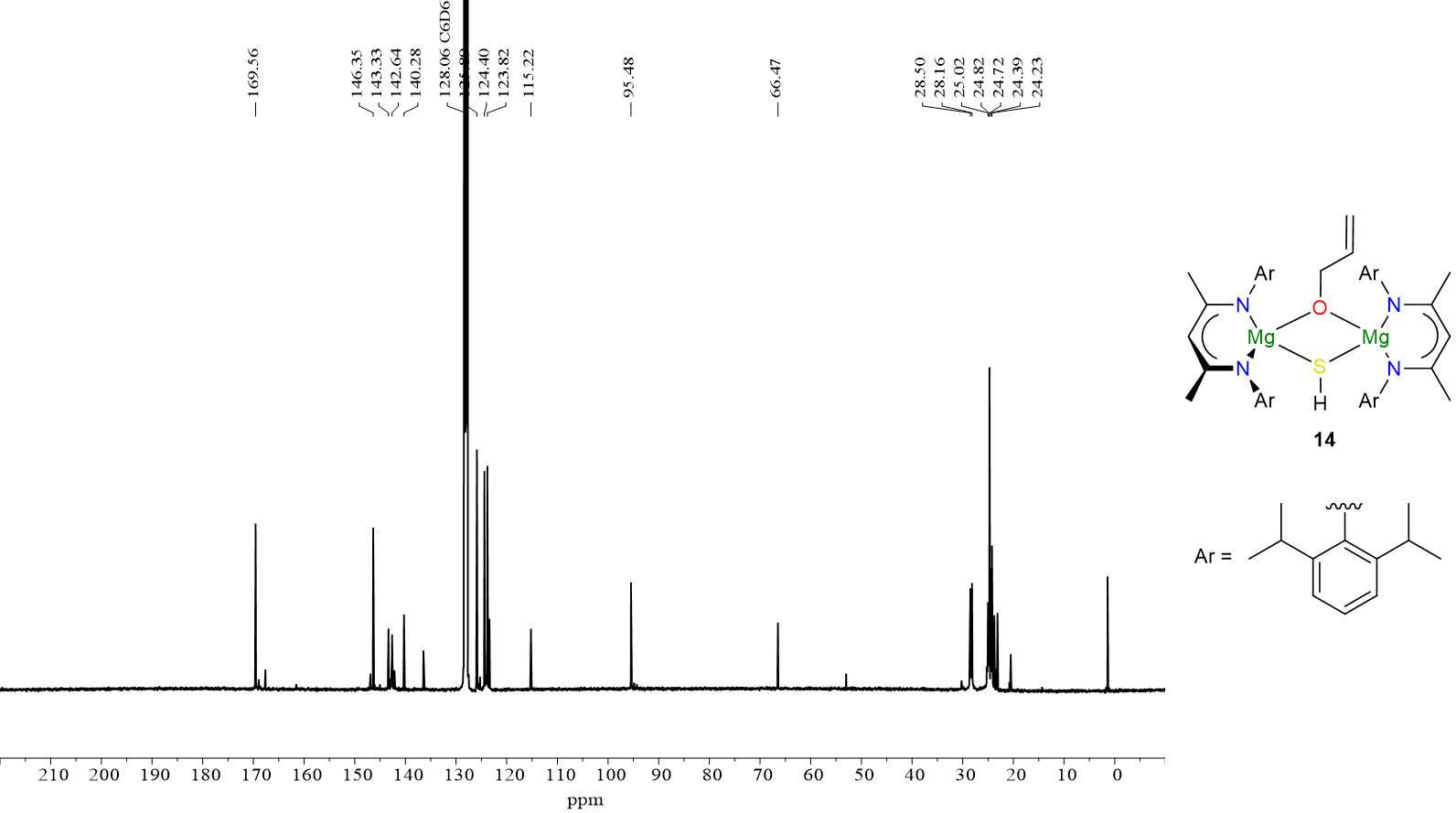


**Figure S70:** ^13^C{^1^H} NMR spectrum of **14** in C_6_D_6_ recorded at 126 MHz, 300 K.

**Figure S71:** ^1^H-^1^H COSY NMR spectrum of **14** in C_6_D_6_.

**Figure S72:** ^1^H-^13^C HSQC NMR spectrum of **14** in C_6_D_6_.

**Figure S73:** ^1^H-^13^C HMBC NMR spectrum of **14** in C_6_D_6_.

**
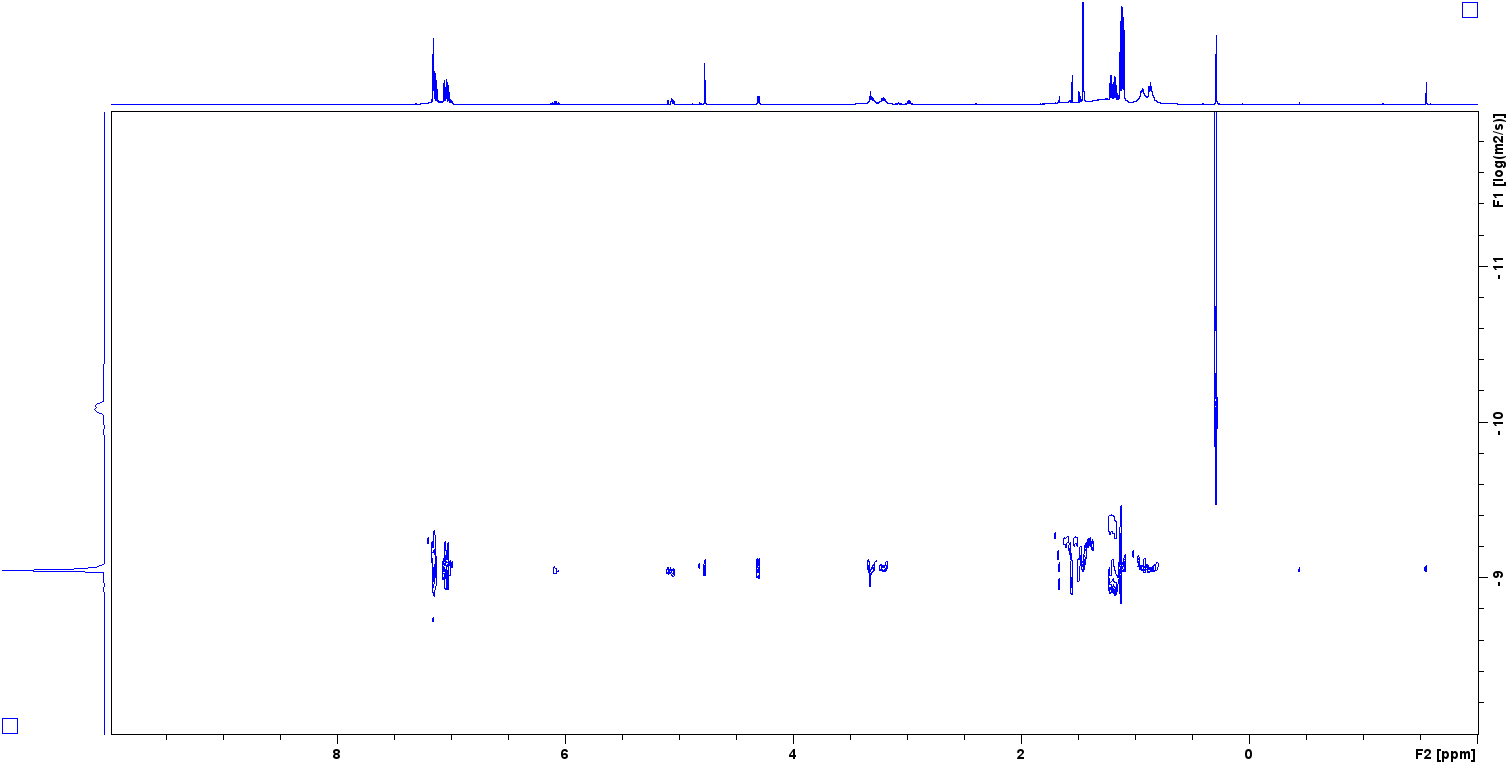
**

**Figure S74**: DOSY NMR spectrum of **14** in C_6_D_6_. All peaks belonging to **14** appear at the same diffusion coefficient.

## **NMR Spectroscopic Investigations**

### **Comparison of Complexes 4 and 4D**

Comparison of the ^1^H and ^2^D NMR spectra for complexes **4** and **4D** confirms that propylene oxide is deprotonated to generate the bridging hydroxide and alkoxide units of [^Dipp^NacnacMg(OH)(OCH_2_CHCH_2_)Mg^Dipp^Nacnac] **4** or [^Dipp^NacnacMg(OD)(OCD_2_CDCD_2_)Mg^Dipp^Nacnac] **4D**, respectively.

Crucial differences were observed depending on whether proteo-PO or deutero-PO (*d*_6_-PO) was used; the reactions were otherwise identical. Key features of the spectra include:

- The loss of the -O*H* resonance in the ^1^H NMR spectrum, and the appearance of the -O*D* resonance in the ^2^D NMR spectrum when deutero-PO was used instead of proteo-PO (*i.e.* the synthesis of **4D**).
- The loss of the propene and alkoxide resonances of **4** in the ^1^H NMR spectrum, and the appearance of propene and alkoxide resonances of **4D** in the 2D NMR spectrum when using deutero-PO instead of proteo-PO.
- Apart from the -O*H*/-O*D* resonances and the alkoxide resonances, all other ligand resonances remain identical in **4** and **4D**.


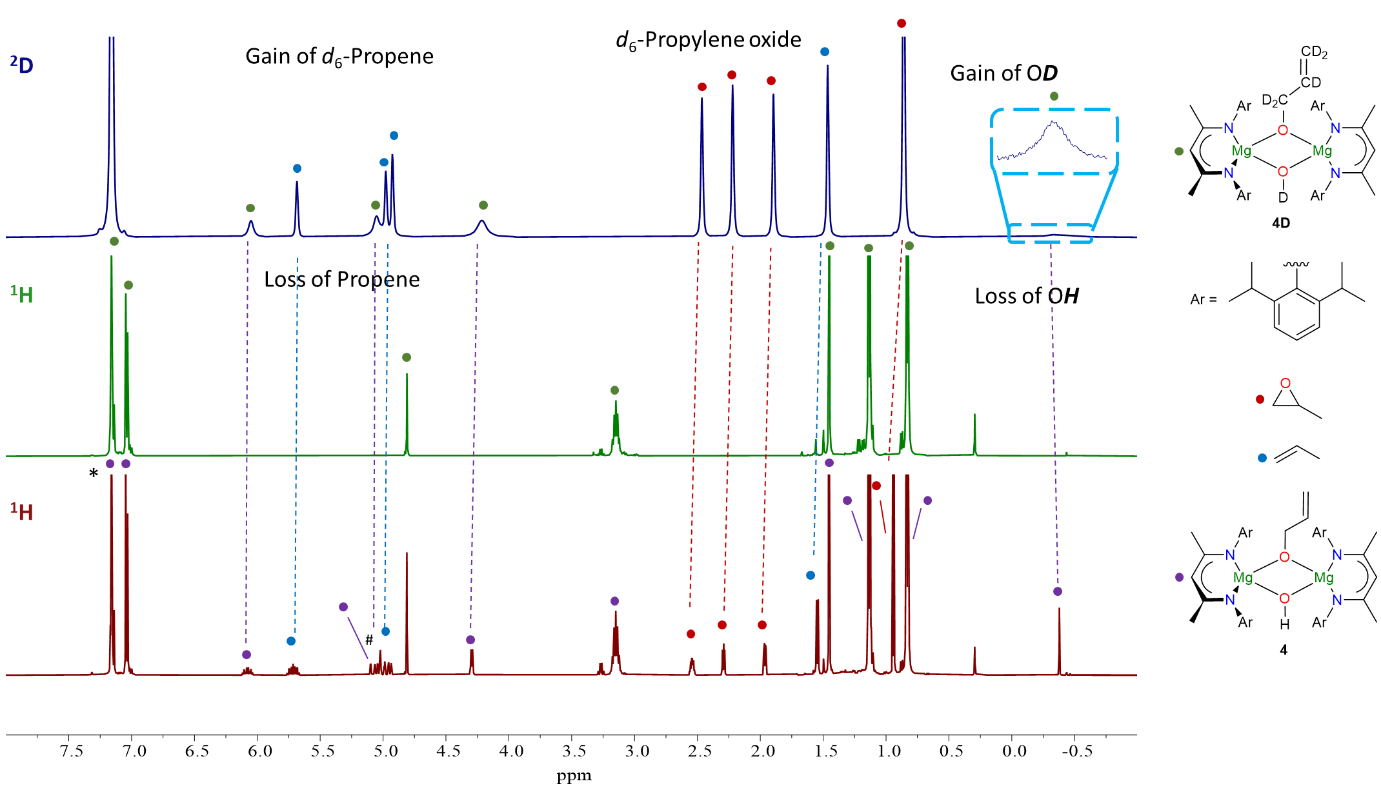


**Figure S75:** Stacked ^1^H NMR spectra of **4** (bottom, maroon, C_6_D_6_), **4D** (middle, green, C_6_D_6_) and ^2^D NMR spectrum of **4D** (top, blue, C_6_H_6_ with 1 µL C_6_D_6_ spike).

### **Synthesis of Complex 4 from [(^Dipp^NacnacMg)_2_O] (8)**

[(^Dipp^NacnacMg)_2_O] was synthesised from [^Dipp^NacnacMg]_2_ **1** and N_2_O, according to literature protocols.^[7]^

In a glovebox, [(^Dipp^NacnacMg)_2_O] (9.7 mg, 10.7 μmol, 1 eq) was dissolved in C_6_D_6_ (0.6 mL) and transferred to a J Youngs tap NMR tube. Propylene oxide (0.63 mg, 10.9 μmol, 0.76 μL, 1.0 eq) was subsequently added, and the reaction mixture was analysed by ^1^H NMR spectroscopy. The resultant ^1^H NMR spectrum confirmed the synthesis of complex **4** (Figure S76). The ^1^H NMR spectrum was identical to that generated from reacting [^Dipp^NacnacMg]_2_ with propylene oxide (2 eq) to produce **4**, without the generation of propene (which is the by-product of propylene oxide deoxygenation by [^Dipp^NacnacMg]_2_ **1**, Scheme 4).


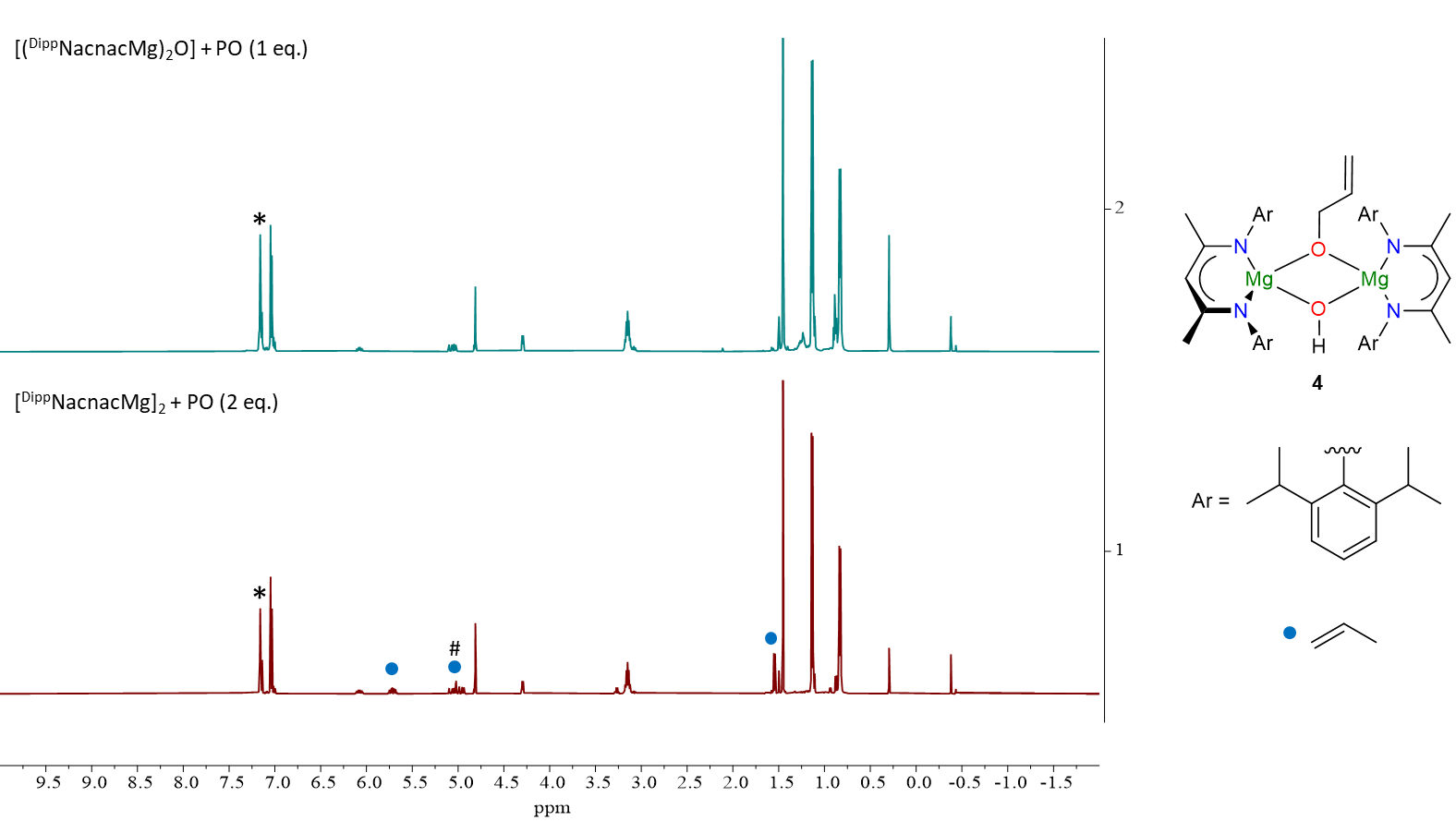


**Figure S76:** Stacked ^1^H NMR spectra for the NMR scale synthesis of complex **4** from [(^Dipp^NacnacMg)_2_O] (top, blue) and [^Dipp^NacnacMg]_2_ (bottom, maroon) in C_6_D_6_. *Overlapping peak with C_6_D_6_, ^#^overlapping peak with propene.

**Alternative Synthesis of [(^Dipp^NacnacMg)_2_O•*d*_8_-THF_2_] (8•*d*_8_-THF_2_)**

In a vial in a glovebox, [^Dipp^NacnacMg]_2_ **1** (10.4 mg, 11.8 μmol, 1 eq) was dissolved in *d*_8_-THF (0.5 mL) and transferred to a J Youngs tap NMR tube. Propylene oxide (0.68 mg, 11.7 μmol, 0.82 μL, 1 eq) was subsequently added. Upon shaking, the solution turned from orange to colourless and the reaction mixture was subsequently analysed by NMR spectroscopy.

Complex **8**•*d*_8_-THF_2_: ^1^H NMR (500 MHz, *d*_8_-THF, 300 K) δ 6.97 (dd, *J* = 8.5, 6.5 Hz, 4H, Ar-*H*), 6.94 – 6.90 (m, 8H, Ar-*H*), 4.63 (s, 2H, Nacnac-C*H*), 3.00 (dq, *J* = 13.8, 6.7 Hz, 8H, C*H*(CH_3_)_2_), 1.40 (s, 12H, Nacnac-C*H*_3_), 1.03 (d, *J* = 6.9 Hz, 24H, CH(C*H*_3_)_2_), 0.82 (d, *J* = 7.0 Hz, 24H, CH(C*H*_3_)_2_).

N.B. The ^1^H NMR spectrum matched that of [(^Dipp^NacnacMg)_2_O] synthesised from literature protocols; both compounds were analysed in *d*_8_-THF solvent (Figure S78).^[7]^


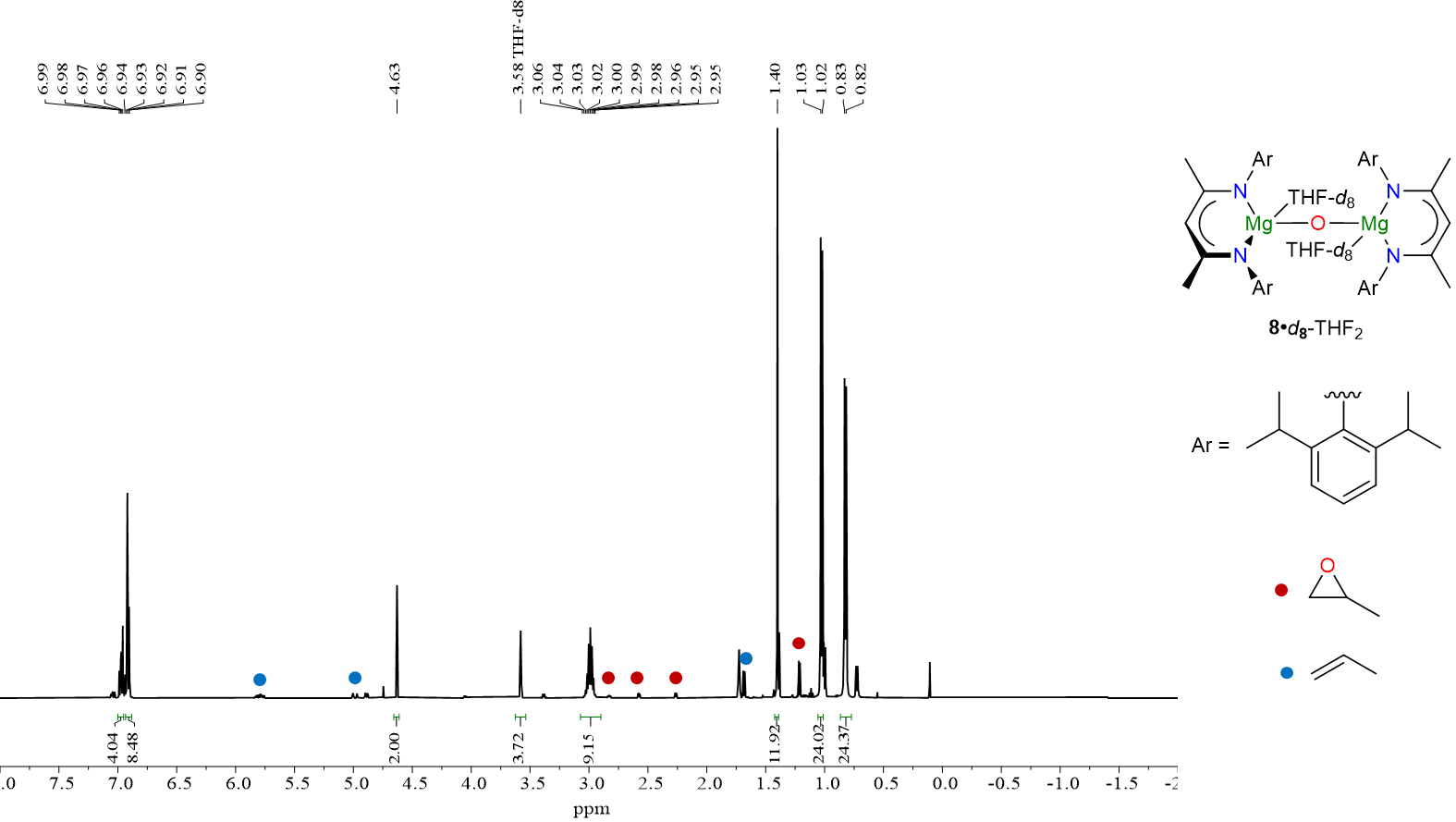


**Figure S77:** ^1^H NMR spectrum of **8**•d_8_-THF_2_ in d_8_-THF recorded at 500 MHz, 300 K.

**Figure S78:** Stacked ^1^H NMR spectra of **8**•d_8_-THF_2_ synthesised according to literature (top, blue) and from the addition of PO to [^Dipp^NacnacMg]_2_ in d_8_-THF (bottom, maroon). NMR spectra recorded in d_8_-THF, 300 K.

**Comparison of [(^Dipp^NacnacMg)_2_O•THF_2_] (8•THF_2_) and [(^Dipp^NacnacMg)_2_S•THF_2_] (13•THF_2_) in *d*_8_-THF**

Comparison of the ^1^H NMR spectra of the analogous oxo-bridged **8**•THF_2_ and sulfido-bridged **13**•THF_2_ shows clear similarities between the two complexes in terms of the complex symmetry, the coupling patterns and the chemical shifts (Figure S79). While the chemical shifts are fairly consistent, the *^i^*Pr-groups are significantly more shielded in **8**•THF_2_ than for **13**•THF_2_ (C*H*, δ = 1.40 vs 1.53 ppm; C*H*_3_ δ = 0.82 *vs* 0.95 ppm, respectively).

Complex **8**•THF_2_: ^1^H NMR (500 MHz, *d*_8_-THF, 300 K) δ 6.97 (dd, *J* = 8.5, 6.5 Hz, 4H, Ar-*H*), 6.94 – 6.90 (m, 8H, Ar-*H*), 4.63 (s, 2H, Nacnac-C*H*), 3.00 (dq, *J* = 13.8, 6.7 Hz, 8H, C*H*(CH_3_)_2_), 1.40 (s, 12H, Nacnac-C*H*_3_), 1.03 (d, *J* = 6.9 Hz, 24H, CH(C*H*_3_)_2_), 0.82 (d, *J* = 7.0 Hz, 24H, CH(C*H*_3_)_2_).

Complex **13**•THF_2_: ^1^H NMR (500 MHz, *d*_8_-THF, 300 K) δ 6.92 (s, 12H, Ar-*H*), 4.64 (s, 2H, Nacnac-C*H*), 3.65 – 3.60 (m, 8H, THF-OC*H*_2_CH_2_), 3.06 – 2.98 (m, 8H), 1.80 – 1.75 (m, 8H, THF-OCH_2_C*H*_2_), 1.53 (s, 12H, Nacnac-C*H*_3_), 1.03 (d, *J* = 6.5 Hz, 24H, CH(C*H*_3_)_2_), 0.95 (d, *J* = 6.8 Hz, 24H, CH(C*H*_3_)_2_).


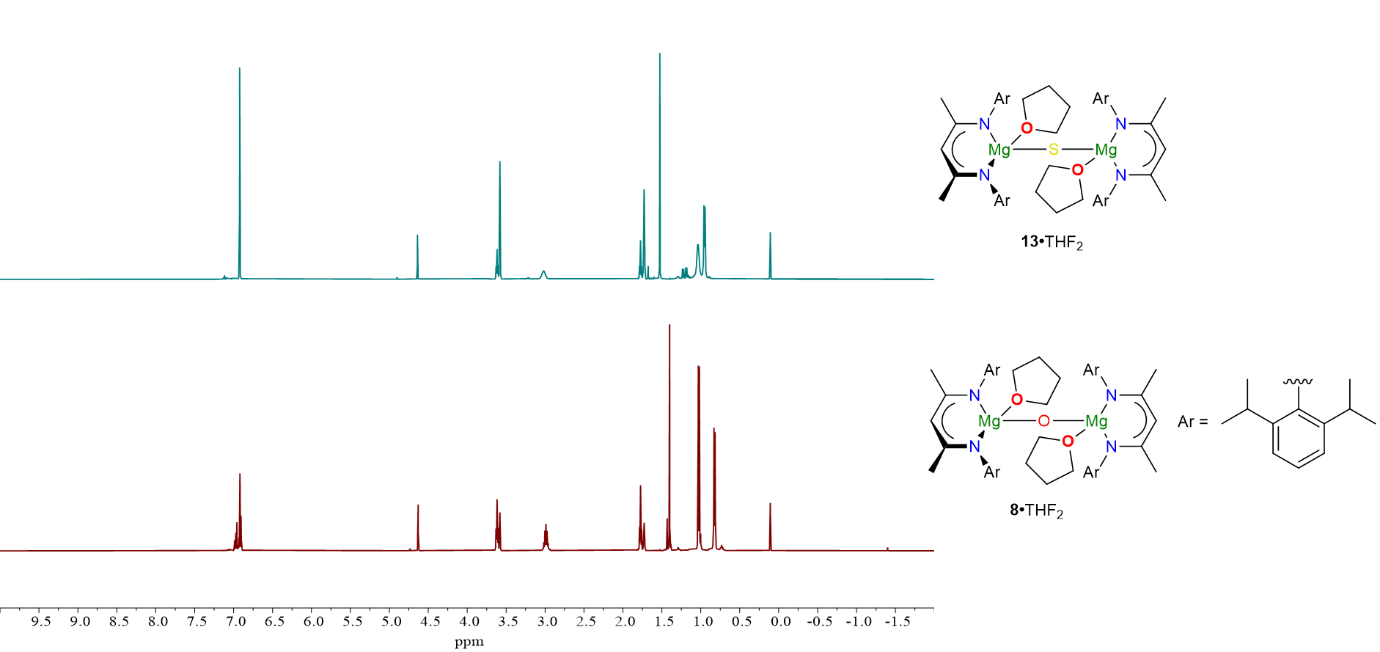


**Figure S79:** Stacked ^1^H NMR spectra of oxo-bridged **8**•THF_2_ (bottom, maroon) and sulfido-bridged **13**•THF_2_ (top, blue) in d_8_-THF recorded at 500 MHz, 300 K.

### **Comparison of Hydroxide Shifts in ^Dipp^Nacnac Magnesium Complexes 4 and 9-11**

Examining the ^1^H NMR spectra for complexes (**4**, **9**, **10a**, **10b** and **11a**) shows a different chemical shift for the hydroxide proton in each complex (Figure S80), which shows that the heteroleptic structures are retained in solution. These -O*H* resonances also have different chemical shifts compared to the homoleptic hydroxide dimer [^Dipp^NacnacMgOH]_2_ **7** (δ = -0.44 ppm).^[7]^


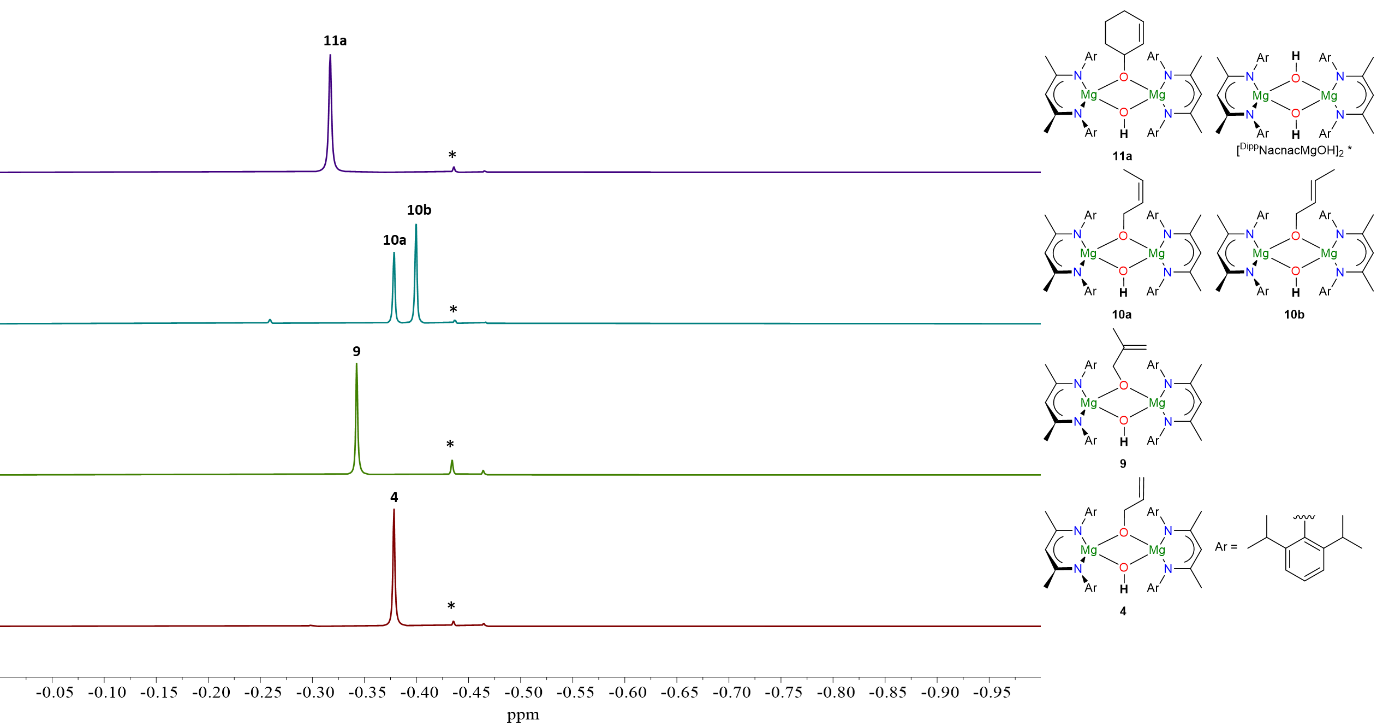


**Figure S80:** Stacked ^1^H NMR spectra highlighting the differences in chemical shift for the OH peak for complexes **4** (maroon, -0.38 ppm), **9** (green, -0.34 ppm), **10a** (blue, -0.38 ppm), **10b** (blue, -0.40 ppm) and **11a** (purple, -0.32 ppm) in C_6_D_6_. Traces of the dihydroxyl magnesium dimer [^Dipp^NacnacMgOH]_2_ **7** are also identified (*, -0.44 ppm).

### **Stability of Heteroleptic Complexes**

While the *in situ* formation of **4**, **9**, **10a**, **10b** and **11a** at room temperature showed no redistribution to homoleptic [^Dipp^NacnacMgOH]_2_ **7** (Figure S80), attempts to isolate the heteroleptic complexes by removing the solvent *in vacuo* did give redistribution into [^Dipp^NacnacMgOH]_2_ **7** along with the homoleptic alkoxide dimers. This was evidenced by the presence of new resonances in the ^1^H NMR spectra, including [^Dipp^NacnacMgOH]_2_ **7**.^[7]^ Additionally, crystals of the homoleptic dimer **11b** were obtained from the reaction of [^Dipp^NacnacMg]_2_ **1** with cyclohexene oxide, rather than heteroleptic **11a** which was formed *in situ*. The ^Dep^Nacnac and ^Mes^Nacnac analogues **5** and **6** also showed new ^1^H NMR resonances upon removing the solvent *in vacuo*, including resonances at δ = - 0.48 and δ = - 0.52 ppm, respectively, that were assigned as Mg-O*H* units of the hydroxide dimers [^Ar^NacnacMgOH]_2_ (Ar = 2,6-diethylphenyl and 2,4,6-trimethylphenyl). Heating heteroleptic **4**-**6** and **9**-**11a** to 80 °C showed that redistribution between the heteroleptic and homoleptic complexes occurred to varying extents, along with some decomposition. Overall, these observations highlight that complexes **4**-**6** and **9**-**11a** are not thermally stable.


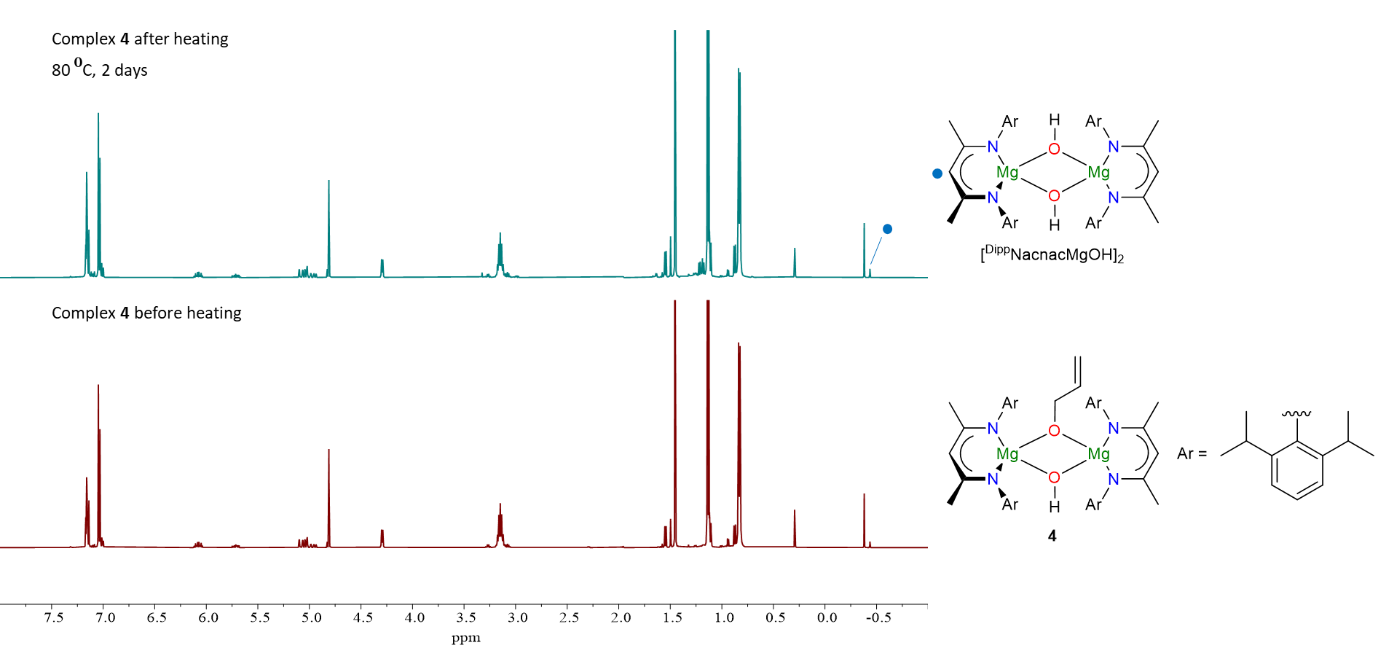


**Figure S81:** Stacked ^1^H NMR spectra of [^Dipp^NacnacMg(OH)(OCH_2_CHCH_2_)Mg^Dipp^Nacnac] **4** before (maroon) and after (blue) heating (80 ⁰C, 2 days). After heating there is a slight increase in [^Dipp^NacnacMgOH]_2_ **7**, identified from diagnostic ^1^H NMR resonances including the -OH peak at -0.44 ppm.


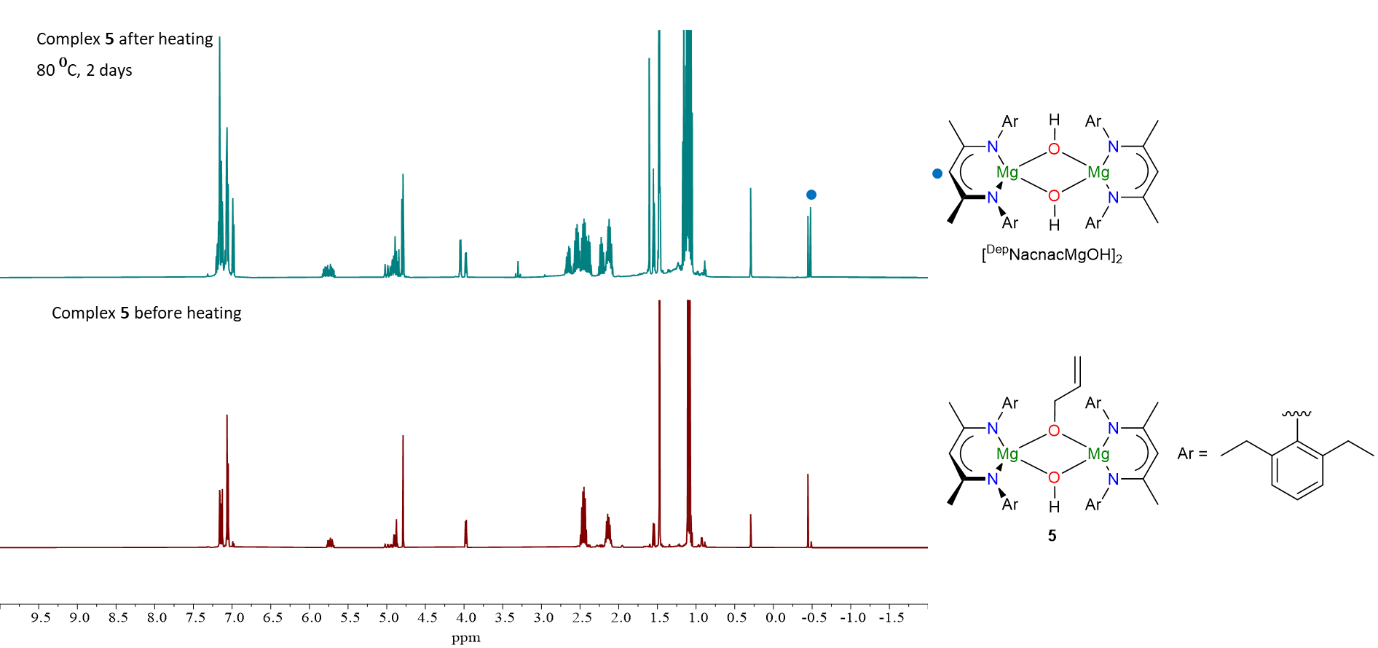


**Figure S82:** Stacked ^1^H NMR spectra of [^Dep^NacnacMg(OH)(OCH_2_CHCH_2_)Mg^Dep^Nacnac] **5** before (maroon) and after (blue) heating (80 ⁰C, 2 days). After heating there is an increase in [^Dep^NacnacMgOH]_2_, identified from diagnostic ^1^H NMR resonances including the -OH peak at -0.48 ppm.


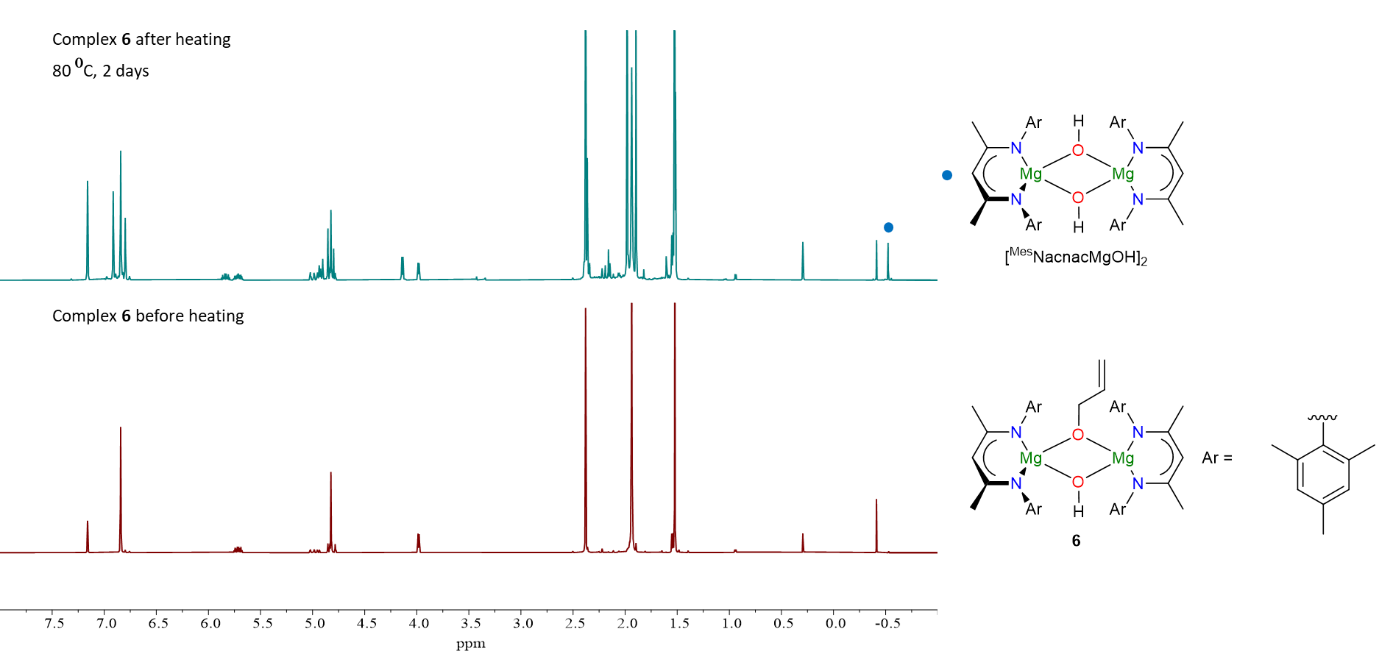


**Figure S83:** Stacked ^1^H NMR spectra of [^Mes^NacnacMg(OH)(OCH_2_CHCH_2_)Mg^Mes^Nacnac] **6** before (maroon) and after (blue) heating (80 ⁰C, 2 days). After heating there is an increase in [^Mes^NacnacMgOH]_2_, identified from diagnostic ^1^H NMR resonances including the -OH peak at -0.52 ppm.


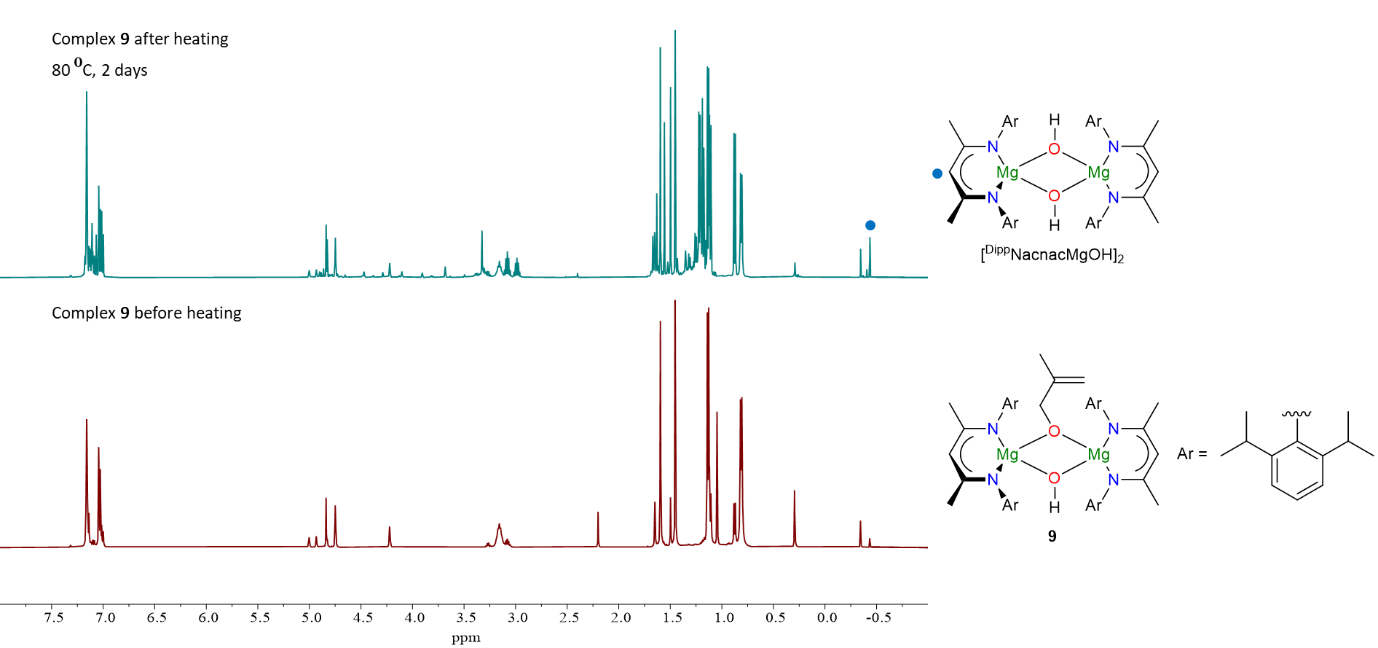


**Figure S84:** Stacked ^1^H NMR spectra of complex **9** before (maroon) and after (blue) heating (80 ⁰C, 2 days). After heating there is an increase in [^Dipp^NacnacMgOH]_2_ **7**, identified from diagnostic ^1^H NMR resonances including the -OH peak at -0.44 ppm.


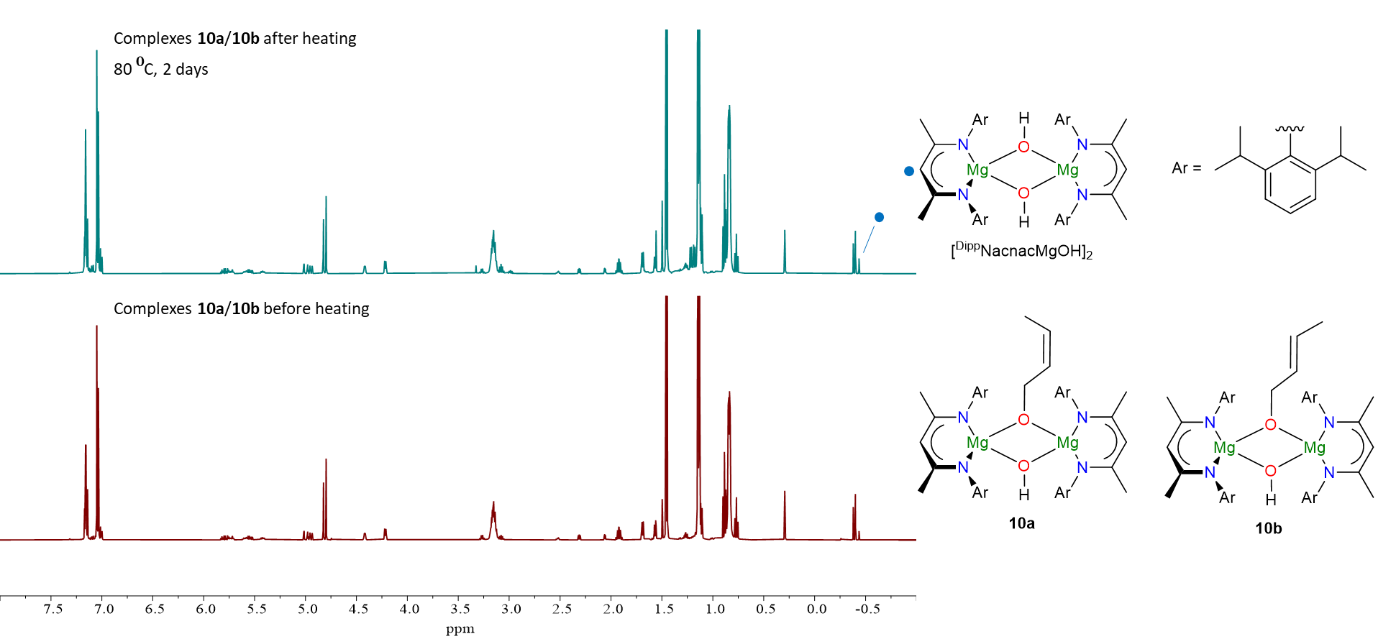


**Figure S85:** Stacked ^1^H NMR spectra of complex **10a-b** before (maroon) and after (blue) heating (80 ⁰C, 2 days). After heating there is an increase in [^Dipp^NacnacMgOH]_2_ **7**, identified from diagnostic ^1^H NMR resonances including the -OH peak at -0.44 ppm.


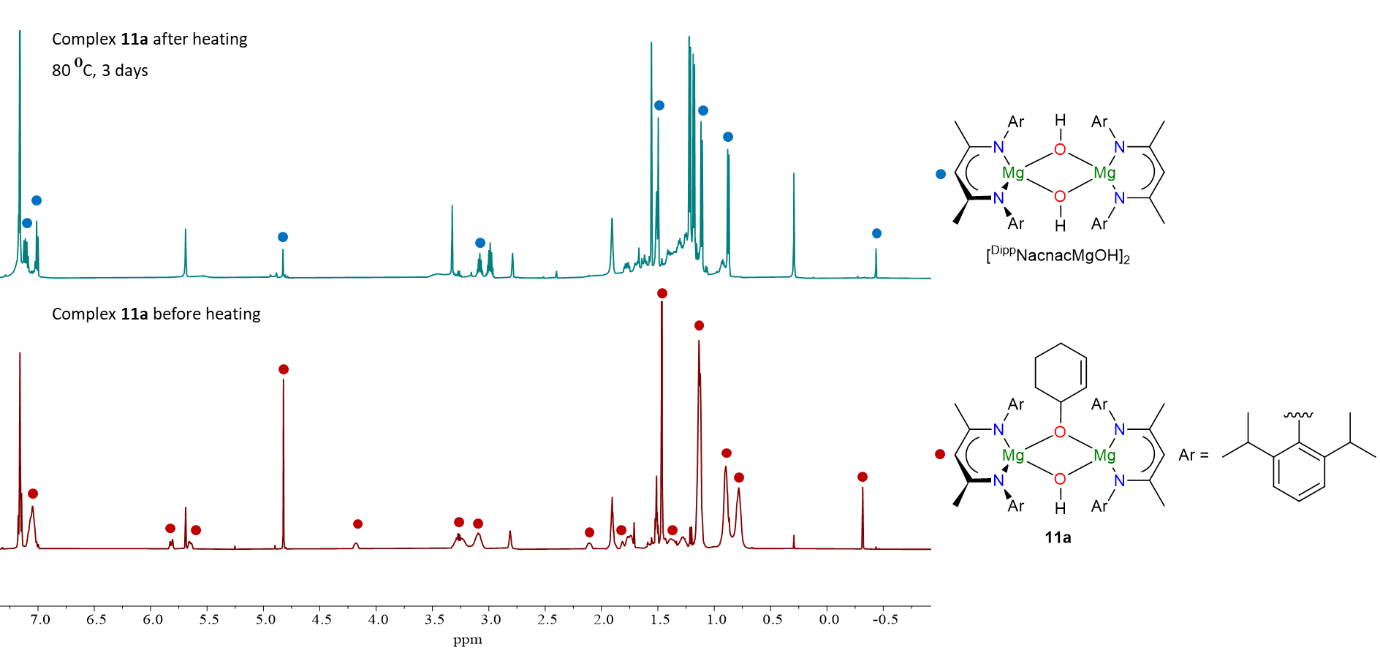


**Figure S86:** Stacked ^1^H NMR spectra of complex **11a** before (maroon) and after (blue) heating (80 ⁰C, 3 days). After heating **11a** has fully redistributed to [^Dipp^NacnacMgOH]_2_ **7**, identified from diagnostic ^1^H NMR resonances including the -OH peak at -0.44 ppm.

**NMR Scale Synthesis of [^Dipp^NacnacMgS]_2_ (12) Showing Elimination of Propene**

In a glovebox, [^Dipp^NacnacMg]_2_ (10.0 mg, 11.3 μmol, 1 eq) was dissolved in C_6_D_6_ (0.6 mL) and transferred to a J Youngs tap NMR tube followed by propylene sulfide (1.8 mg, 24.9 μmol, 1.96 μL, 2.2 eq). Upon shaking the solution turned from yellow to colourless to yellow again and the reaction mixture was subsequently analysed by NMR spectroscopy. Resonances for complex **12** and propene can be clearly seen in the ^1^H NMR spectrum. Propene was removed *in vacuo* and the sample redissolved in C_6_D_6_ to give **12**.

*Overlap with C_6_D_6_


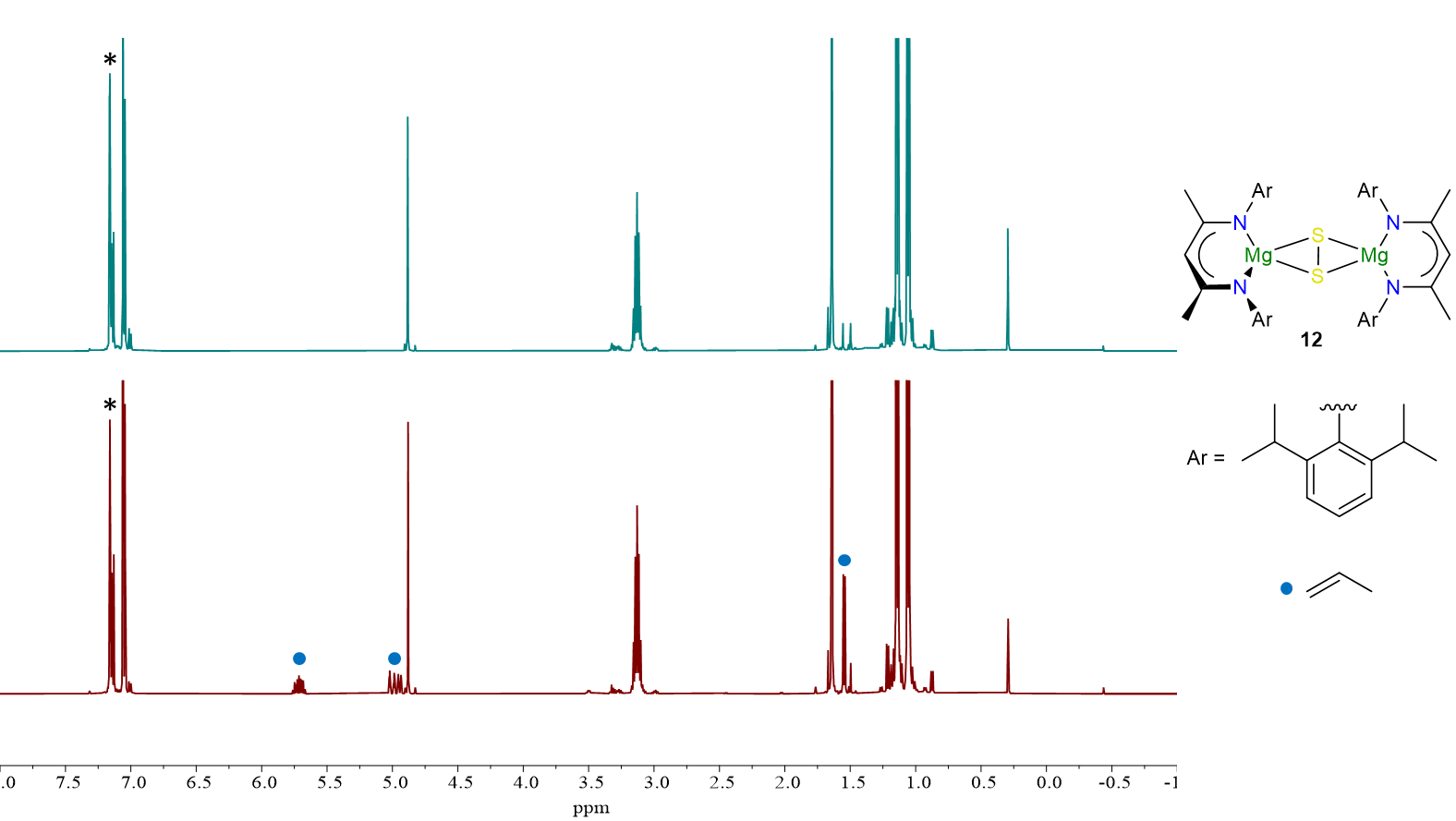


**Figure S87:** ^1^H NMR spectrum of NMR scale synthesis of **12** in C_6_D_6_ recorded at 500 MHz, 300 K. Initial ^1^H NMR spectrum (bottom, maroon) shows **12** and elimination of propene. Propene was removed from the reaction mixture in vacuo which is spectroscopically observable after redissolution in C_6_D_6_. (top, blue).

**NMR Scale Synthesis of [(^Dipp^NacnacMg)_2_S•THF_2_] (13•THF_2_) Showing Elimination of Propene**

In a vial in a glovebox, [^Dipp^NacnacMg]_2_ **1** (9.4 mg, 10.6 μmol, 1 eq) was dissolved in C_6_D_6_ (0.6 mL) and transferred to a J Youngs tap NMR tube. An excess of THF (10 µL) was subsequently added, and the reaction mixture was shaken to give an orange solution of the THF adduct of **1**. Propylene sulfide (1.74 mg, 23.4 μmol, 1.84 μL, 2.2 eq) was added and upon shaking, the solution turned from orange to colourless. The reaction mixture was subsequently analysed by NMR spectroscopy. Resonances for complex **13**•THF*_2_*, propene and excess propylene sulfide can be clearly seen in the ^1^H NMR spectrum. Propene and excess propylene sulfide were removed *in vacuo* and the sample redissolved in C_6_D_6_ to give **13**•THF*_2_*.

^#^Overlap with propylene sulfide


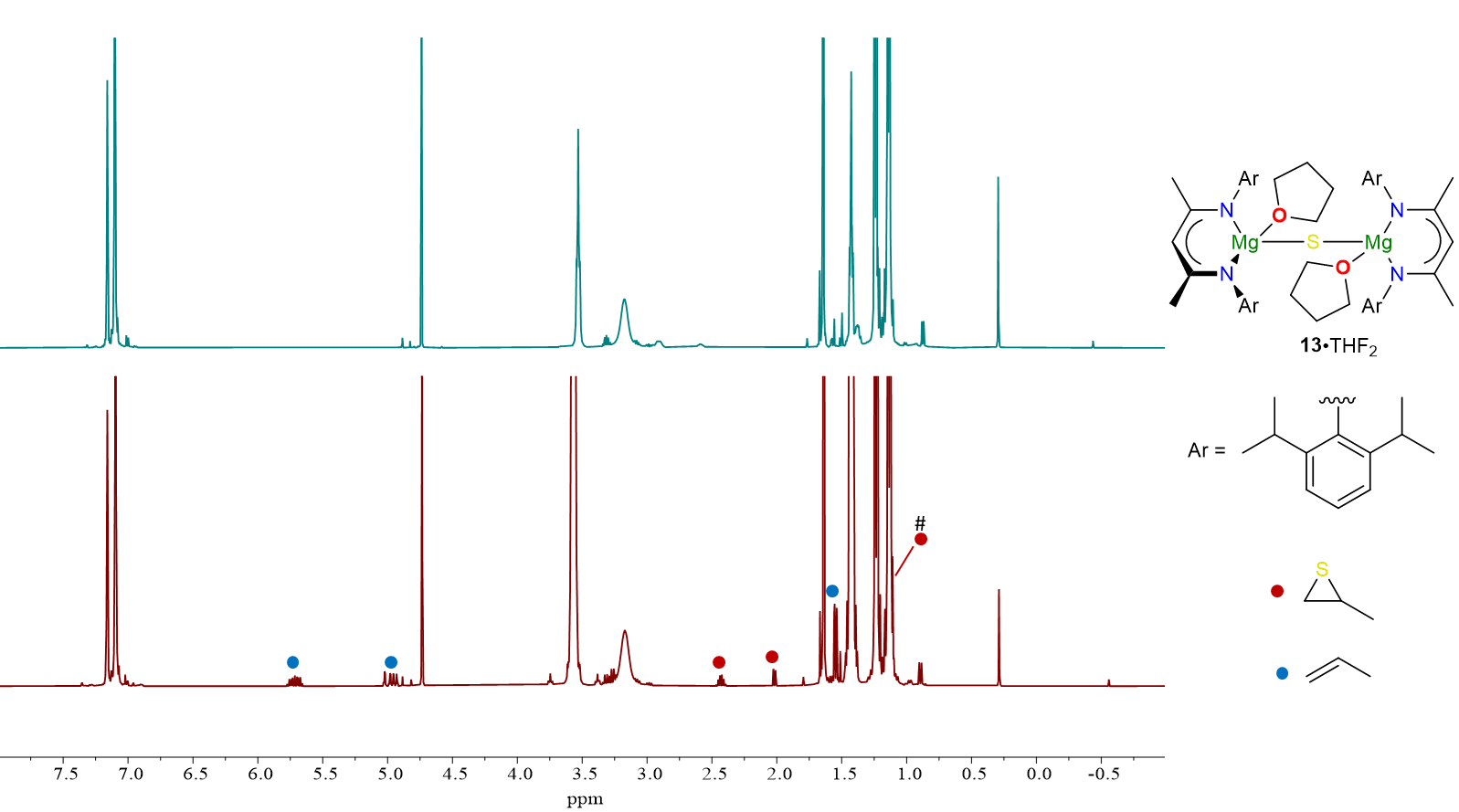


**Figure S88:** ^1^H NMR spectrum of NMR scale synthesis of **13**•THF_2_ in C_6_D_6_ recorded at 500 MHz, 300 K. Initial ^1^H NMR spectrum (bottom, maroon) shows **13**•THF_2_, excess propylene sulfide and elimination of propene. Propene and propylene sulfide were both removed from the reaction mixture in vacuo, which is spectroscopically observable after redissolution in C_6_D_6_. (top, blue) ^#^Overlapping peak with propylene sulfide.

**Comparison of [^Dipp^NacnacMg]_2_ with PO (2 eq) and PS (2 eq)**

Comparing complexes **4** and **12** shows a clear trend for the downfield shift of ligand peaks for complex **12** *versus* **4**. These shifts are noticeable for the Nacnac backbone C*H* of 4.81 and 4.88 ppm, the Dipp C*H* 1.45 and 1.64 ppm, and a Dipp C*H*_3_ group of 0.83 and 1.06 for complexes **4** and **12** respectively.


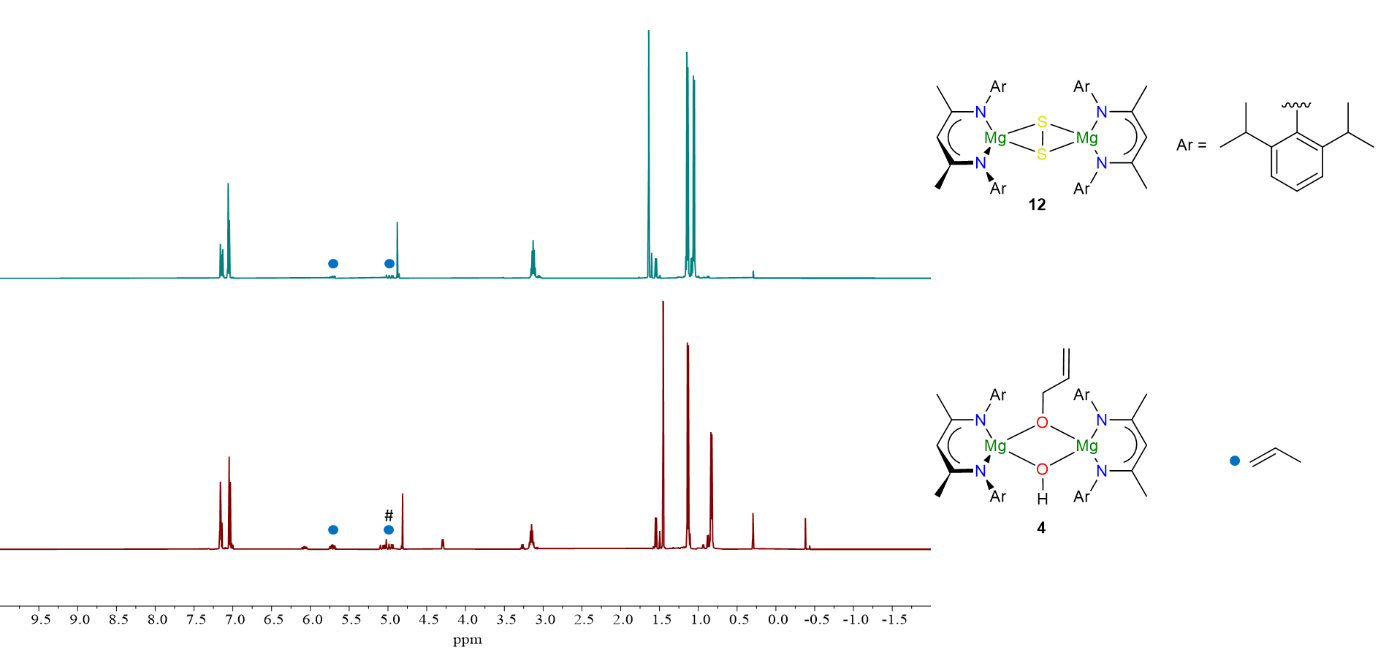


**Figure S89:** ^1^H NMR spectrum for the in situ NMR scale synthesis of **4** in C_6_D_6_ with PO (2 eq) recorded at 500 MHz, 300 K (bottom, maroon), ^#^overlap of propene with **4**. ^1^H NMR spectrum for the in situ NMR scale synthesis of **12** in C_6_D_6_ with PS (2 eq) recorded at 500 MHz, 300 K (top, blue). Propene identified.

**Reactivity of [^Dipp^NacnacMg]_2_ with PO in *d*_8_-THF**

Adding PO (2 eq) to [^Dipp^NacnacMg]_2_ in *d*_8_-THF spectroscopically initially gives [(^Dipp^NacnacMg)_2_O] **8**•*d*_8_-THF*_2_* (analysed by NMR spectroscopy after approx. 30 min) as the major product, with a minor amount of **4** and excess PO. After leaving this reaction mixture for 1 day the major product is **4**, demonstrating that **8**•*d*_8_-THF*_2_* is formed as an intermediate species. Crystals formed in the NMR tube over the following four days and were characterised as the THF solvate of **4** (see Figure S97).


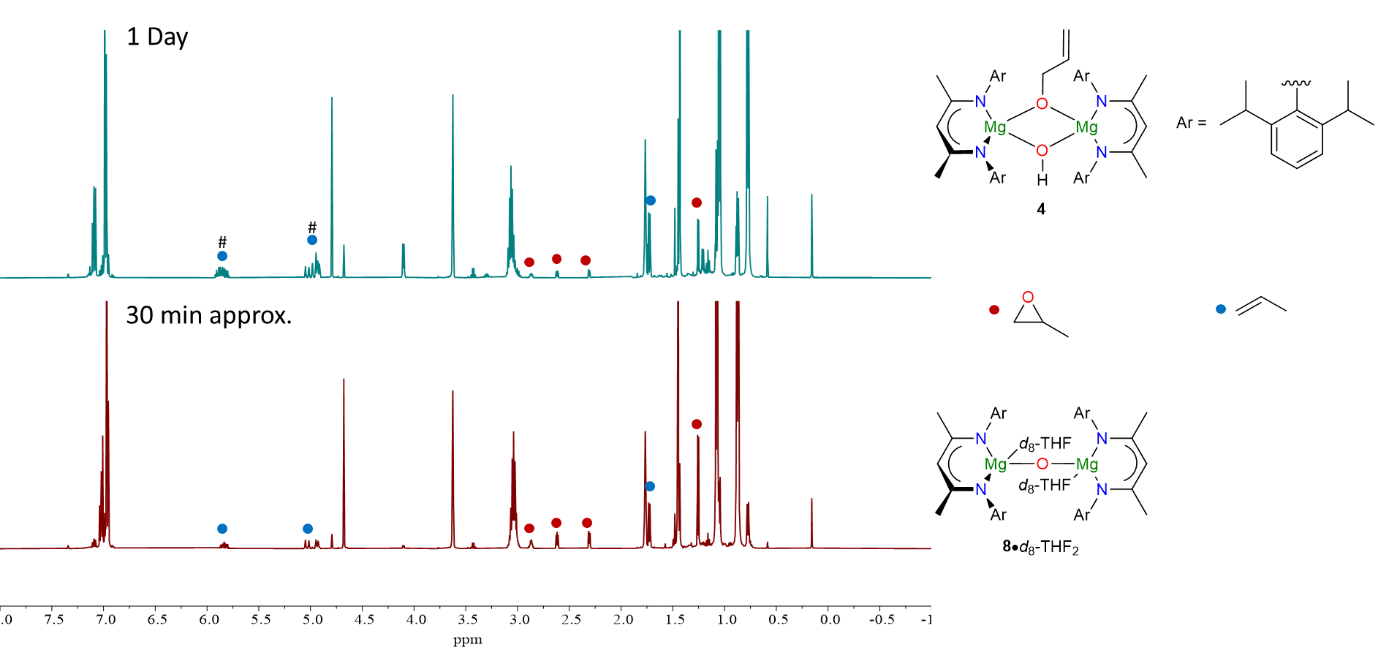


**Figure S90:** ^1^H NMR spectrum for the in situ NMR scale reaction of **1** with PO (2 eq) in d_8_-THF, recorded at 500 MHz, 300 K. Analysing the reaction after 30 min shows **8**•d_8_-THF_2_ as the major product (bottom, maroon), and after 1 day the major product is **4**. Propene and propylene oxide identified. ^#^Overlapping peak of propene and **4**.

**Reactivity of [^Dipp^NacnacMg]_2_ with PO in C_6_D_6_**

Adding PO (1 eq) to [^Dipp^NacnacMg]_2_ in C_6_D_6_ spectroscopically gives 50% conversion to alkoxide/hydroxide **4** with concomitant propene elimination. Adding further PO (1 eq, *i.e.* 2 eq in total) gives **4** along with propene.


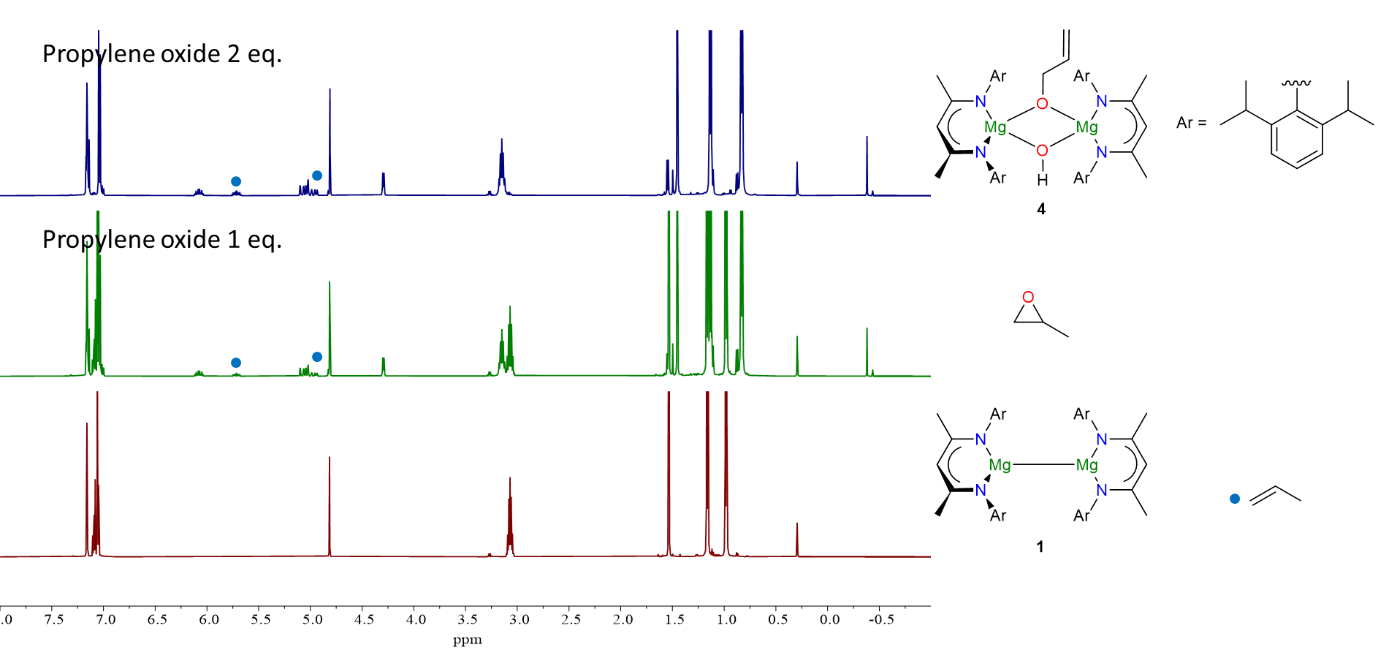


**Figure S91:** ^1^H NMR spectrum for the reaction of **1** (bottom, maroon) with 1 equivalent (middle, green) and 2 equivalents (top, blue) of propylene oxide to give **4**, recorded at 500 MHz, 300 K.

**Reactivity of [^Dipp^NacnacMg]_2_ with PS in C_6_D_6_**

Adding PS (1 eq) to [^Dipp^NacnacMg]_2_ in C_6_D_6_ gives 50% conversion to disulfide **12** with concomitant propene elimination, as determined by ^1^H NMR spectroscopy. Adding further PS (1 eq, *i.e.* 2 eq in total) gives disulfide-bridged **12** along with propene.


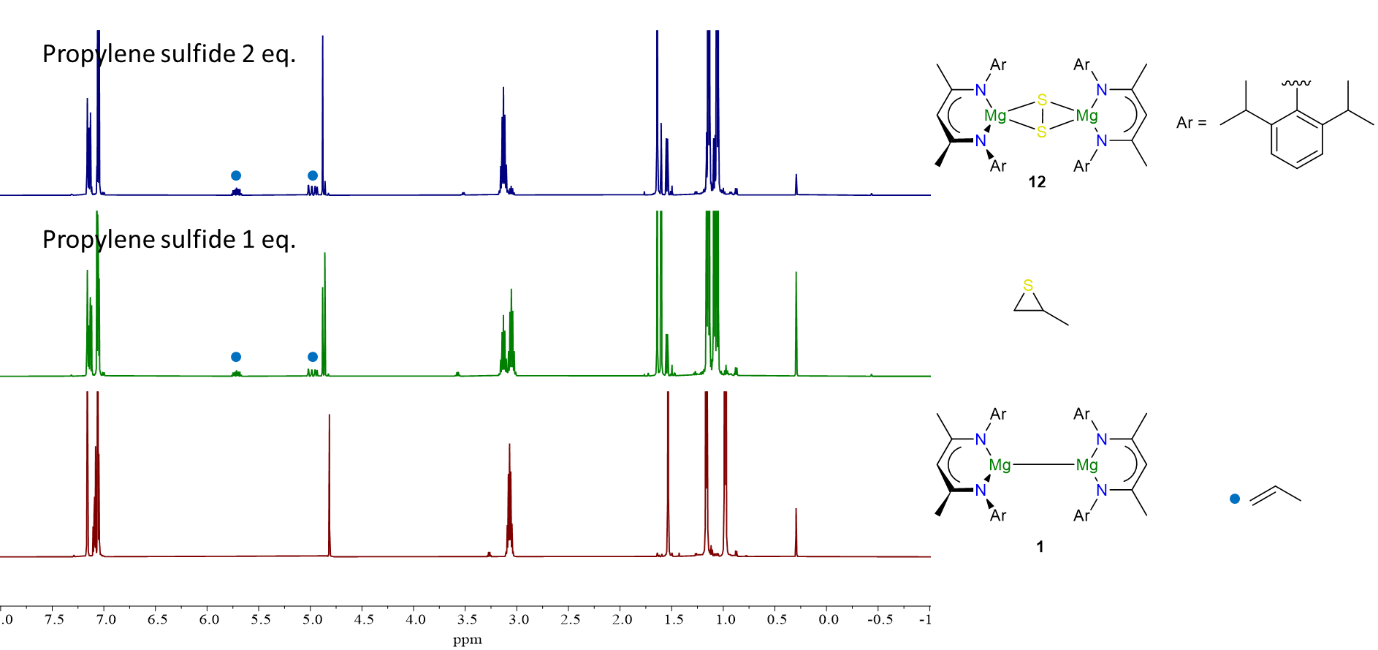


**Figure S92:** ^1^H NMR spectrum for the reaction of **1** (bottom, maroon) with 1 equivalent (middle, green) and 2 equivalents (top, blue) of propylene sulfide to give **12**, recorded at 500 MHz, 300 K.

### **Synthesis of Complex 12 from 13•(Et_2_O)_2_**

In a glovebox, [^Dipp^NacnacMg]_2_ (12.4 mg, 14.0 μmol, 1.0 eq) was suspended in Et_2_O (1.0 mL) followed by propylene sulfide (2.1 mg, 28.0 μmol, 2.21 μL, 2.0 eq) and the reaction mixture transferred to a J Youngs tap NMR tube. The reaction mixture was dried *in vacuo* to give crude [(^Dipp^NacnacMg)_2_S•(OEt_2_)_2_] (**13**•(Et_2_O)_2_) as an off-white solid. The reaction mixture was redissolved in C_6_D_6_ (0.6 mL) to give a clear, colourless solution followed by the addution of propylene sulfide (1.1 mg, 15.2 μmol, 1.2 μL, 1.0 eq) to give a yellow solution upon shaking. The reaction mixture was dried *in vacuo* (to remove propene and Et_2_O) and redissolved in C_6_D_6_ (0.6 mL). The resultant ^1^H NMR spectrum confirmed the synthesis of disulfide-bridge **12** (Figure S88). The ^1^H NMR spectrum was identical to that generated from reacting [^Dipp^NacnacMg]_2_ with propylene sulfide (2 eq) to produce **12**, demonstrating that the formation of **12** can proceed *via* **13**•(Et_2_O)_2_ as an intermediate.


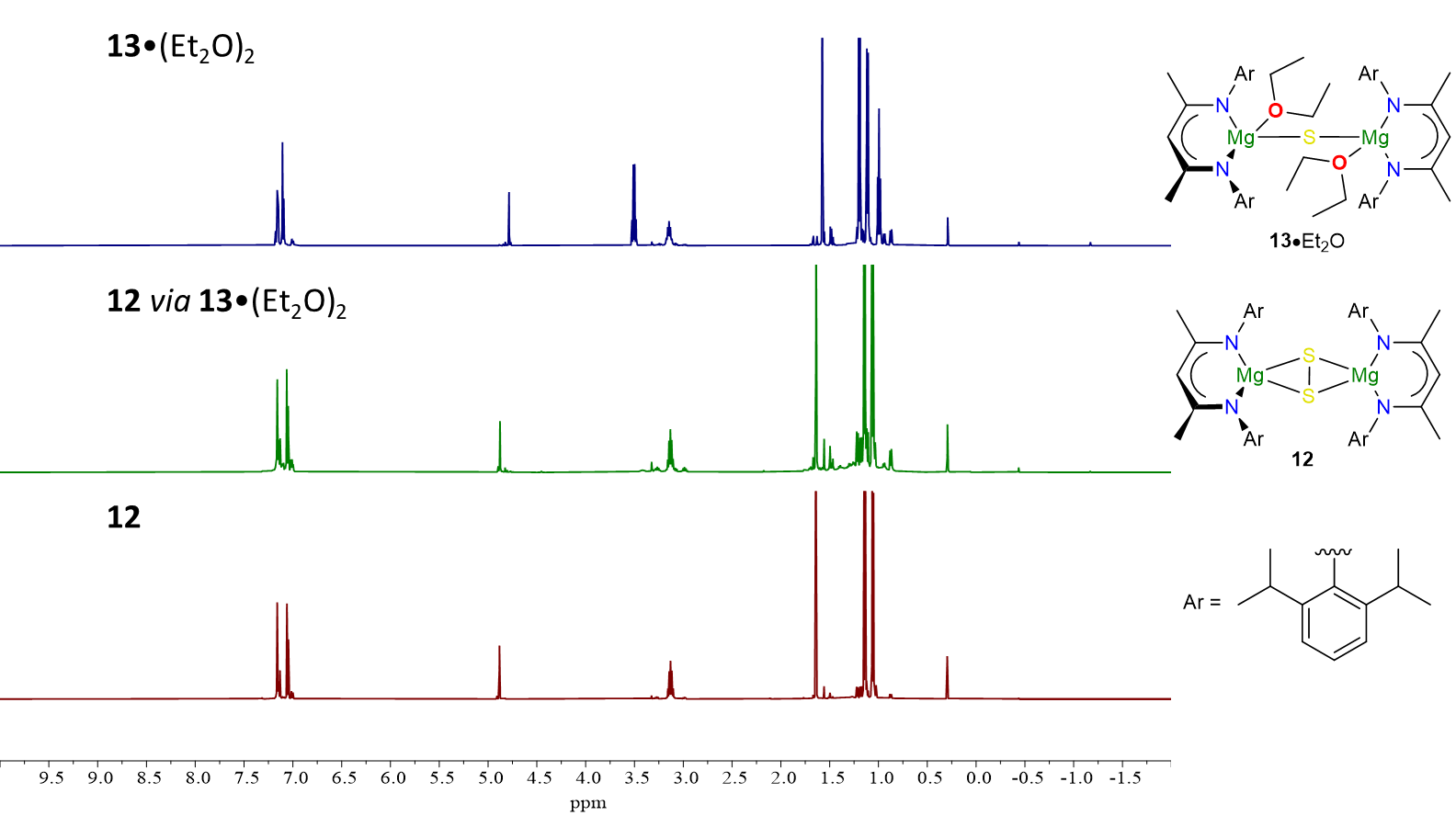


**Figure S93:** Stacked ^1^H NMR spectra for sulfido-bridged **13**•(Et_2_O)_2_ prepared in situ from the reaction of [^Dipp^NacnacMg]_2_ and propylene sulfide (top, blue), disulfide-bridged **12** synthesised via **13**•(Et_2_O)_2_ and 1 eq of propylene sulfide (middle, green), and crystals of disulfide-bridged **12** (bottom, maroon) in C_6_D_6_.

### **Proposed Mechanisms for the Reactivity of [^Dipp^NacnacMg]_2_ with Propylene Oxide**

Key observations underpinning the proposed mechanism are:

- Addition of propylene oxide (PO) (1 eq.) to [^Dipp^NacnacMg]_2_ **1** in non-coordinating C_6_D_6_ gives 50% conversion to heteroleptic **4** with 50% unreacted **1** remaining.
- In coordinating *d*_8_-THF addition of PO (1 eq.) to **1** gives 100% conversion to oxo-bridged **8**•*d*_8_-THF.
- Addition of PO to oxo-bridged **8** gives **4**, in both C_6_D_6_ and *d*_8_-THF.
- Addition of *d*_6_-PO to oxo-bridged **8** gives **4**, with the O*H* now an O*D* (*i.e.* to give **4D**).
- An analogous Mg-O-C-C-M intermediate for the deoxygenation of epoxides has previously been proposed in a low-valent magnesium system.^[13]^ We note that other epoxide deoxygenation mechanisms may also be possible, such as a concerted mechanism.^[34-35]^

Potential mechanism for the reactivity of **1** with PO in C_6_D_6_ (see Figure S94):

1. Coordination of one equivalent of PO activates the Mg-Mg bond of **1** to give **Int.1**.
2. Ring-opening insertion of a second equivalent of PO gives the magnesium alkyl/alkoxide bridged intermediate(s) **Int.2a** and/or **Int.2b**, depending on the orientation of the epoxide insertion.
3. Alkene elimination from **Int.2a** and/or **Int.2b** gives oxo-bridged **Int.3**, with PO coordinated.
4. The coordinated PO of **Int.3** is primed to react *via* deprotonation and rearrangement to give heteroleptic **4**.

**Figure S94:** Potential mechanism for the reaction of **1** with PO in C_6_D_6_.

Potential mechanism for the reactivity of **1** with PO in THF (Figure S95):

1. Coordination of THF to **1** gives **1**•THF_2_, where the THF coordination is known to be labile.^[42]^
2. Loss of one THF molecule gives **Int.1** where the Mg-Mg bond is activated by the one molecule of coordinated THF. This allows ring-opening insertion of PO (1 eq.) to give **Int.2a** and/or **Int.2b**.
3. Alkene elimination from either **Int.2a**/**2b** gives THF coordinated oxo-bridged **Int.3** which is in equilibrium with **8**•THF_2_.
4. A second equivalent of epoxide undergoes ring-opening deprotonation with the loss of coordinated THF to give **4**.

**Figure S95:** Proposed mechanism for the reaction of **1** with PO in THF.

## **Single Crystal X-Ray Diffraction Data**


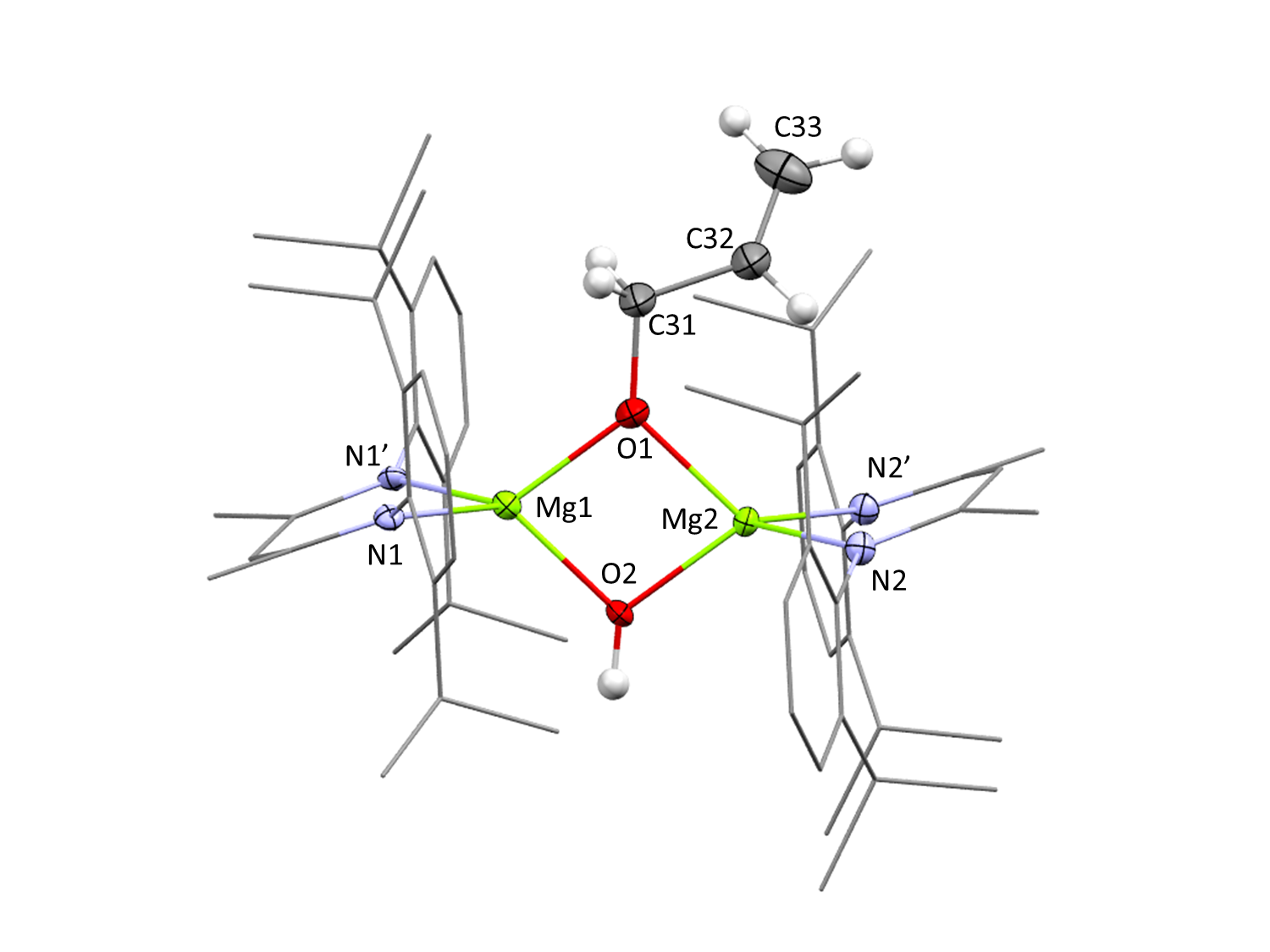


**Figure S96:** Structure of [^Dipp^NacnacMg(OH)OCH_2_CHCH_2_)Mg^Dipp^Nacnac] **4** obtained at room temperature from C_6_D_6_ overnight, minor disorder component is not shown. Selected hydrogen atoms are shown at a fixed radius. Selected parts of the ligand framework shown as wireframe. Thermal ellipsoids set to 50% probability. Selected bond lengths (Å) and angles (°) Mg1-O1 1.958(4), Mg1-O2 1.964(4), Mg2-O1 1.972(5), Mg2-O2 1.961(4), Mg1-Mg2 2.9958(12) O1-C31 1.434(6), C31-C32 1.500(6), C32-C33 1.238(8), Mg1-O1-Mg2 99.31(18) and Mg1-O2-Mg2 99.50(17).


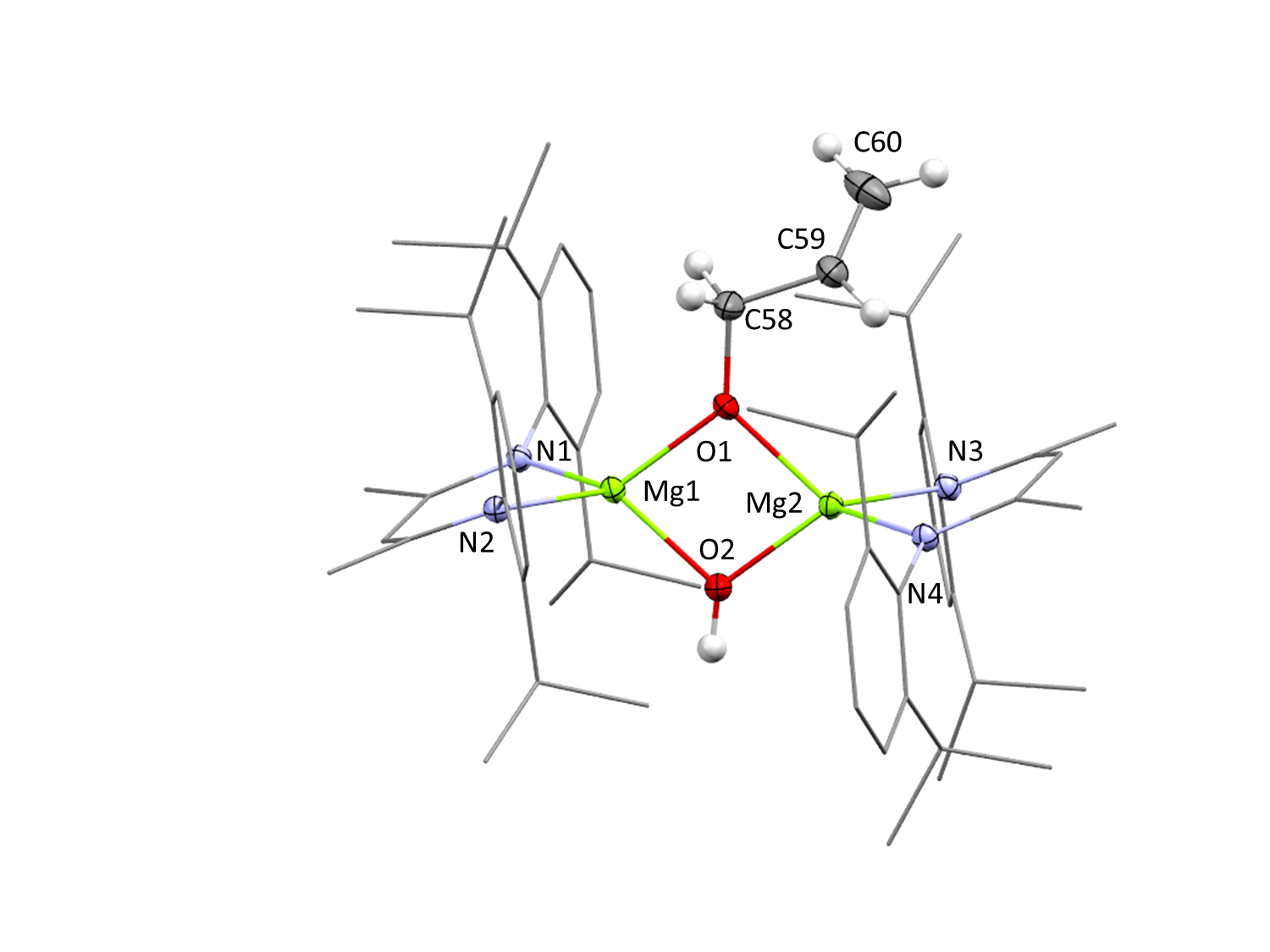


**Figure S97:** Structure of [^Dipp^NacnacMg(OH)OCH_2_CHCH_2_)Mg^Dipp^Nacnac] **4** obtained at room temperature from d_8_-THF over 5 days, minor disorder component is not shown. Selected hydrogen atoms are shown at fixed radii. Selected parts of the ligand framework shown as wireframe. Thermal ellipsoids set to 50% probability. Selected bond lengths (Å) and angles (°) Mg1-O1 1.965(2), Mg1-O2 1.966(2), Mg2-O1 1.978(2), Mg2-O2 1.948(2), Mg1-Mg2 2.9903(7) O1-C58 1.427(3), C58-C59 1.495(3), C59-C60 1.292(4), Mg1-O1-Mg2 98.63(9) and Mg1-O2-Mg2 99.63(9).


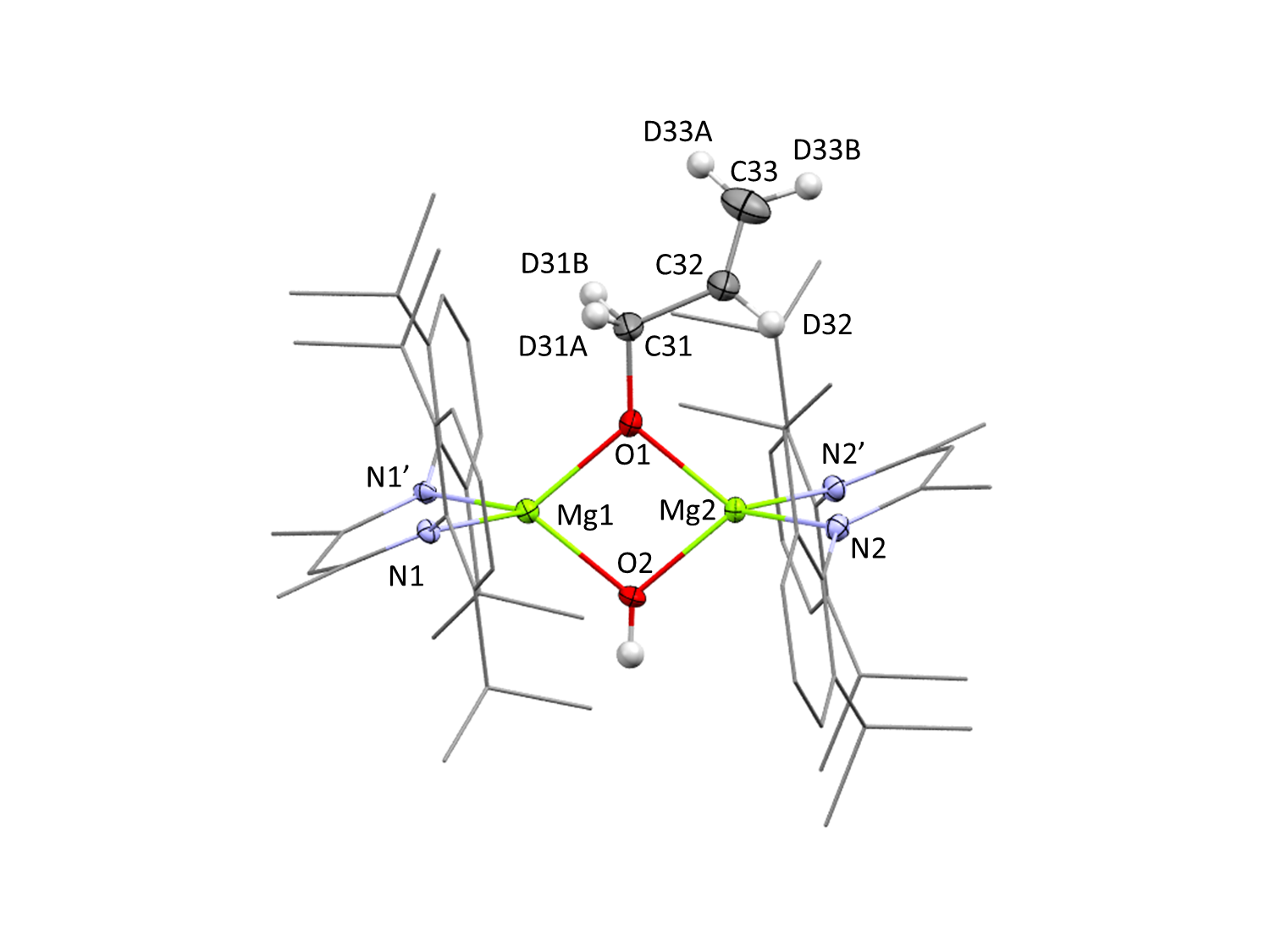


**Figure S98:** Structure of [^Dipp^NacnacMg(OD)OCD_2_CDCD_2_)Mg^Dipp^Nacnac] **4D** obtained at room temperature from C_6_D_6_ overnight, minor disorder component is not shown. Selected deuterium atoms are shown at fixed radii. Selected parts of the ligand framework shown as wireframe. Thermal ellipsoids set to 50% probability. Selected bond lengths (Å) and angles (°) Mg1-O1 1.974(4), Mg1-O2 1.954(4), Mg2-O1 1.964(4), Mg2-O2 1.965(4), Mg1-Mg2 2.9950(11), O1-C31 1.416(6), C31-C32 1.491(6), C32-C33 1.239(7), Mg1-O1-Mg2 99.03(17) and Mg1-O2-Mg2 99.66(16).


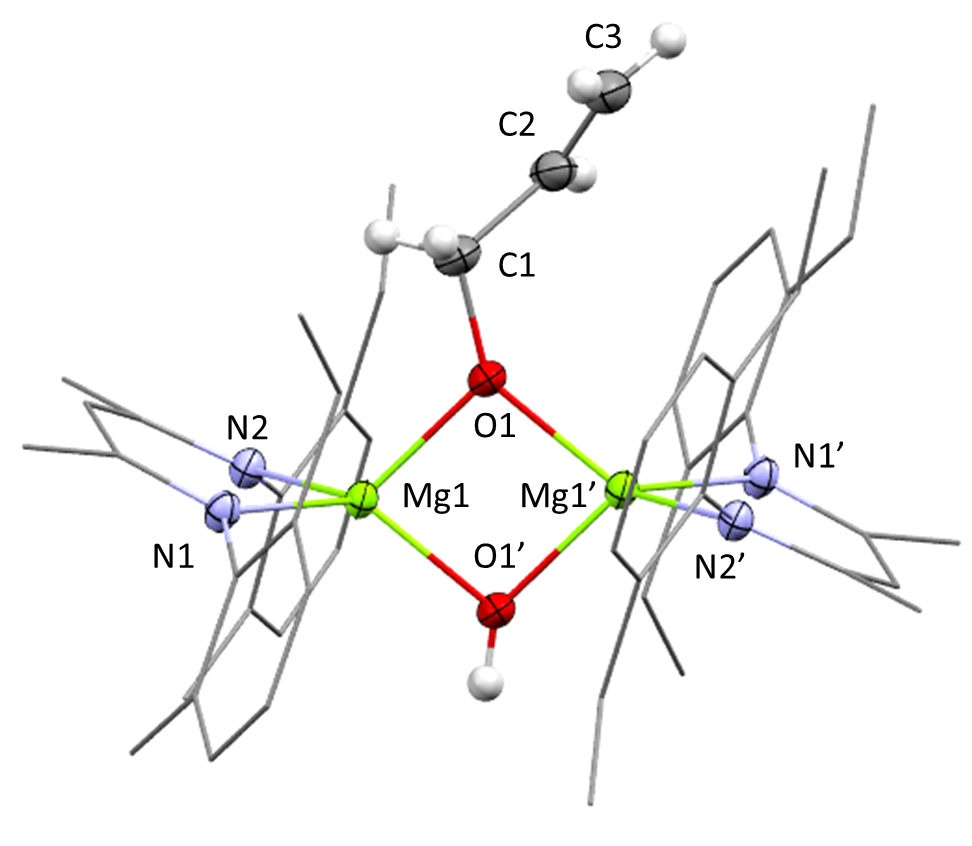


**Figure S99:** Structure of [^Dep^NacnacMg(OH)OCH_2_CHCH_2_)Mg^Dep^Nacnac] **5** obtained from toluene stored at -35 °C overnight, minor disorder component is not shown. Selected hydrogen atoms are shown at fixed radii. Selected parts of the ligand framework shown as wireframe. Thermal ellipsoids set to 50% probability. Selected bond lengths (Å) and angles (°) Mg1-O1 1.9574(7), Mg1-O2 1.9317(7), Mg1-Mg1’ 2.8944(6), O1-C1 1.417(2), C1-C2 1.479(3), C2-C3 1.315(3) and Mg1-O1-Mg1’ 96.19(3).


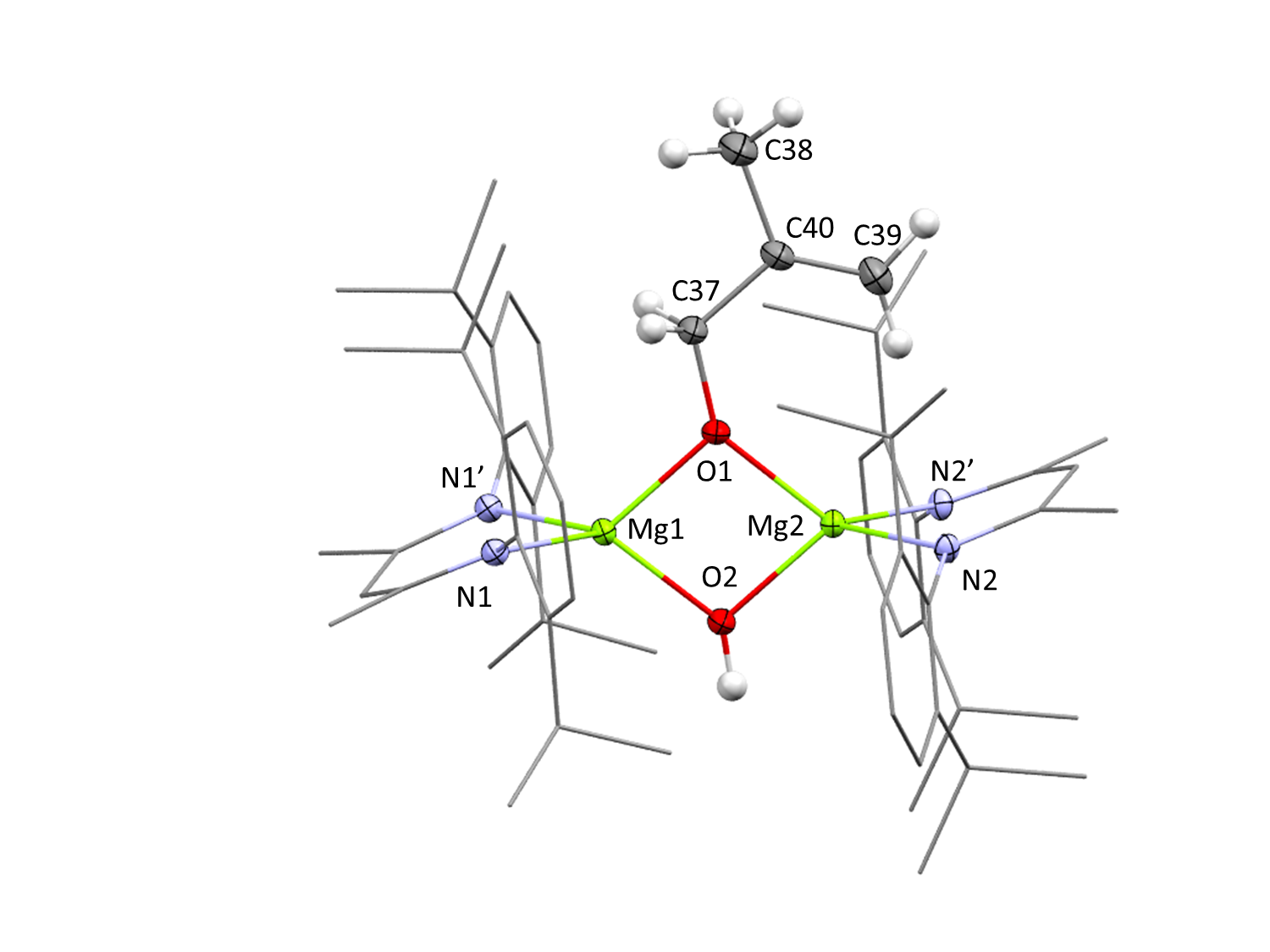


**Figure S100:** Structure of [^Dipp^NacnacMg(OH)(OCH_2_C(CH_2_)(CH_3_))Mg^Dipp^Nacnac] **9** obtained at room temperature from C_6_D_6_ overnight, minor disorder component is not shown. Selected hydrogen atoms are shown at fixed radii. Selected parts of the ligand framework shown as wireframe. Thermal ellipsoids set to 50% probability. Selected bond lengths (Å) and angles (°) Mg1-O1 1.983(2), Mg1-O2 1.965(2), Mg2-O1 1.970(2), Mg2-O2 1.958(2), Mg1-Mg2 3.0102(8), O1-C37 1.400(3), C37-C40 1.496(3), C38-C40 1.518(4) and C39-C40 1.318(4), Mg1-O1-Mg2 99.17(8) and Mg1-O2-Mg2 100.22(9).


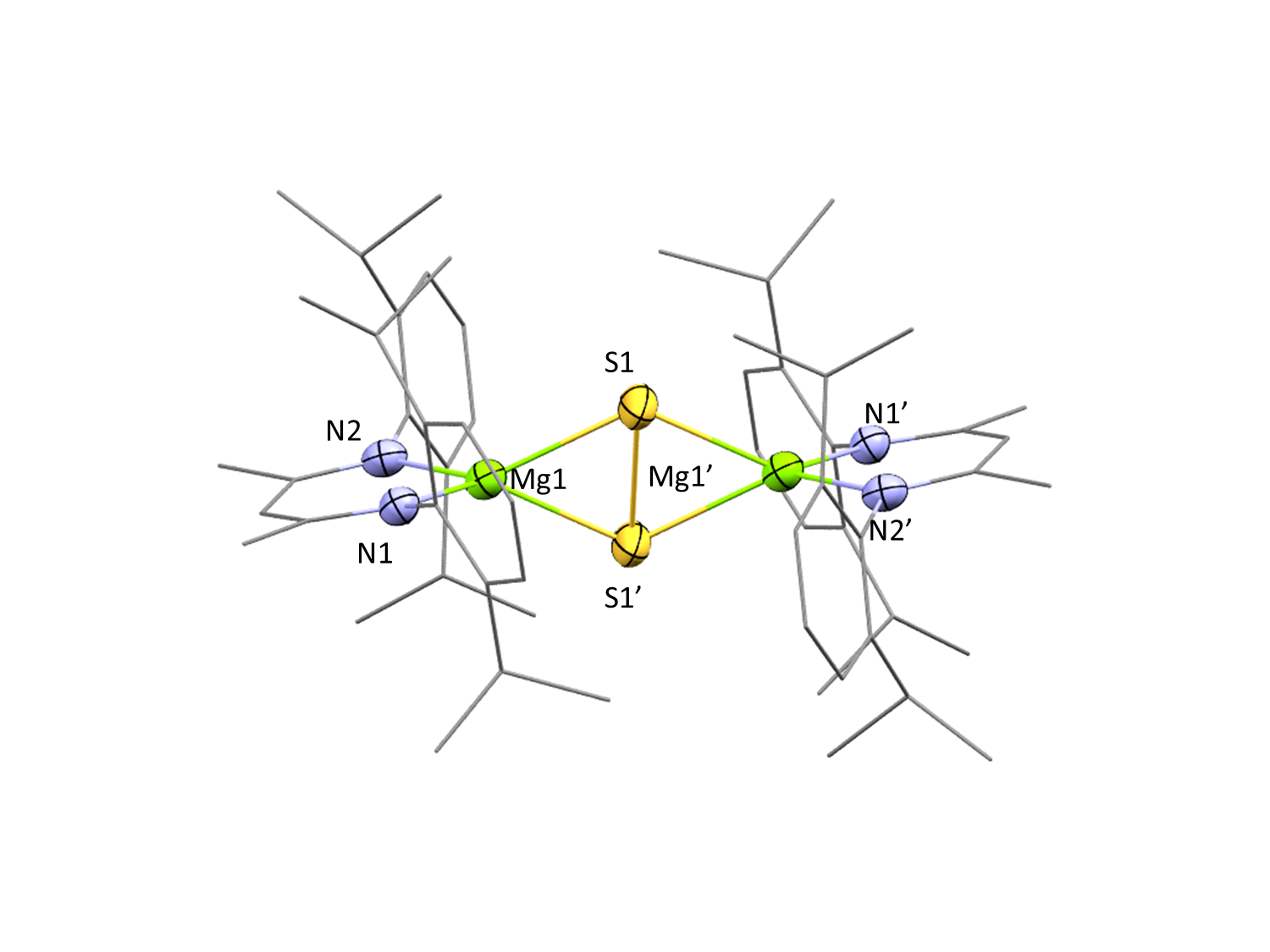


**Figure S101:** Structure of solvate free [^Dipp^NacnacMg_2_S_2_] **12** obtained from toluene with the addition of hexane as an antisolvent and stored at -30 °C overnight. Selected parts of the ligand framework shown as wireframe. Thermal ellipsoids set to 50% probability. Selected bond lengths (Å) and angles (°) Mg1-S1 2.462(2), Mg1-S1’ 2.336(2), Mg1-Mg1’ 4.5456(4), S1-S1’ 2.303(4) and Mg1-S1-Mg1’ 122.69(8).


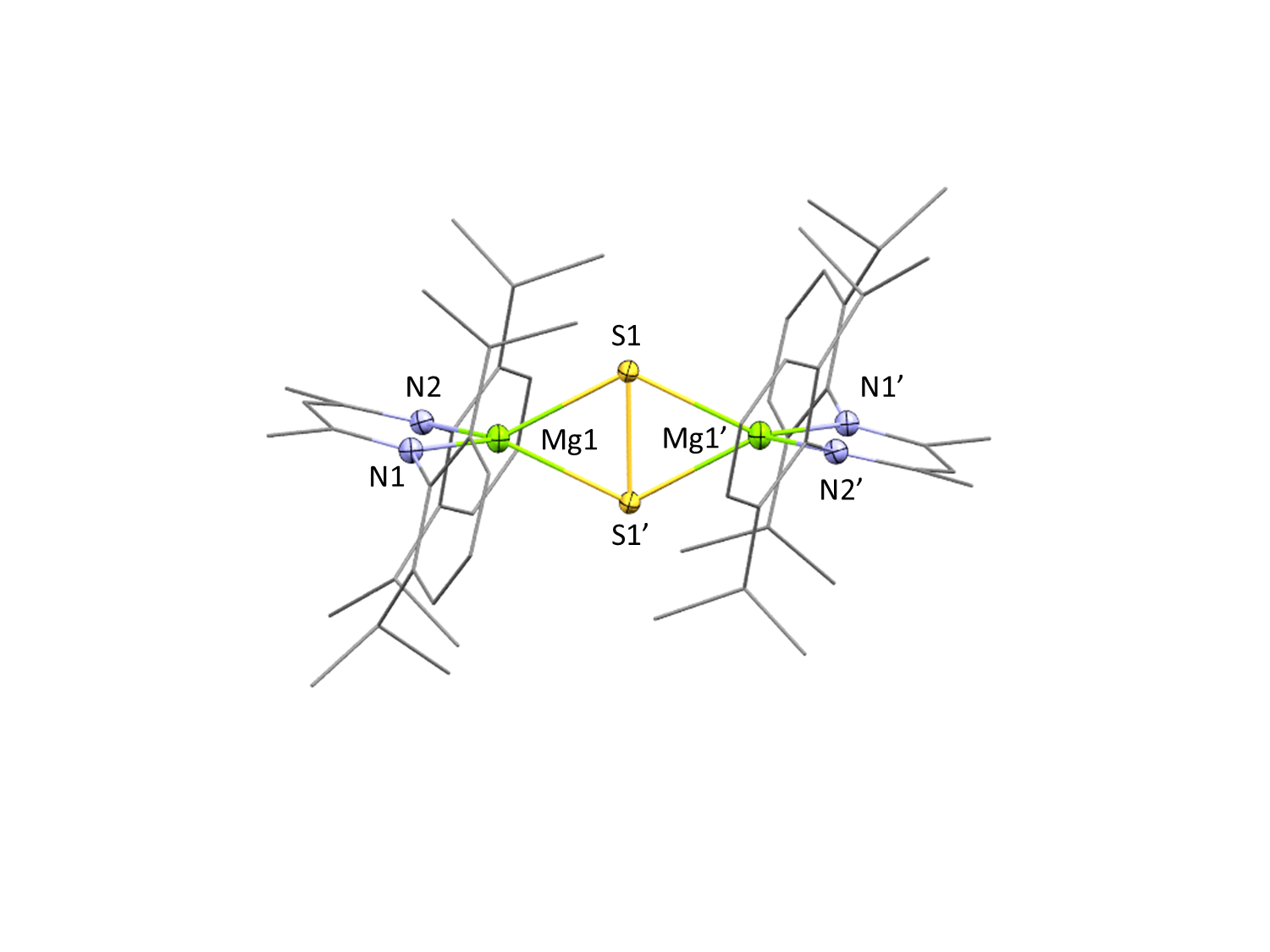


**Figure S102**: Structure of hexane solvate [^Dipp^NacnacMg_2_S_2_] **12** obtained from toluene with the addition of hexane as an antisolvent and stored at -30 °C ovenight. Selected parts of the ligand framework shown as wireframe. Thermal ellipsoids set to 50% probability. Selected bond lengths (Å) and angles (°) Mg1-S1 2.3710(15), Mg1-S1’ 2.4020(16), Mg1-Mg1’ 4.2106(7), S1-S1’ 2.167(4) and Mg1-S1-Mg1’ 126.01(8).


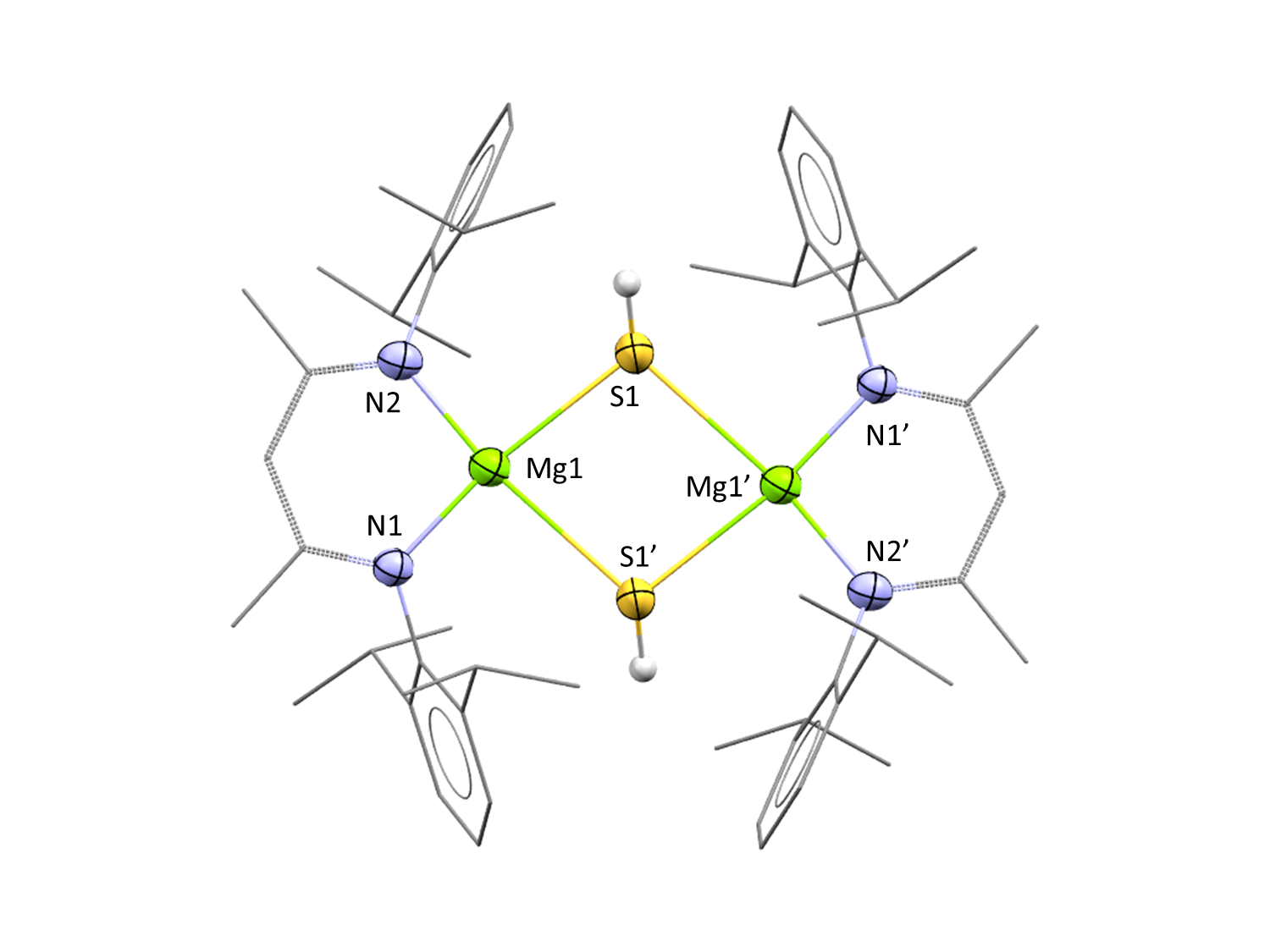


**Figure S103:** Structure of the bis-thiolate impurity [^Dipp^NacnacMgSH]_2_ which co-crystallised (2.5% approx.) with **12**, obtained from toluene with the addition of hexane as an antisolvent and stored at -30 °C overnight. Selected parts of the ligand framework shown as wireframe. Thermal ellipsoids set to 50% probability. Selected bond lengths (Å) and angles (°) Mg1-S1 2.438(5), Mg1-S1’ 2.482(5), Mg1-Mg1’ 4.2531(7), S1-S1’ 2.167(4) and Mg1-S1-Mg1’ 119.6(3). The crystal structure of the analogous (*^i^*^PrDipp^NacnacMgSH]_2_ Nacnac magnesium bis-thiolate has previously been reported, also as a degradation product.^[39]^


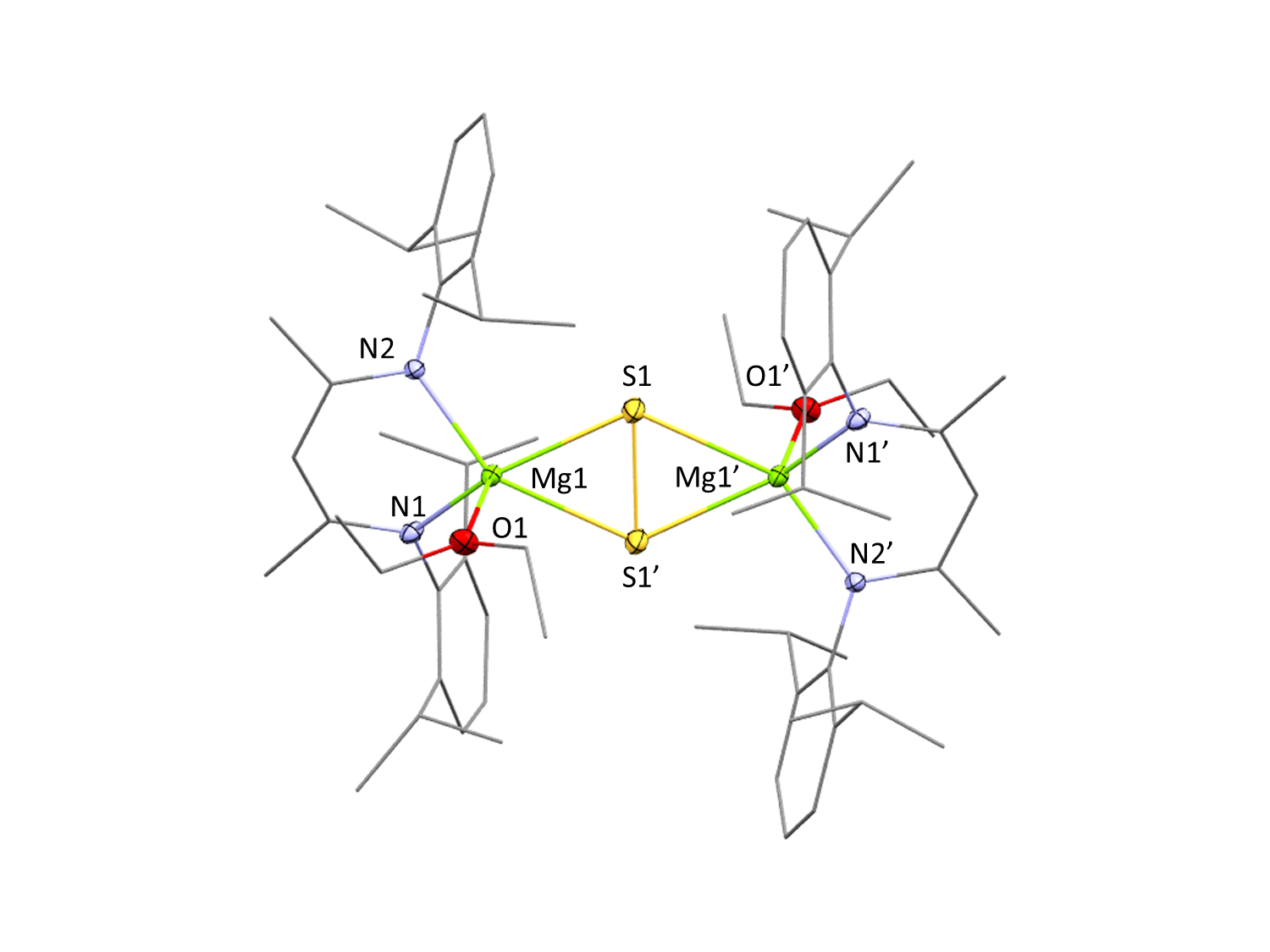


**Figure S104:** Structure of [(^Dipp^NacnacMgS)_2_•(OEt_2_)_2_] **12**•(Et_2_O)_2_ obtained as the minor crystalline product (16%) from the reaction of [^Dipp^NacnacMg]_2_ with propylene sulfide (2 eq) in diethyl ether at room temperature overnight. Selected parts of the ligand framework shown as wireframe. Thermal ellipsoids set to 50% probability. Selected bond lengths (Å) and angles (°) Mg1-S1 2.5459(15), Mg1-S1’ 2.4959(15), Mg1-Mg1’ 4.5456(4) S1-S1’ 2.181(3) and Mg1-S1-Mg1’ 128.73(7).

**
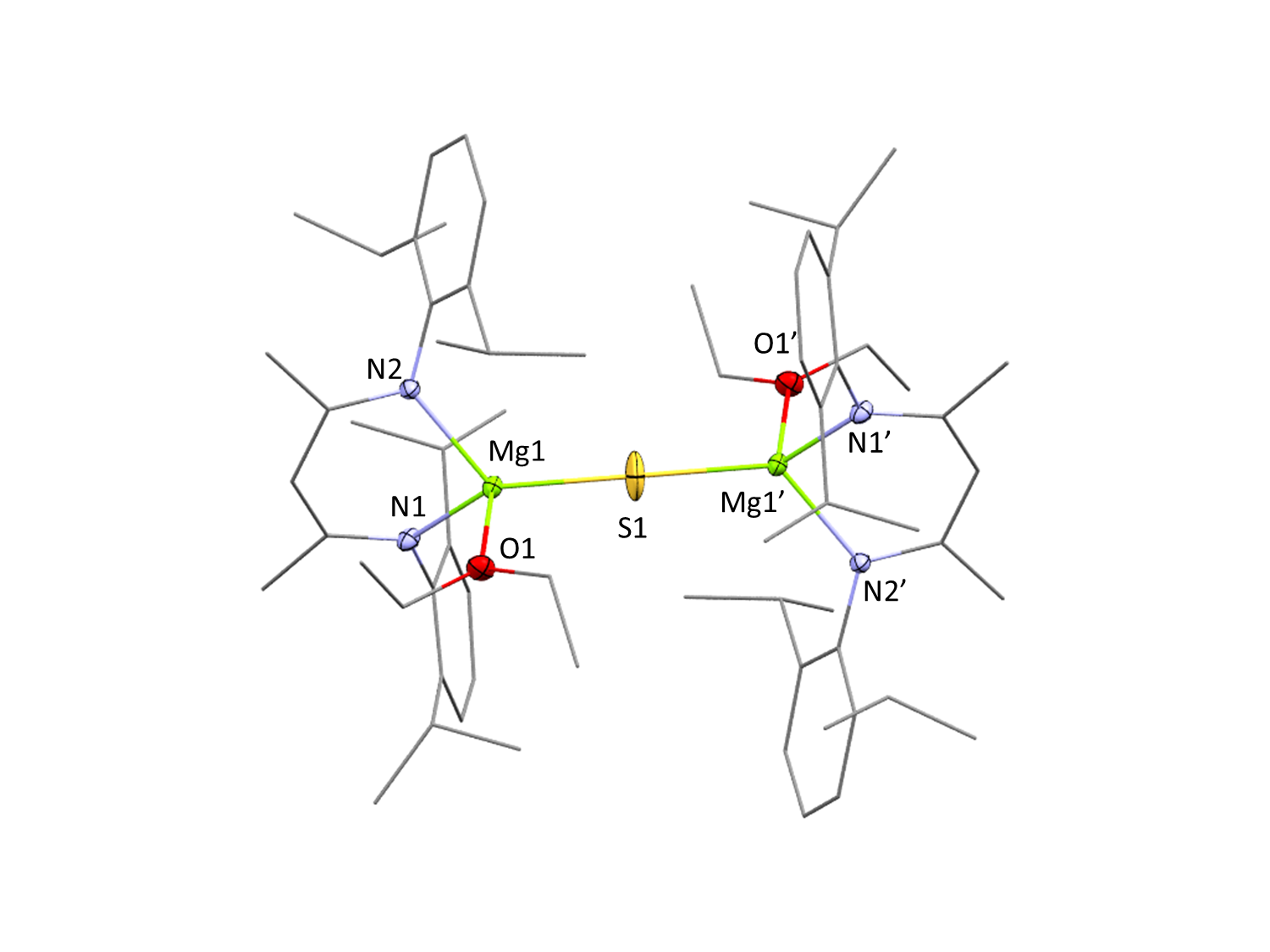
**

**Figure S105:** Structure of [(^Dipp^NacnacMg)_2_S•(OEt_2_)_2_] **13**•(Et_2_O)_2_ obtained as the major crystalline product (84%) from the reaction of [^Dipp^NacnacMg]_2_ with propylene sulfide (2 eq) in diethyl ether at room temperature overnight. Selected parts of the ligand framework shown as wireframe. Thermal ellipsoids set to 50% probability. Selected bond lengths (Å) and angles (°) Mg1-S1 2.2728(3) and Mg1-S1-Mg1’ 180.0.


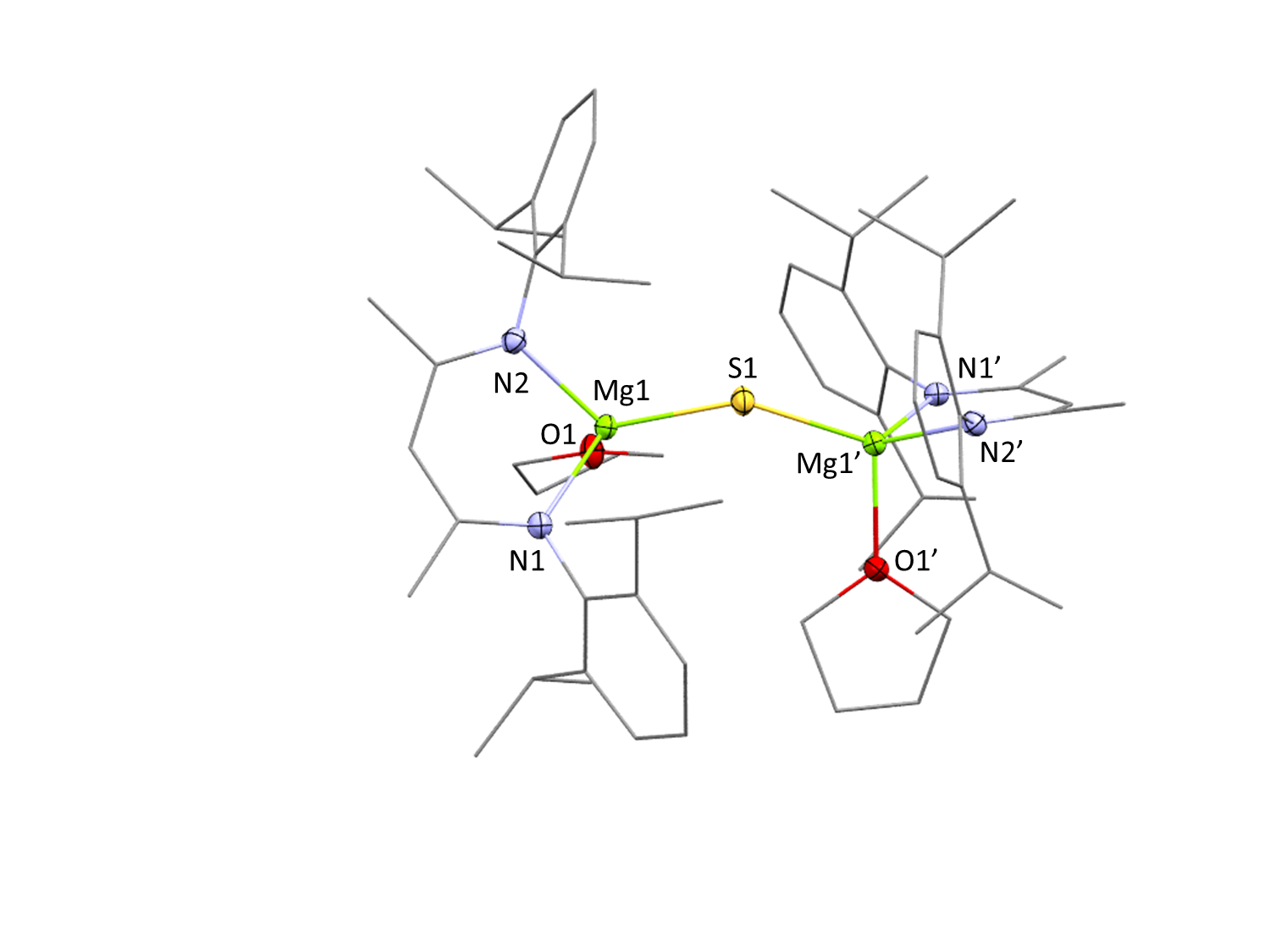


**Figure S106:** Structure of [^Dipp^NacnacMg)_2_S•THF_2_] **13**•THF_2_ obtained from obtained from toluene with the addition of hexane as an antisolvent and stored at -30 °C over three days, minor disorder component is not shown. Selected parts of the ligand framework shown as wireframe. Thermal ellipsoids set to 50% probability. Selected bond lengths (Å) and angles (°) Mg1-S1 2.2902(3), Mg1'-S1 2.2903(3) and Mg1-S1-Mg1’ 140.11(2).


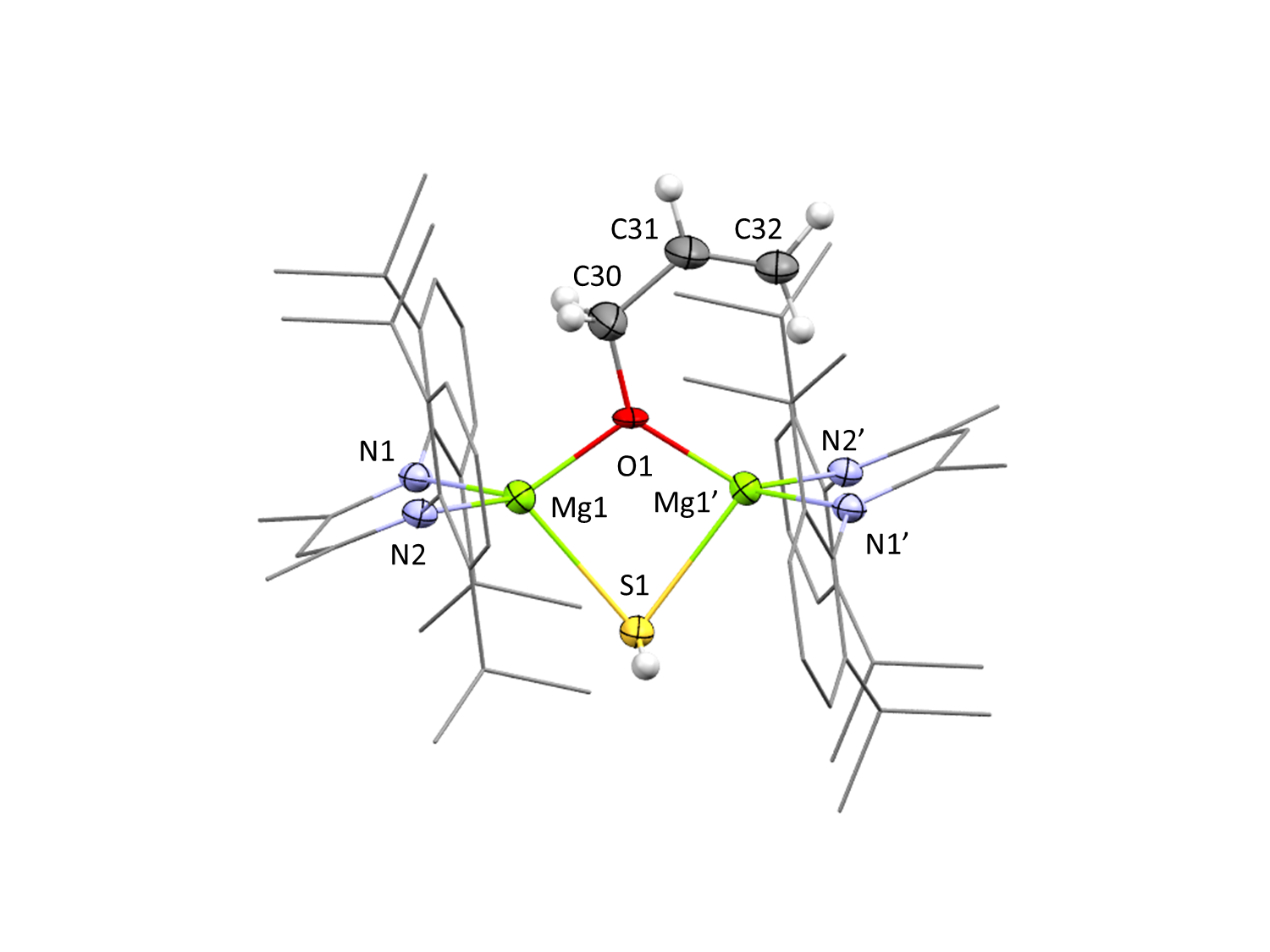


**Figure S107:** Structure of [^Dipp^NacnacMg(SH)(OCH_2_CHCH_2_)Mg^Dipp^Nacnac] **14** obtained from *d*_8_-toluene/hexane at -30 °C over three days. Selected parts of the ligand framework shown as wireframe. Thermal ellipsoids set to 50% probability. Selected bond lengths (Å) and angles (°) Mg1-O1 1.927(4), Mg1'-O1 1.918(4), Mg1-S1 2.553(2), Mg1’-S1 2.551(2), Mg1-Mg1’ 3.1906(17), Mg1-O1-Mg1’ 112.1(2) and Mg1-S1-Mg1’ 77.37(7).

## **References**

[7] R. Lalrempuia, A. Stasch, A. Stasch, C. Jones, "The reductive disproportionation of CO_2_ using a magnesium(I) complex: Analogies with low valent f-block chemistry", *Chem. Sci.*, **2013**, *4*, 4383–4388, 10.1039/c3sc52242c.

[13] J. Maurer, L. Klerner, J. Mai, H. Stecher, S. Thum, M. Morasch, J. Langer, S. Harder, "Redox-active inverse crowns for small molecule activation", *Nat. Chem.,* **2025**, *17*, 703–709, 10.1038/s41557-024-01724-5.

[27] M. H. Chisholm, J. Gllucci, K. Phomphrai, "Coordination Chemistry and Reactivity of Monomeric Alkoxides and Amides of Magnesium and Zinc Supported by Diiminato Ligand CH(CMeNC_6_H_3_-2,6-^i^Pr_2_)_2_. A Comparative Study", *Inorg. Chem.*, **2002**, *41*, 2785–2794, 10.1021/ic020148e.

[29] W. Ren, D. Gu, "An Azobenzenyl Anion Radical Complex of Magnesium: Synthesis, Structure, and Reactivity Studies", *Inorg. Chem.*, **2016**, *55*, 11962–11970, 10.1021/acs.inorgchem.6b02114.

[34] P. Idzko, J. P. Wagner, "Sulfur Monoxide Release and Capture: A Computational Study", *Eur. J. Org. Chem.,* **2023**, *26*, 10.1002/ejoc.202201370.

[35] Y. Steudel, R. Steudel, M. W. Wong, "The thermal decomposition of thiirane: a mechanistic study by ab initio MO theory", *Chem. Eur. J.,* **2002**, *8*, 217–228, 10.1002/1521-3765(20020104)8:1<217::aid-chem217>3.0.co;2-0.

[39] S. Burnett, R. Ferns, D. B. Cordes, A. M. Z. Slawin, T. van Mourik, A. Stasch, "Low-Coordinate Magnesium Sulfide and Selenide Complexes", *Inorg. Chem.*, **2023**, *62*, 16443–16450, 10.1021/acs.inorgchem.3c02132.

[42] S. P. Green, C. Jones, A. Stasch, "Stable adducts of a dimeric magnesium(I) compound", *Angew. Chem. Int. Ed.,* **2008**, *47*, 9079–9083, 10.1002/anie.200803960.

[44] M. Stender, R. J. Wright, B. E. Eichler, J. Prust, M. M. Olmstead, H. W. Roesky, P. P. Power, "The synthesis and structure of lithium derivatives of the sterically encumbered β-diketiminate ligand [{(2,6-Pr^i^_2_H_3_C_6_)N(CH_3_)C}_2_CH]–, and a modified synthesis of the aminoimine precursor", *J. Chem. Soc., Dalton Trans.*, **2001**, 3465–3469, 10.1039/b103149j.

[45] S. J. Bonyhady, C. Jones, S. Nembenna, A. Stasch, A. J. Edwards, G. J. McIntyre, "β-Diketiminate-Stabilized Magnesium(I) Dimers and Magnesium(II) Hydride Complexes: Synthesis, Characterization, Adduct Formation, and Reactivity Studies", *Chem. Eur. J.*, **2010**, *16*, 938–955, 10.1002/chem.200902425.

[46] R. Lalrempuia, C. E. Kefalidis, S. J. Bonyhady, B. Schwarze, L. Maron, A. Stasch, C. Jones, "Activation of CO by Hydrogenated Magnesium(I) Dimers: Sterically Controlled Formation of Ethenediolate and Cyclopropanetriolate Complexes", *J. Am. Chem. Soc.*, **2015**, *137*, 8944–8947, 10.1021/jacs.5b06439.

[47] J. Hicks, M. Juckel, A. Paparo, D. Dange, C. Jones, "Multigram Syntheses of Magnesium(I) Compounds Using Alkali Metal Halide Supported Alkali Metals as Dispersible Reducing Agents", *Organometallics*, **2018**, *37*, 4810–4813, 10.1021/acs.organomet.8b00803.

[48] T. J. Yue, M. C. Zhang, G. G. Gu, L. Y. Wang, W. M. Ren, X. B. Lu, "Precise Synthesis of Poly(thioester)s with Diverse Structures by Copolymerization of Cyclic Thioanhydrides and Episulfides Mediated by Organic Ammonium Salts", *Angew. Chem. Int. Ed.*, **2019**, *131*, 628–633, 10.1002/ange.201812135.

[49] G. M. Sheldrick, "A short history of ShelX", *Acta Cryst.*, **2008**, *A64*, 112–122, 10.1107/s0108767307043930.

[50] G. M. Sheldrick, "ShelXT – Integrated space-group and crystal-structure determination", *Acta Cryst.*, **2015**, *A71*, 3–8, 10.1107/s2053273314026370.

[51] G. M. Sheldrick, "Crystal structure refinement with ShelXL", *Acta Cryst.*, **2015**, *C71*, 3–8, 10.1107/s2053229614024218.

[52] L. J. Bourhis, O. V. Dolomanov, R. J. Gildea, J. A. K. Howard, H. Puschmann, "The anatomy of a comprehensive constrained, restrained refinement program for the modern computing environment – Olex2 dissected", *Acta Cryst.*, **2015**, *71*, 59–75, 10.1107/s2053273314022207.

[53] O. V. Dolomanov, L. J. Bourhis, R. J. Gildea, J. A. K. Howard, H. Puschmann, "OLEX2: A complete structure solution, refinement and analysis program", *J. Appl. Cryst.*, **2009**, *42*, 339–341, 10.1107/s0021889808042726.
